# Supplementary material for: Enabling long-distance hydrogen spillover in nonreducible metal-organic frameworks for catalytic reaction
Source: Nat Commun. 2024 Jul 24;15:6263. doi: 10.1038/s41467-024-50706-1 (PMC11269641; doi:10.1038/s41467-024-50706-1)
Supplement: Supplementary file 1 — Supplementary Information for Enabling long-distance hydrogen spillover in nonreducible metal-organic frameworks for catalytic reaction [file 41467_2024_50706_MOESM1_ESM.pdf]

Supplementary Information

Enabling long-distance hydrogen spillover in nonreducible metal-organic frameworks for catalytic reaction

Xiao-Jue Bai[1], Caoyu Yang[1,2] and Zhiyong Tang[1,2*]

[1]Chinese Academy of Sciences (CAS) Key Laboratory of Nanosystem and Hierarchy Fabrication, National Center for Nanoscience and Technology, Beijing 100190, China.

[2]University of Chinese Academy of Sciences; Beijing 100049, P. R. China.

[*]e-mail: zytang@nanoctr.cn

This file includes:

Supplementary Methods

Supplementary Figures 1 to 83

Supplementary Tables S1 to S7

Supplementary Methods

Synthesis of Cu-MOF-2/Pt. 526.5 mg $Cu(NO_3)_2·3H_2O$, 362 mg 1,4-benzenedicarboxylic acid and 43.5 mL dimethylformamide (DMF) were mixed in a 250 mL round-bottom flask and refluxed at 100°C for 24 h. The resulting powder was collected by centrifugation at 7000 rpm. Finally, the solid was washed consecutively three times with DMF followed by additional three times with CH_3OH, and then dried at 80°C for 12 h.

10 mg Cu-MOF-2 was redispersed in 4 mL ethanol, and then 2 mL Pt nanoparticles (NPs) solution (1.2 mM) was added drop by drop under stirring. Afterward, the solution was left stirred for 2 h at room temperature. As-synthesized Cu-MOF-2/Pt was centrifuged at 8000 rpm for 3 min and dried at 80°C in a vacuum.

Synthesis of Fe-MIL-101/Pt. 270 mg $FeCl_3·6H_2O$ and 166 mg 1,4-benzenedicarboxylic acid were dissolved in 5 mL DMF. After that, the solution was treated under ultrasonic condition for 20 min. Subsequently, the solution was transferred into a 25 mL Teflon-lined stainless-steel autoclave and heated at 150°C for 12 h. After the reaction, the product was collected by centrifugation at 7000 rpm. Finally, the solid was washed consecutively three times with DMF followed by additional three times with CH_3OH, and then dried at 80°C for 12 h.

10 mg Fe-MIL-101 was redispersed in 4 mL ethanol, and then 2 mL Pt NPs solution (1.2 mM) was added drop by drop under stirring. Subsequently, the solution was left stirred for 2 h at room temperature. As-synthesized Fe-MIL-101/Pt was centrifuged at 7000 rpm for 3 min and dried at 80°C in a vacuum.

Synthesis of Co-ZIF-67/Pt. 145.5 mg $Co(NO_3)_2{\cdot}6H_2O$ was dissolved in 5 mL methanol, meanwhile 0.164 g 2-methylimidazole was dissolved in another 5 mL methanol. The metal ion solution was then rapidly added to the organic ligand solution, and the mixed solution was left to stand for 24 h. Finally, the obtained powder was washed with methanol three times *via* centrifugation at 8,000 rpm for 5 min and dried at 80°C in a vacuum.

10 mg Co-ZIF-67 was redispersed in 4 mL ethanol, and then 2 mL Pt NPs solution (1.2 mM) was added drop by drop under stirring. Subsequently, the solution was left stirred for 2 h at room temperature. As-synthesized Co-ZIF-67/Pt was centrifuged at 8000 rpm for 3 min and dried at 80°C in a vacuum.

Synthesis of Zr-UiO-66/Pt. 25 mg 1,4-benzenedicarboxylic was dissolved in 5 mL DMF, meanwhile 33.4 mg $ZrCl_4$ and 0.7 mL acetic acid were dissolved in another 5 mL DMF. The above solutions were transferred into a Teflon lined stainless steel autoclave in 120°C oven for 24 h. The resulting powder was collected by centrifugation at 10000 rpm for 3 min, washed twice with DMF and twice with methanol, and then dried at 80°C in a vacuum.

10 mg Zr-UiO-66 was redispersed in 4 mL ethanol, and then 2 mL Pt NPs solution (1.2 mM) was added drop by drop under stirring. Subsequently, the solution was left stirred for 2 h at room temperature. As-synthesized Zr-UiO-66/Pt was centrifuged at 10000 rpm for 3 min and dried at 80°C in a vacuum.

Synthesis of Zn-ZIF-8/Pt. 148 mg $Zn(NO_3)_2{\cdot}6H_2O$ was dissolved in 5 mL methanol, meanwhile 0.164 g 2-methylimidazole was dissolved in another 5mL methanol. The

metal ion solution was then rapidly added to the organic ligand solution, and the mixed solution was left to stand for 24 h. Finally, the obtained powder was washed with methanol three times *via* centrifugation at 8,000 rpm for 5 min and dried at 80°C in a vacuum.

10 mg Zn-ZIF-8 was redispersed in 4 mL ethanol, and then 2 mL Pt NPs solution (1.2 mM) was added drop by drop under stirring. Subsequently, the solution was left stirred for 2 h at room temperature. As-synthesized Zn-ZIF-8/Pt was centrifuged at 8000 rpm for 3 min and dried at 80°C in a vacuum.

General Characterization. Thermogravimetric analysis (TGA) was recorded by NETZSCH STA 449F3 and the heating rate was 10°C/min. Morphologies of samples were characterized by scanning electron microscopy (SEM, Hitachi S4800), transmission electron microscopy (TEM) and high-resolution TEM (HRTEM, Tecnai G2 20 S-TWIN). Scanning transmission electron microscopy (STEM) imaging and energy-dispersive X-ray spectroscopy (EDS) elemental mapping were carried out on Tecnai G2 F20 S-TWIN. The crystallographic information was analyzed by X-ray diffraction (XRD, D/MAX-TTRIII(CBO)) equipped with a Cu Kα radiation source (λ = 1.5406 Å) operating at 50 kV and 300 mA. The organic groups in ZIFs materials were characterized by Fourier transform infrared spectroscopy (FT-IR, Thermo Scientific Nicolet IS50). The isothermal adsorption curve of the samples was determined by using nitrogen adsorption-desorption isotherms at 77 K (Micromeritics ASAP 2020 system). The composition of the materials was analyzed through quantitative X-ray photoelectron spectroscopy (XPS, THERMO VG ESCALAB250) that gave

information about the surface electronic states of the elements. Nuclear magnetic resonance (NMR) spectra were recorded at 400 MHz with an Avance III Bruker Corporation instrument.

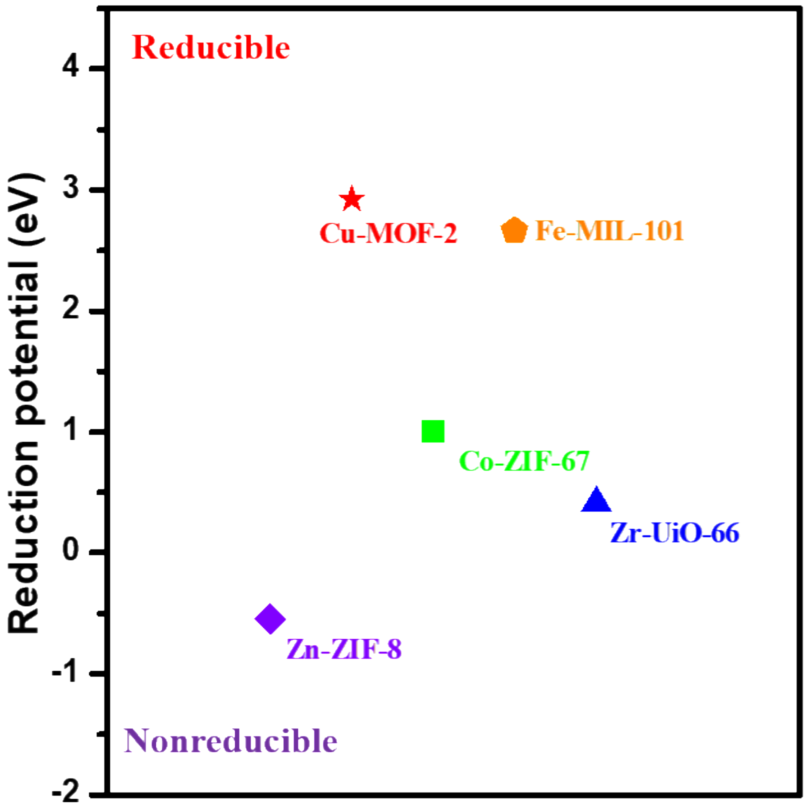

Supplementary Fig. 1 Calculated reduction potential values obtained from the quantum chemistry method for different MOF clusters.[a]

According to the literature,[a] the redox ability of different MOFs (metal clusters) is evaluated by the reduction potential energy ($E_{reduction}$). Accurate adiabatic electronic affinity (E_A) values are obtained from the quantum chemistry calculation, and the value of E_A is the opposite of the reduction energy ($E_{reduction}$) that is defined as the energy difference between MOFs in neutral and anion states ($E_{reduction} = -E_A = E_{neutral} - E_{anion}$). As shown in Supplementary Fig. 1, the $E_{reduction}$ values of several typical MOFs are quite different and follow the order: $E_{reduction}$ (Cu-MOF-2) > $E_{reduction}$ (Fe-MIL-101) > $E_{reduction}$ (Co-ZIF-67) > $E_{reduction}$ (Zr-UiO-66) > $E_{reduction}$ (Zn-ZIF-8). Generally, the $E_{reduction}$ of MOFs depends mainly on the intrinsic chemical property of metal centers, whereas it is only slightly influenced by the coordination mode (or type of MOFs).

[a] Bai, X.-J., Zhai, X., Zhang, L.-Y., Fu, Y. & Qi, W. Site-directed reduction engineering within bimetal-organic frameworks for efficient size-selective catalysis. *Matter* **4**, 2919-2935, (2021).

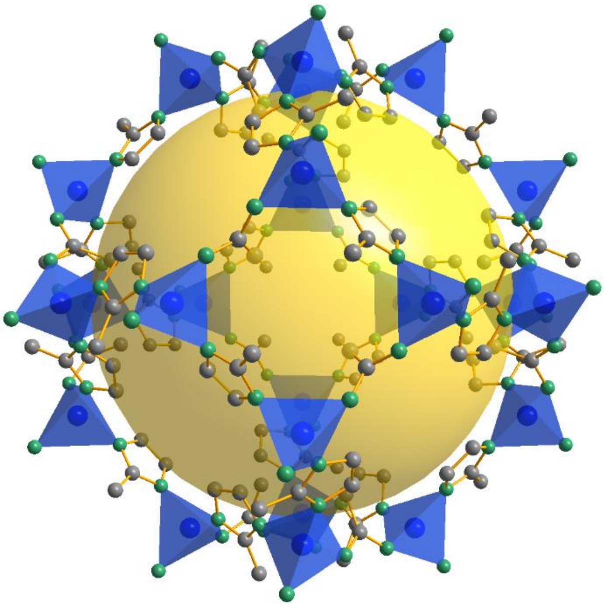

Supplementary Fig. 2 Crystal structure of Zn-based ZIF-8. Blue: Zn, green: N, gray: C, yellow sphere: cavity.

Detail: Zeolitic imidazolate framework-8 (ZIF-8). Molecular composition: $C_8H_{12}N_4 \cdot Zn$. Zn-ZIF-8 is a three-dimensional microporous structure composed of zinc ions [Zn^{2+}] as inorganic nodes connected to 4 organic ligands of 2-methylimidazole. CCDC number: 864309.

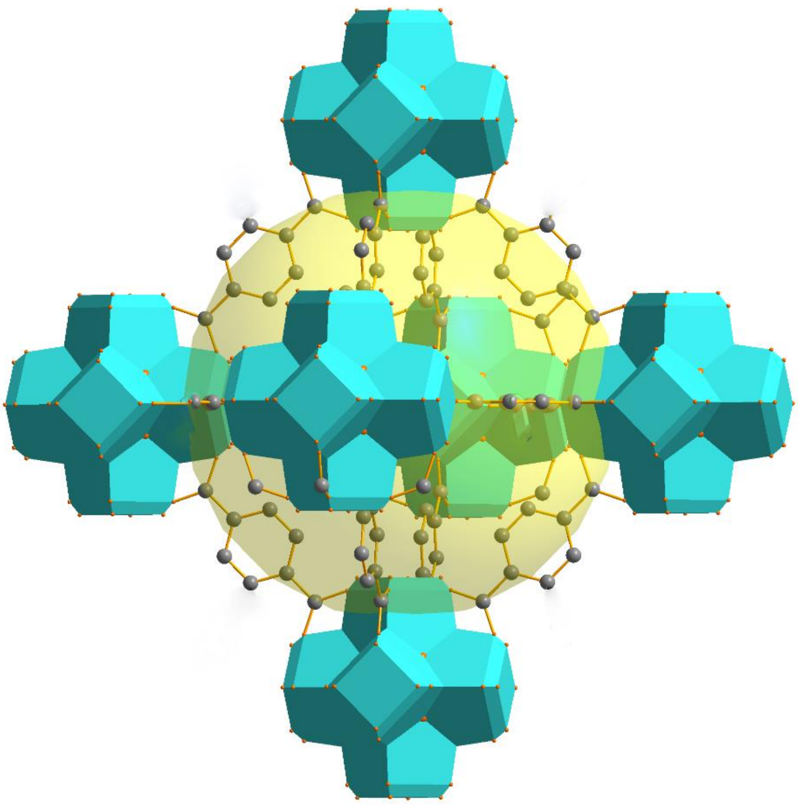

Supplementary Fig. 3 Crystal structure of Zr-based UiO-66. Cyan: Zr, red: O, gray: C, yellow sphere: cavity.

Detail: Universitetet i Oslo framework-66 (UiO-66). Molecular composition: $C_{48}H_{28}O_{32}Zr_6$.

The UiOs material was first synthesized by Professor Lillerud's group at the University of Oslo. Zr-UiO-66 is a three-dimensional microporous structure composed of zirconium clusters [$Zr_6O_4(OH)_4$] as inorganic nodes connected to 12 organic ligands of terephthalic acid. CCDC number: 1854712.

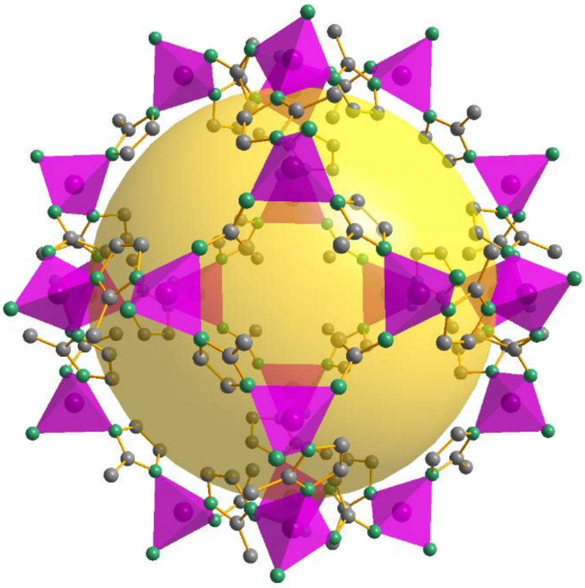

Supplementary Fig. 4 Crystal structure of Co-based ZIF-67. Pink: Co, green: N, gray: C, yellow sphere: cavity.

Detail: Zeolitic imidazolate framework-67 (ZIF-67). Molecular composition: $C_8H_{12}N_4Co$.

Co-ZIF-67 is a three-dimensional microporous structure composed of cobalt ions [Co^{2+}] as inorganic nodes connected to 4 organic ligands of 2-methylimidazole. CCDC number: 671073.

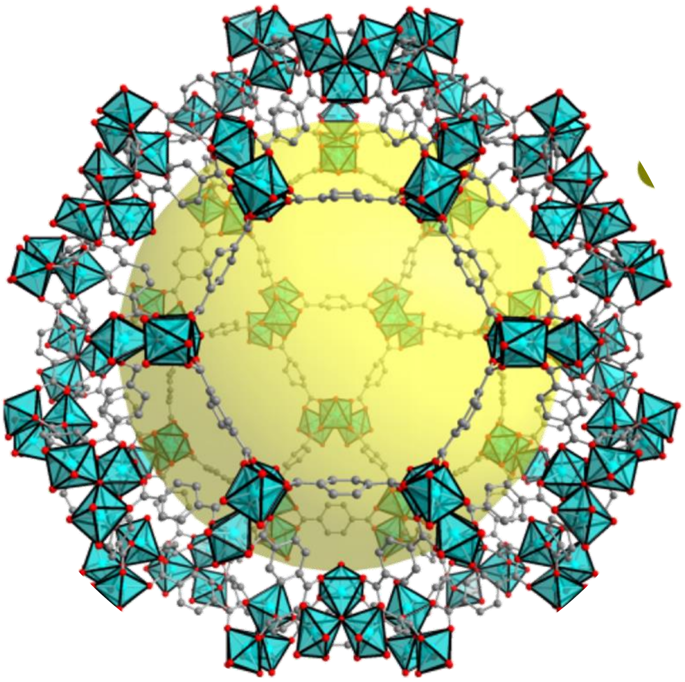

Supplementary Fig. 5 Crystal structure of Fe-based MIL-101. Blue: Fe, red: O, gray: C, yellow sphere: cavity.

Detail: Materials of Institute Lavoisier framework-101 (MIL-101). Molecular composition: $C_{24}H_{12}ClFe_3O_{13}$.

MILs material was first reported by Ferey's group at the Lavoisier Institute. Fe-MIL-101 is a mesoporous cage structure composed of iron trimers [Fe_3OCl] as inorganic nodes connected to 6 organic ligands of terephthalic acid. CCDC number: 605510.

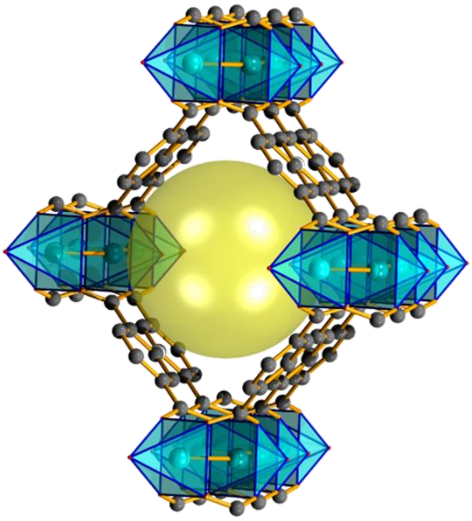

Supplementary Fig. 6 Crystal structure of Cu-based Cu-MOF-2. Cyan: Cu, red: O, gray: C, yellow sphere: cavity.

Detail: Cu-MOF-2. Molecular composition: $C_{16}H_8Cu_2O_8$.

Cu-MOF-2 is a one-dimensional microporous structure composed of copper dimers [Cu_2] as inorganic nodes connected to 4 organic ligands of terephthalic acid. CCDC number: 687690.

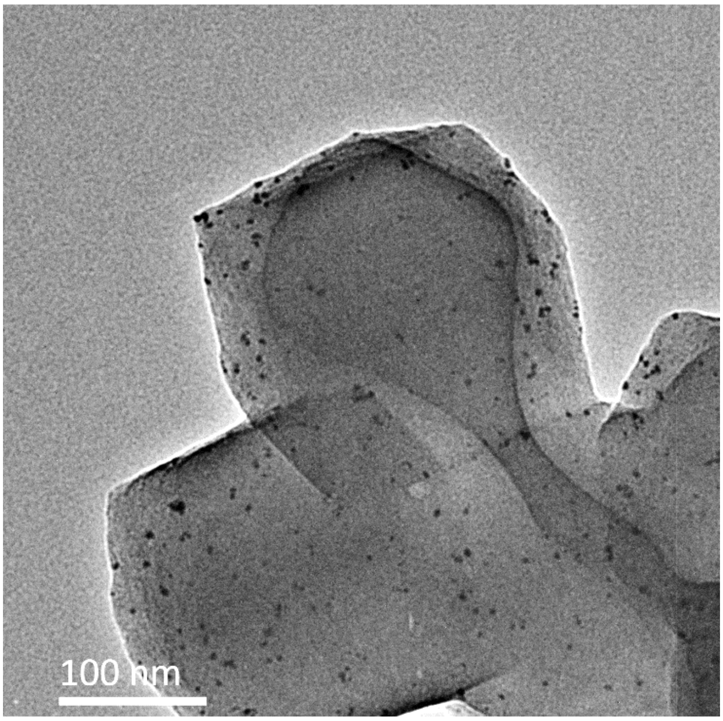

Supplementary Fig. 7 TEM image of Zn-ZIF-8/Pt.

As shown in Supplementary Fig. 7, the Zn-ZIF-8/Pt shows a dodecahedral shape in size of ~220 nm. And Pt nanoparticles are evenly distributed in the Zn-ZIF-8 crystal, forming the supported Zn-ZIF-8/Pt catalyst.

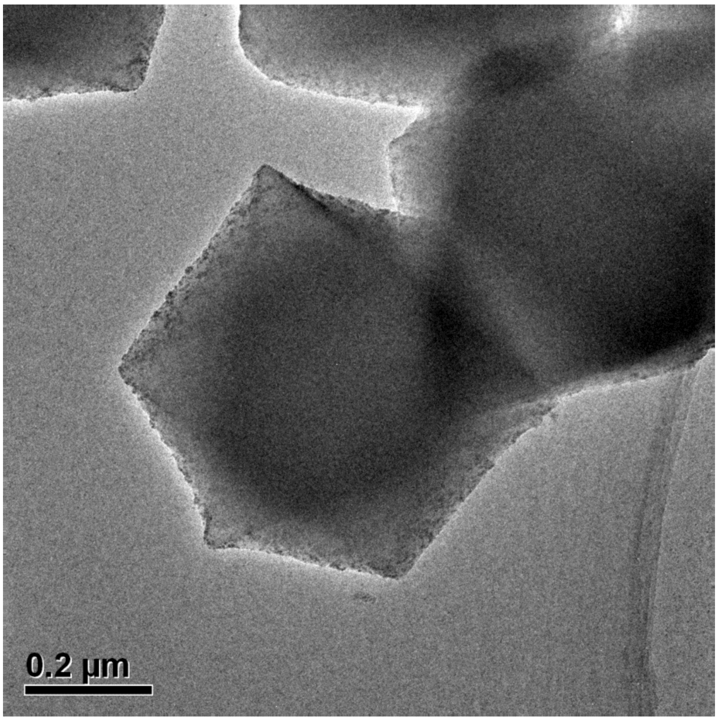

Supplementary Fig. 8 TEM image of Zr-UiO-66/Pt.

As shown in Supplementary Fig. 8, the Zr-UiO-66/Pt shows an octahedral shape in size of ~650 nm. And, Pt nanoparticles are evenly distributed in the Zr-UiO-66 crystal, forming the supported Zr-UiO-66/Pt catalyst.

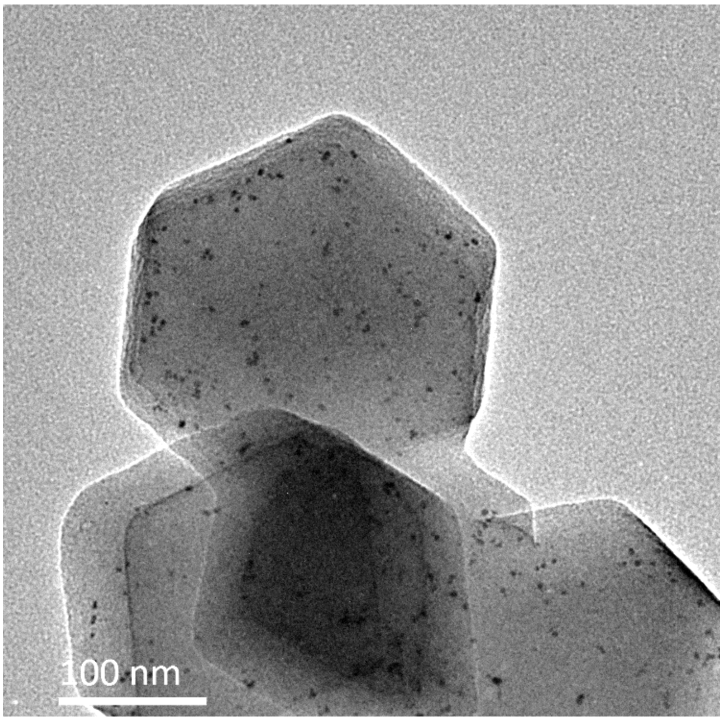

Supplementary Fig. 9 TEM image of Co-ZIF-67/Pt.

As shown in Supplementary Fig. 9, the Co-ZIF-67/Pt crystal shows a dodecahedral shape in size of ~240 nm. And, Pt nanoparticles are evenly distributed in the Co-ZIF-67 crystal, forming the supported Co-ZIF-67/Pt catalyst.

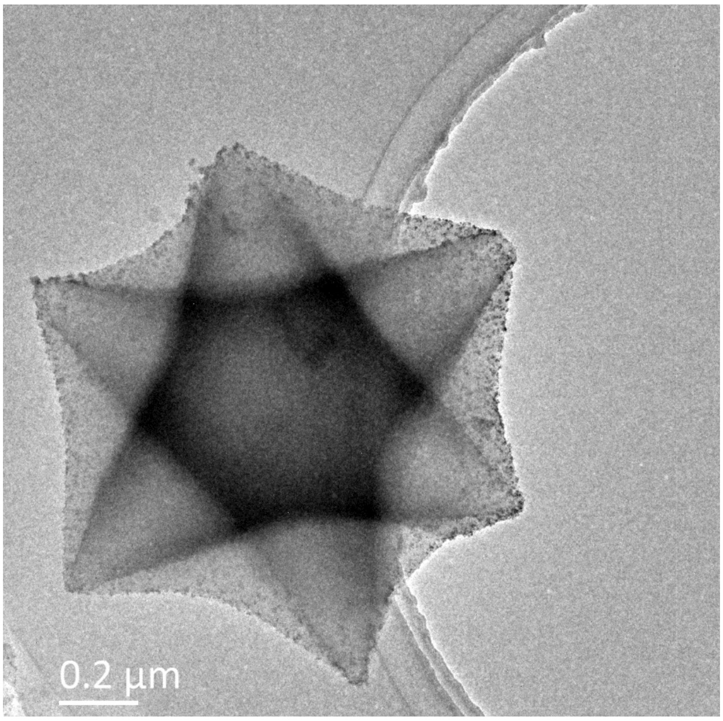

Supplementary Fig. 10 TEM image of Fe-MIL-101/Pt.

As shown in Supplementary Fig. 10, the Fe-MIL-101 shows an octahedral shape in size of ~1 μm. And, Pt nanoparticles are evenly distributed in the Fe-MIL-101 crystal, forming the supported Fe-MIL-101/Pt catalyst.

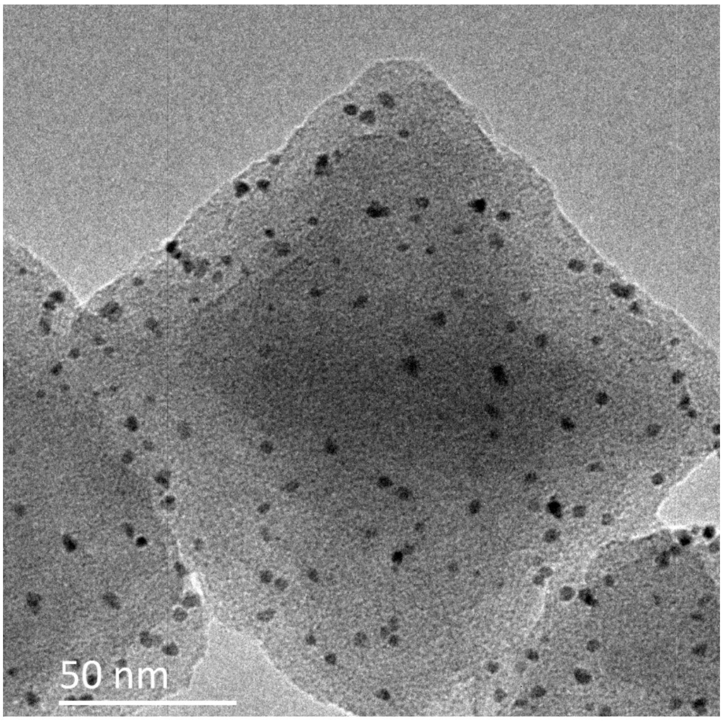

Supplementary Fig. 11 TEM image of Cu-MOF-2/Pt.

As shown in Supplementary Fig. 11, the Cu-MOF-2/Pt shows a hexahedral shape in size of ~150 nm. And, Pt nanoparticles are evenly distributed in the Cu-MOF-2 crystal, forming the supported Cu-MOF-2/Pt catalyst.

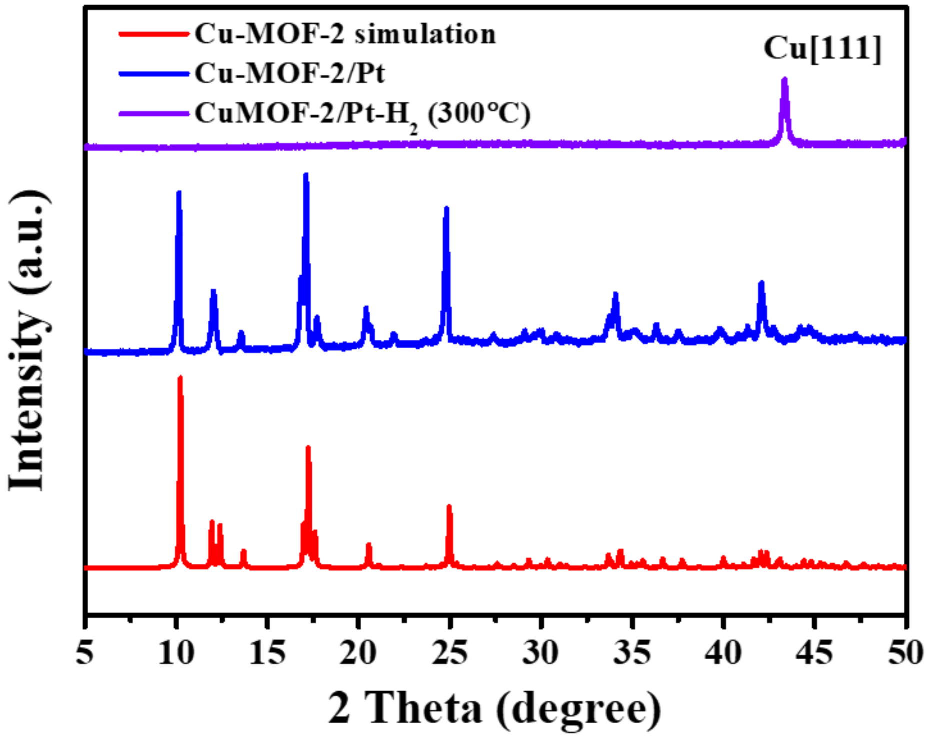

Supplementary Fig. 12 XRD patterns of Cu-MOF-2/Pt and Cu-MOF-2/Pt after heat treatment in flowing H_2 gas.

The characteristic diffraction peaks of Cu-MOF-2/Pt (blue curve) well match those of Cu-MOF-2 simulation (no.687690, CCDC), suggesting the structural stability of MOFs after loading Pt nanoparticles. After Cu-MOF-2/Pt is treated in H_2 at 300°C for 30 min, the diffraction peaks of Cu-MOF-2 completely disappear (purple curve) while a new peak attributed to the metallic Cu [111] appears, which proves that Cu^{2+} is reduced by hydrogen atoms *via* hydrogen spillover.

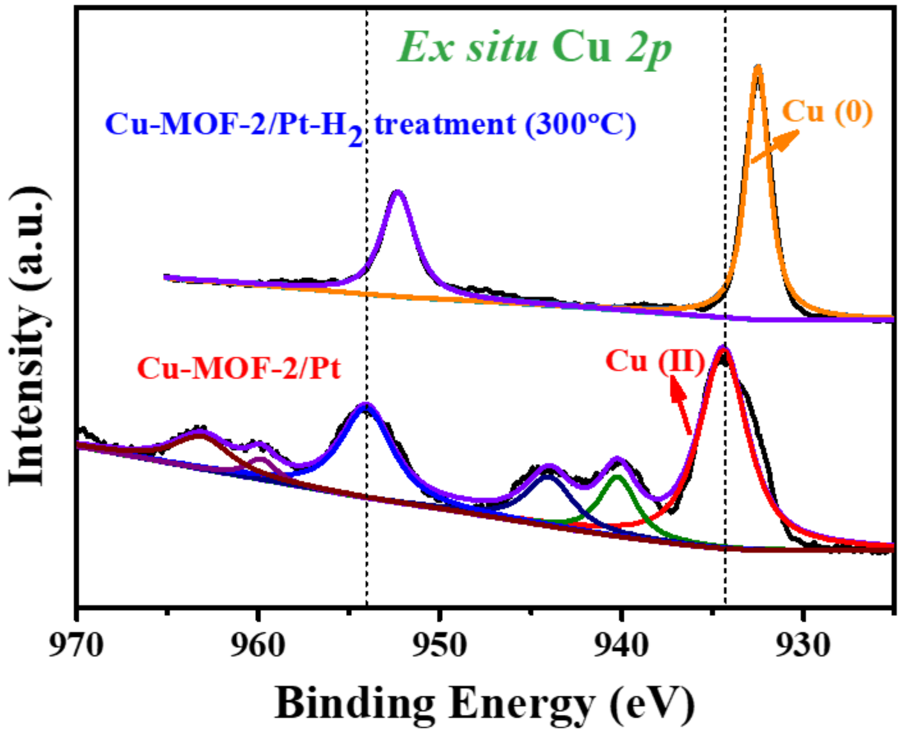

Supplementary Fig. 13 XPS spectra of Cu-MOF-2/Pt and Cu-MOF-2/Pt after heat treatment in flowing H_2 gas.

After Cu-MOF-2/Pt is treated in H_2 at 300°C, two obvious peaks appear at 932.5 and 952.3 eV (top curve), which is assigned to Cu^0. In good agreement with the XRD result (Supplementary Fig. 12), Cu^{2+} in Cu-MOF-2/Pt is reduced to metallic Cu by hydrogen atoms *via* hydrogen spillover.

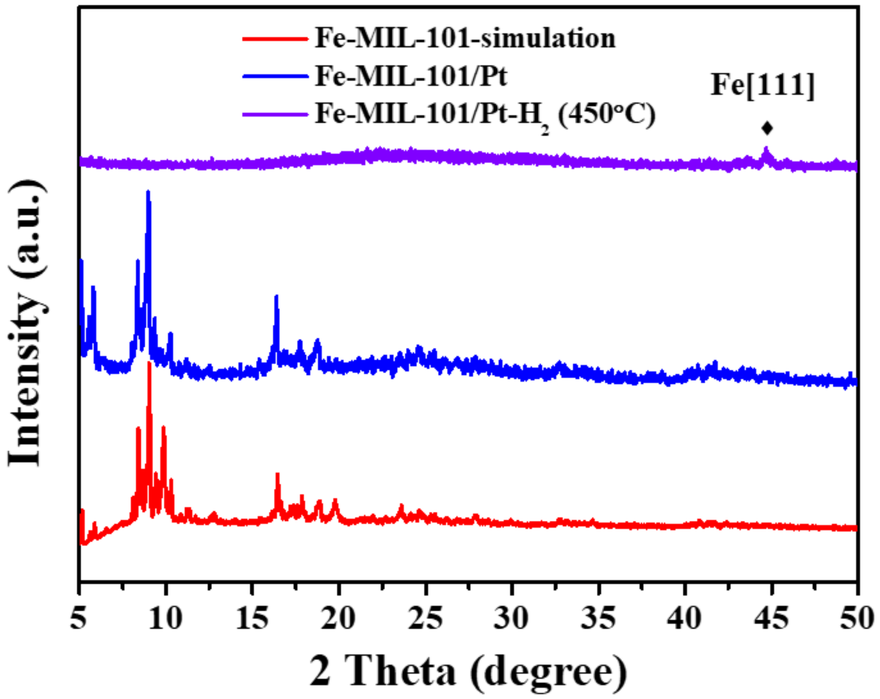

Supplementary Fig. 14 XRD patterns of Fe-MIL-101/Pt and Fe-MIL-101/Pt after heat treatment in flowing H_2 gas.

The characteristic diffraction peaks of Fe-MIL-101/Pt (blue curve) well match those of Fe-MIL-101 simulation, suggesting the structural stability of MOFs after loading Pt nanoparticles. After Fe-MIL-101/Pt is treated in H_2 at 450°C for 30 min, the diffraction peaks of Fe-MIL-101 completely disappear (purple curve) while a new peak attributed to the metallic Fe [111] appears, which proves that Fe^{3+} is reduced by hydrogen atoms *via* hydrogen spillover.

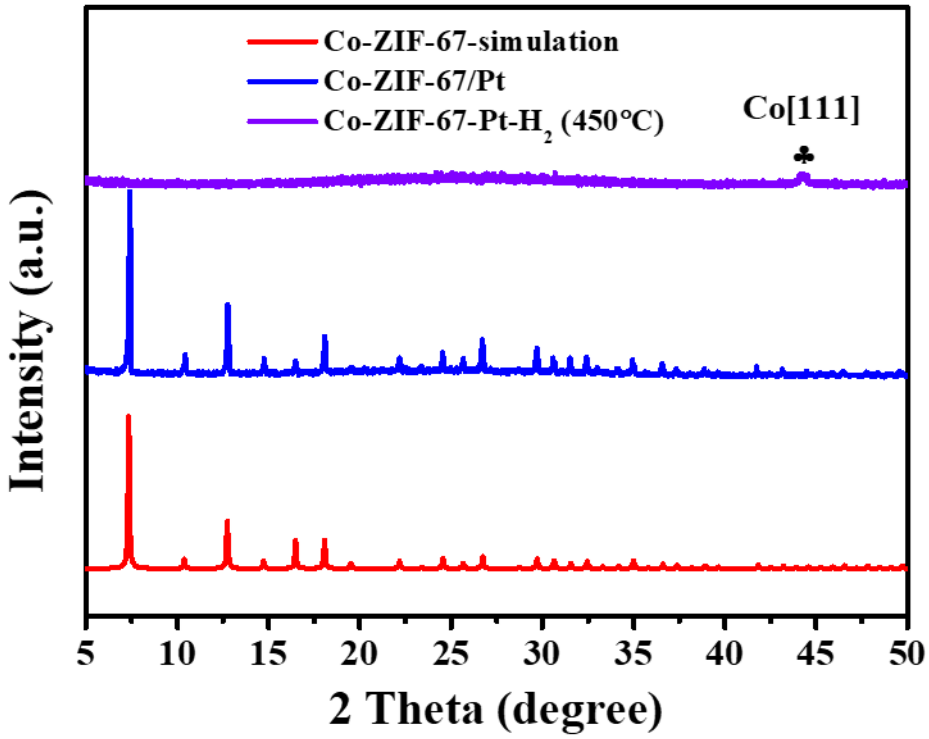

Supplementary Fig. 15 XRD patterns of Co-ZIF-67/Pt and Co-ZIF-67/Pt after heat treatment in flowing H_2 gas.

The characteristic diffraction peaks of Co-ZIF-67/Pt (blue curve) well match those of Co-ZIF-67 simulation, suggesting the structural stability of MOFs after loading Pt nanoparticles. After Co-ZIF-67/Pt is treated in H_2 at 450°C for 30 min, the diffraction peaks of Co-ZIF-67 completely disappear (purple curve) while a new peak attributed to the metallic Co [111] appears, which proves that Co^{2+} is reduced by hydrogen atoms *via* hydrogen spillover.

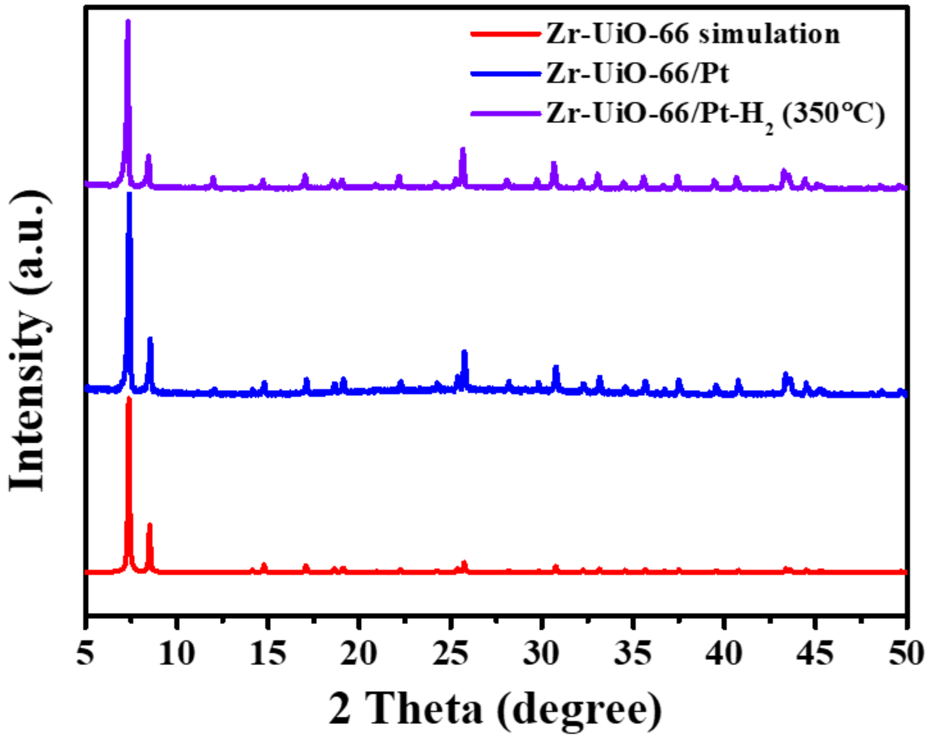

Supplementary Fig. 16 XRD patterns of Zr-UiO-66/Pt and Zr-UiO-66/Pt after heat treatment in flowing H_2 gas.

The characteristic diffraction peaks of Zr-UiO-66/Pt (blue curve) well match those of Zr-UiO-66 simulation, suggesting the structural stability of MOFs after loading Pt nanoparticles. After Zr-UiO-66/Pt is treated in H_2 at 350°C for 30 min, there is no obvious change in each diffraction peak, which proves that Zr-UiO-66 is stable in the spillover state.

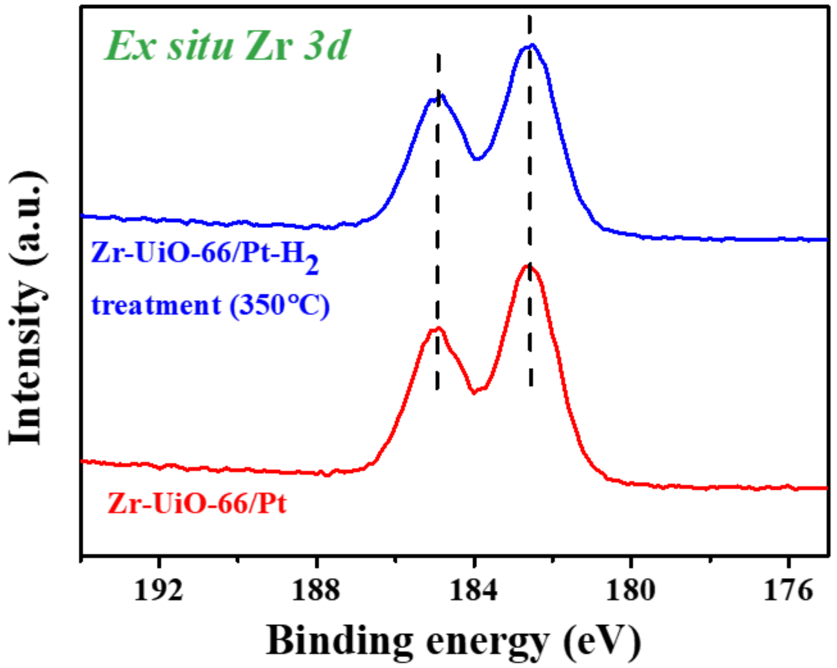

Supplementary Fig. 17 XPS spectra (Zr 3d) of Zr-UiO-66/Pt and Zr-UiO-66/Pt after heat treatment in flowing H_2 gas.

Two characteristic peaks of Zr are observed in the XPS spectrum of Zr-UiO-66/Pt (red curve), where are located at 184.9 eV and 182.6 eV corresponding to Zr^{4+} $3d_{3/2}$ and Zr^{4+} $3d_{5/2}$, respectively. After Zr-UiO-66/Pt is treated in H_2 at 350°C for 30 min, no peak shifts were observed on Zr-UiO-66/Pt. In good agreement with the XRD result (Supplementary Fig. 16), the valence states of Zr elements in Zr-UiO-66 samples remain intact in the presence of H_2 and dissociated hydrogen. This phenomenon is caused by the low reduction potential of Zr-ZIFs, which means that hydrogen (molecule or atom) cannot reduce the tetravalent Zr^{4+}.

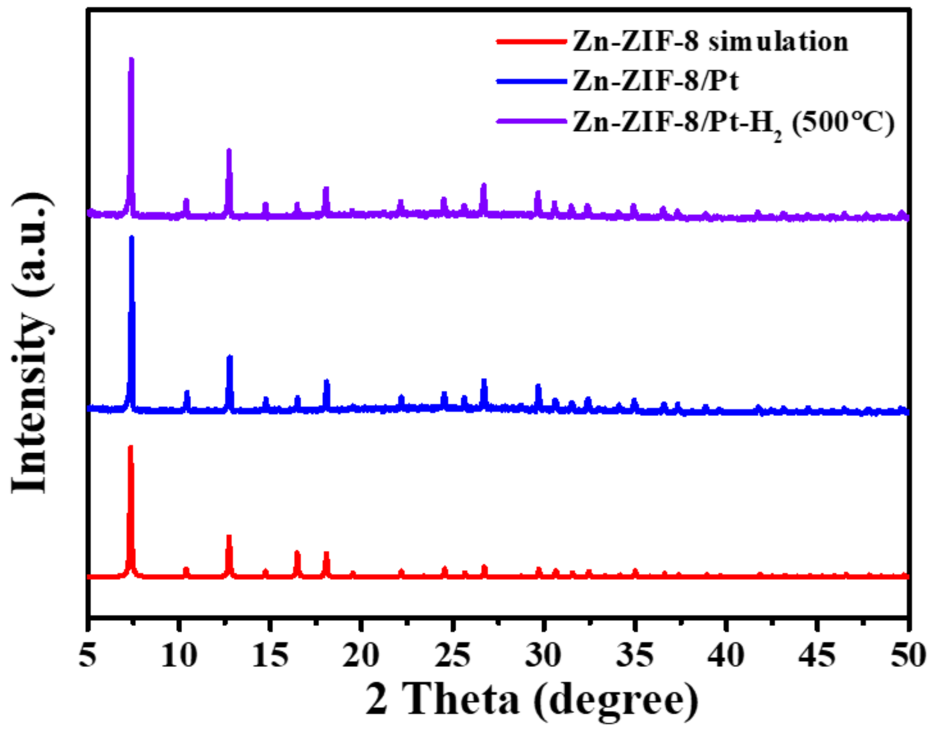

Supplementary Fig. 18 XRD patterns of Zn-ZIF-8/Pt and Zn-ZIF-8/Pt after heat treatment in flowing H_2 gas.

The diffraction peak positions of Zn-ZIF-8/Pt and their relative intensities are well indexed to Zn-ZIF-8 simulation, suggesting the structural stability of MOFs after loading Pt nanoparticles. When Zn-MOFs/Pt treated in H_2 at 500°C for 30 min, there is no obvious change in each diffraction peak, which proves that Zn-MOFs are stable in the spillover state.

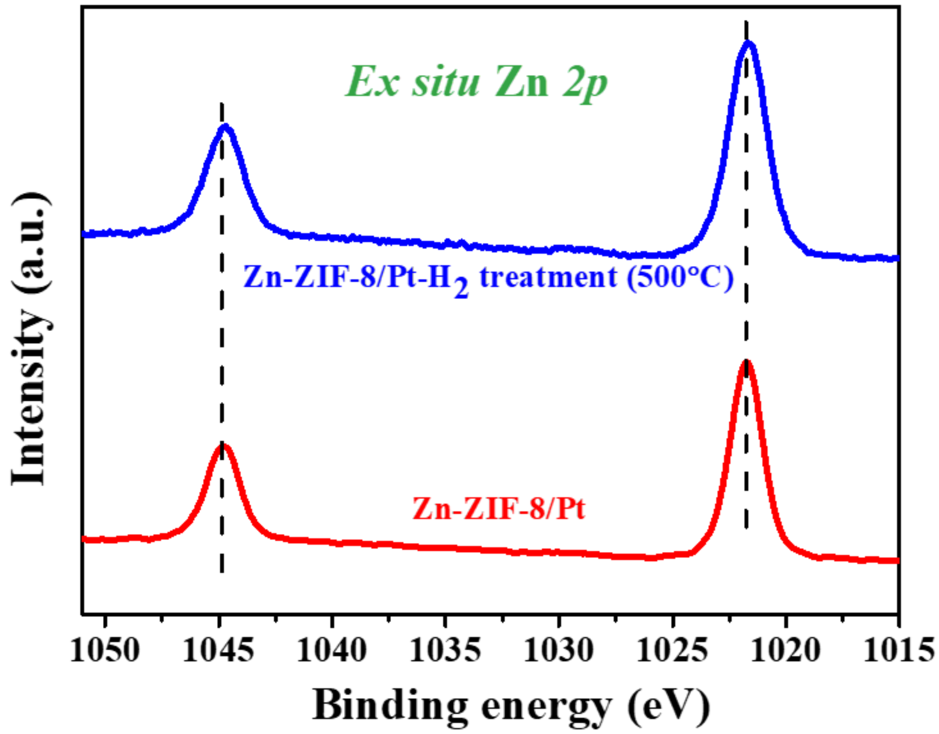

Supplementary Fig. 19 XPS spectra (Zn 2p) of Zn-ZIF-8/Pt and Zn-ZIF-8/Pt after heat treatment in flowing H_2 gas.

Two characteristic peaks of Zn are observed in the XPS spectra of Zn-ZIF-8/Pt, where are located at 1021 eV and 1044 eV corresponding to Zn^{2+} $2p_{3/2}$ and Zn^{2+} $2p_{1/2}$, respectively. After Zn-ZIF-8/Pt is treated in H_2 at 500°C for 30 min, no peak shifts are observed on Zn-ZIF-8/Pt. In good agreement with the XRD result (Supplementary Fig. 18), the valence states of Zn elements in Zn-ZIF-8 samples remain intact in the presence of H_2 and dissociated hydrogen. This phenomenon is caused by the low reduction potential of Zn-ZIFs, which means that hydrogen (molecule or atom) cannot reduce the divalent Zn^{2+}.

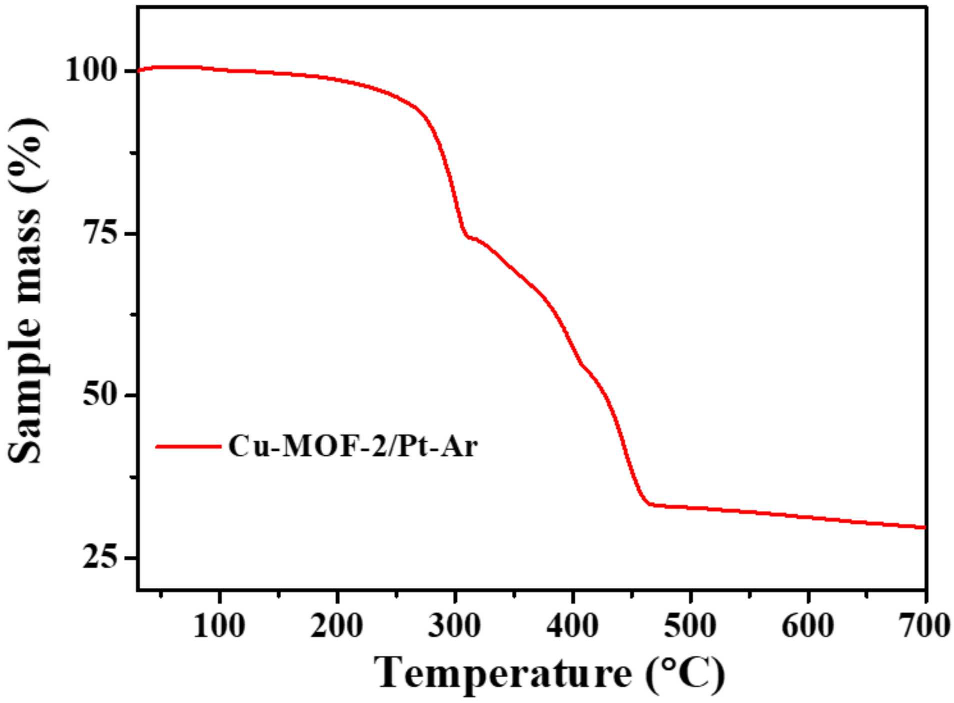

Supplementary Fig. 20 TGA curve of Cu-MOF-2/Pt in Ar.

As shown in Supplementary Fig. 20, the mass loss below 250°C is caused by the removal of coordinated solvent molecule (DMF). Obviously, the thermal decomposition of Cu-MOF-2/Pt starts from 310°C.

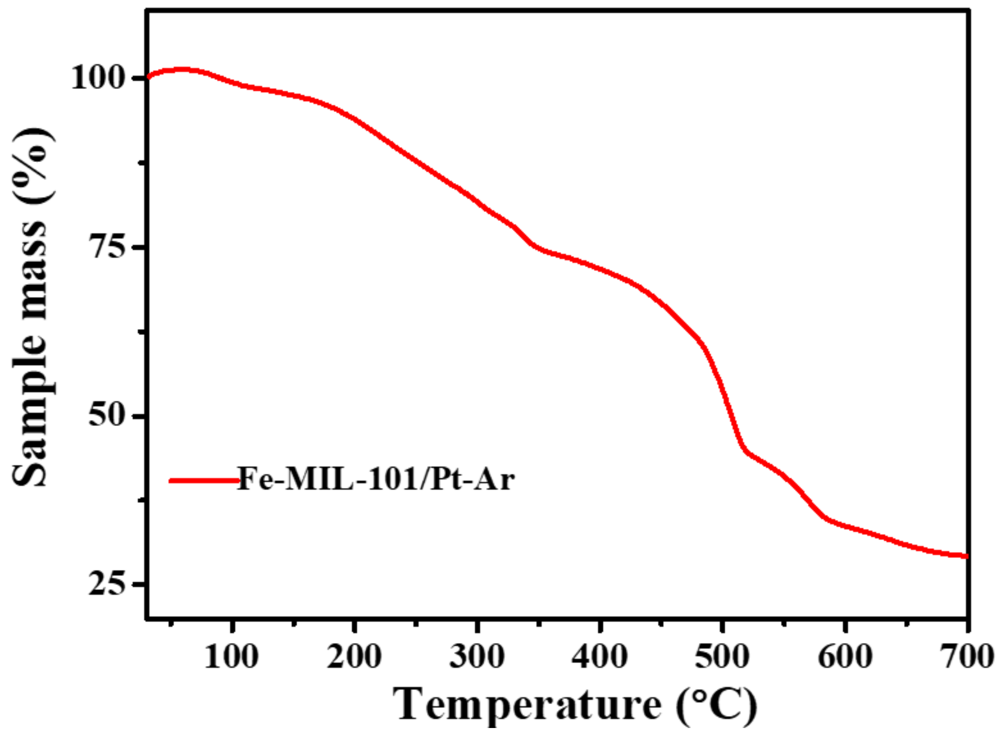

Supplementary Fig. 21 TGA curve of Fe-MIL-101/Pt in Ar.

As shown in Supplementary Fig. 21, the mass loss below 300°C is caused by the removal of guest molecule (DMF). Obviously, the thermal decomposition of Fe-MIL-101/Pt starts from 450°C.

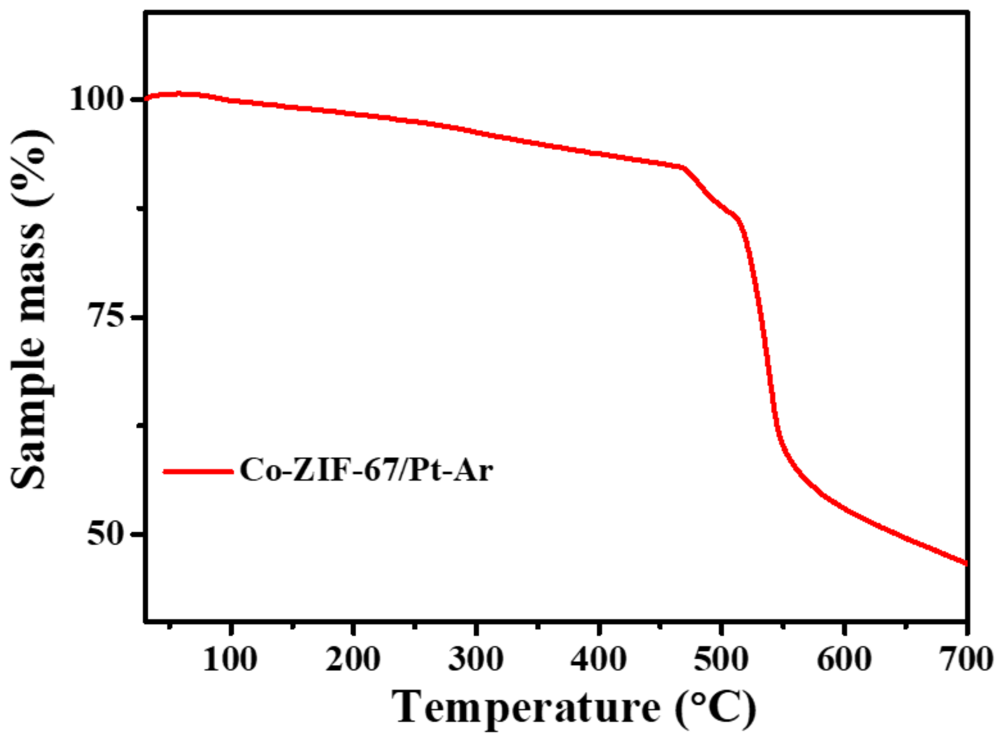

Supplementary Fig. 22 TGA curve of Co-ZIF-67/Pt in Ar.

As shown in Supplementary Fig. 22, Co-ZIF-67/Pt possesses good thermo-stability, and its thermal decomposition starts from 470°C.

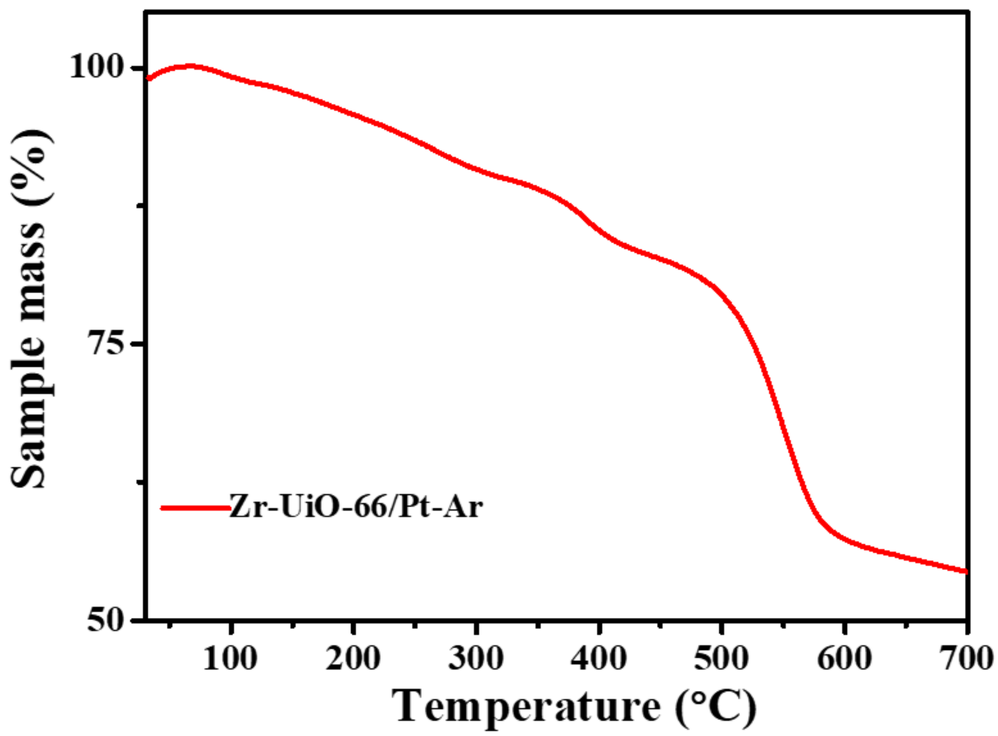

Supplementary Fig. 23 TGA curve of Zr-UiO-66/Pt in Ar.

As shown in Supplementary Fig. 23, the mass loss below 300°C is caused by the removal of guest molecule. Obviously, the thermal decomposition of Zr-UiO-66/Pt starts from 370°C.

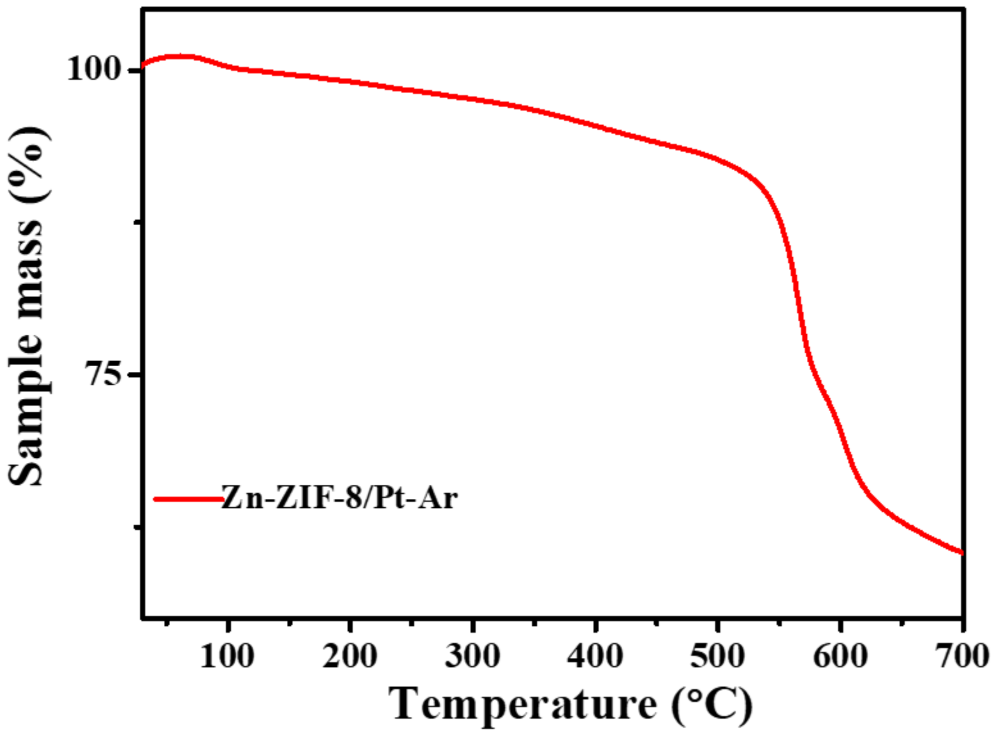

Supplementary Fig. 24 TGA curve of Zn-ZIF-8/Pt in Ar.

As shown in Supplementary Fig. 24, Zn-ZIF-8/Pt possesses good thermo-stability, and its thermal decomposition starts from 530°C.

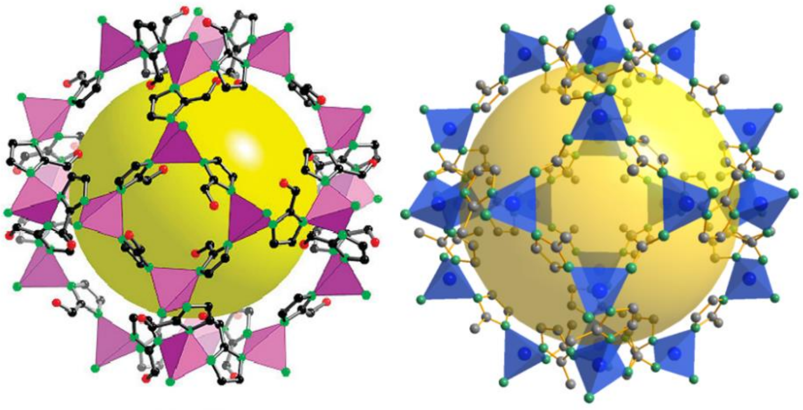

Supplementary Fig. 25. Crystal structure of ZIFs with sodalite (SOD) topologies.[b]

Crystalline zeolitic imidazolate framework, ZIF-8, is prepared from zinc nitrate and 2-methylimidazole (mIm) and possesses the SOD-type topology. And, 2-methylimidazole can be replace by imidazole of other functional groups (e.g., aldehyde and hydroxyl), while their structure remains isomorphic SOD-type frameworks.

[b] Morris, W., Doonan, C. J., Furukawa, H., Banerjee, R. & Yaghi, O. M. Crystals as molecules: Postsynthesis covalent functionalization of zeolitic imidazolate frameworks. *J. Am. Chem. Soc.* **130**, 12626-12627, (2008).

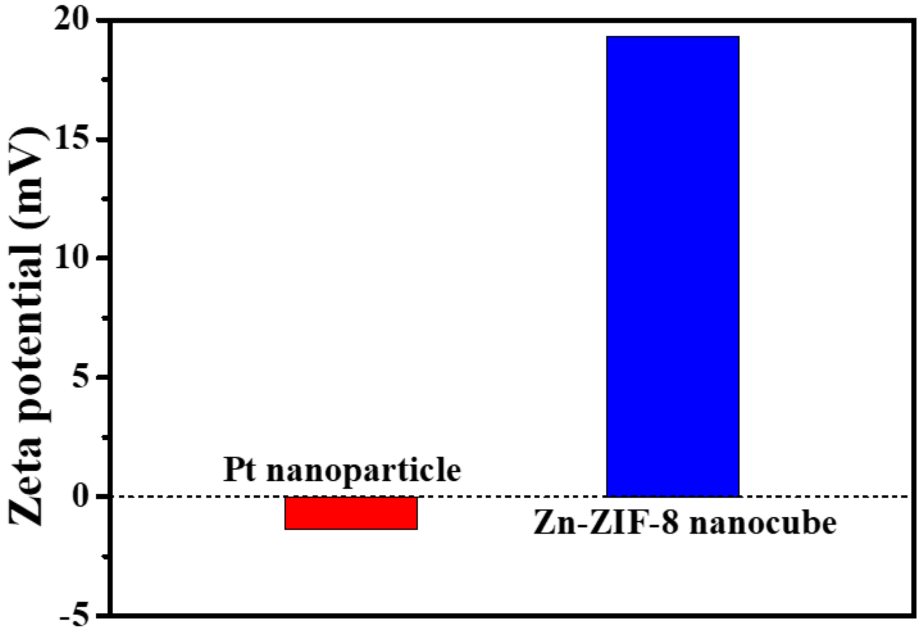

Supplementary Fig. 26 Zeta potential of Pt nanoparticles and Zn-ZIF-8 nanocubes.

Zeta potential results show that the surface charge of Pt nanoparticles is slightly negative (-1.375 mV), while that of Zn-ZIF-8 nanocubes is significantly positive (19.3 mV). The opposite surface charges ensure that Pt nanoparticles are easily adsorbed onto Zn-ZIF-8 nanocubes via electrostatic attraction.

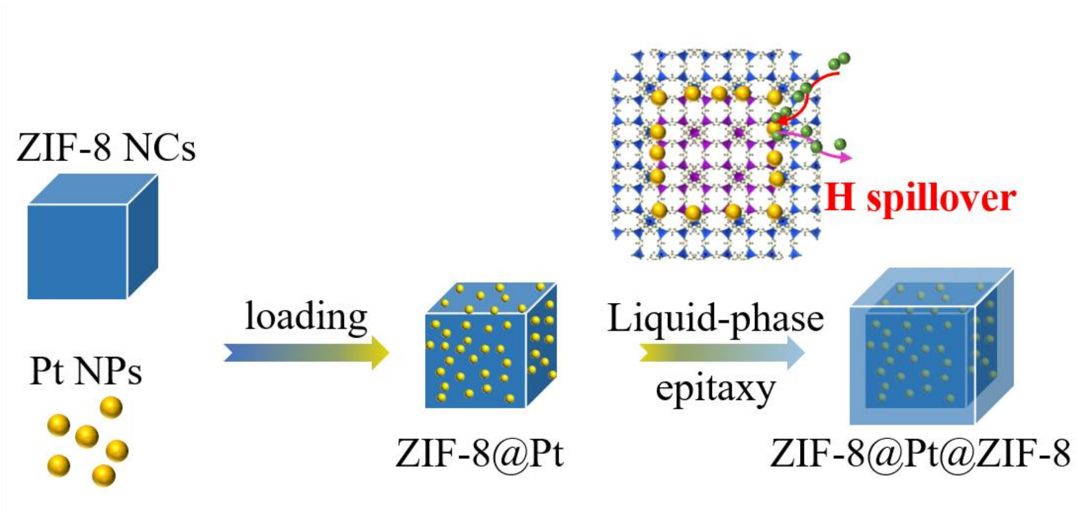

Supplementary Fig. 27 Preparation route of sandwich Zn-ZIF-8@Pt@Zn-ZIF-8.

To ensure the identical diffusion pathway/distance of hydrogen atoms from the Pt nanoparticles to the surface of outer Zn-ZIF-8 shell, it requires as-obtained Zn-ZIF-8@Pt@Zn-ZIF-8 to possess the highly symmetrical and uniform structure. Therefore, Zn-ZIF-8 nanocubes enclosed by six identical [100] surfaces were firstly prepared by using a surfactant-mediated method at room temperature, and then the pre-synthesized Pt nanoparticles were adsorbed on the surface of Zn-ZIF-8 nanocubes via electrostatic adsorption, forming Zn-ZIF-8@Pt. Finally, a Zn-ZIF-8 shell crystallized vertically along the crystal facets of Zn-ZIF-8@Pt via epitaxial growth, giving rise to formation of highly symmetrical and uniform Zn-ZIF-8@Pt@Zn-ZIF-8.

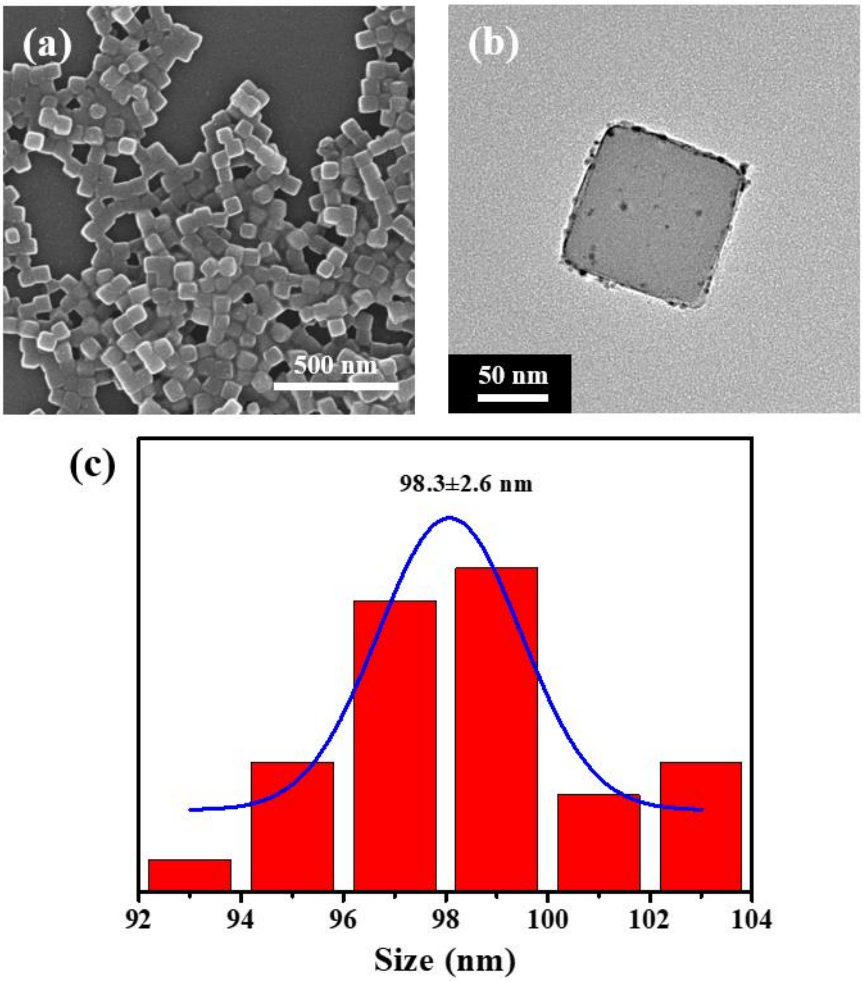

Supplementary Fig. 28 (a) SEM image and (b) TEM image of Zn-ZIF-8@Pt. (c) Particle size distribution of Zn-ZIF-8@Pt.

The Zn-ZIF-8 nanocube cores were obtained by using a surfactant-mediated method at room temperature, and then the pre-synthesized Pt nanoparticles were adsorbed on the surface of Zn-ZIF-8 nanocube cores via electrostatic adsorption, forming Zn-ZIF-8@Pt. Notably, as-produced Zn-ZIF-8@Pt exhibits the cubic shape with high uniformity.

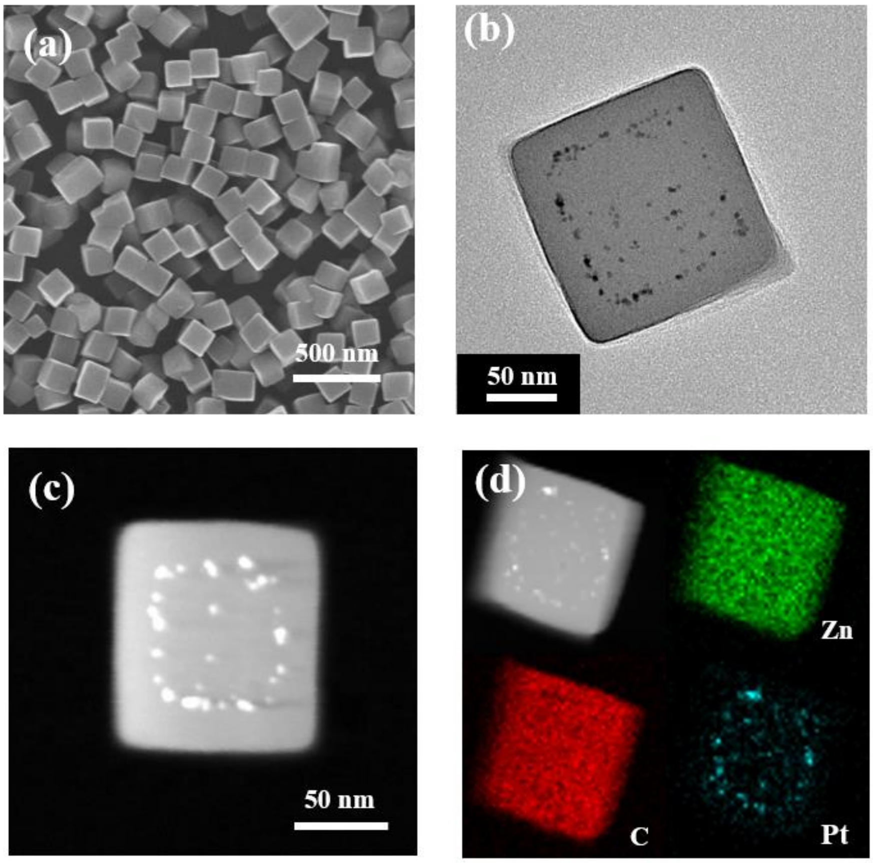

Supplementary Fig. 29 (a) SEM image, (b) TEM image, (c) STEM and (d) EDX element mapping images of Zn-ZIF-8@Pt@Zn-ZIF-8.

According to SEM and TEM observations, all the produced Zn-ZIF-8@Pt and Zn-ZIF-8@Pt@Zn-ZIF-8 are of the cubic shape with high uniformity, clearly confirming that the shells are grown in an epitaxial manner. Energy dispersive X-ray (EDX) elemental mapping result further shows the well-defined distribution of Pt nanoparticles in the sandwich structure.

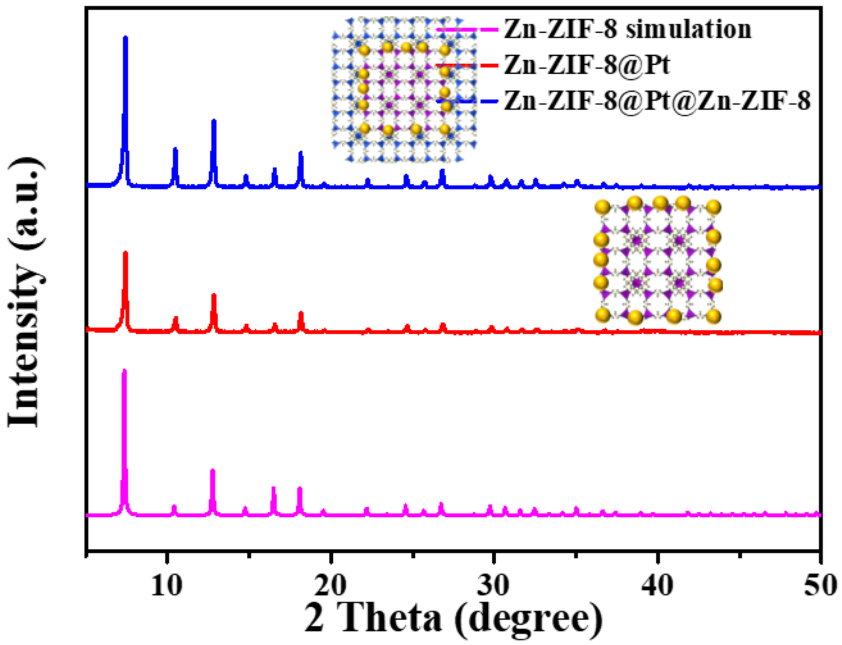

Supplementary Fig. 30 XRD patterns of Zn-ZIF-8@Pt and Zn-ZIF-8@Pt@Zn-ZIF-8.

The crystal structure of Zn-ZIF-8@Pt is identical to that of pure Zn-ZIF-8 (simulation), proving that the adsorption process of Pt nanoparticles does not damage the original structure of Zn-ZIF-8. After the epitaxial growth of outer shell, the diffraction peak position remains unchanged but the peak intensity increases obviously, indicating the successful preparation of Zn-ZIF-8@Pt@Zn-ZIF-8.

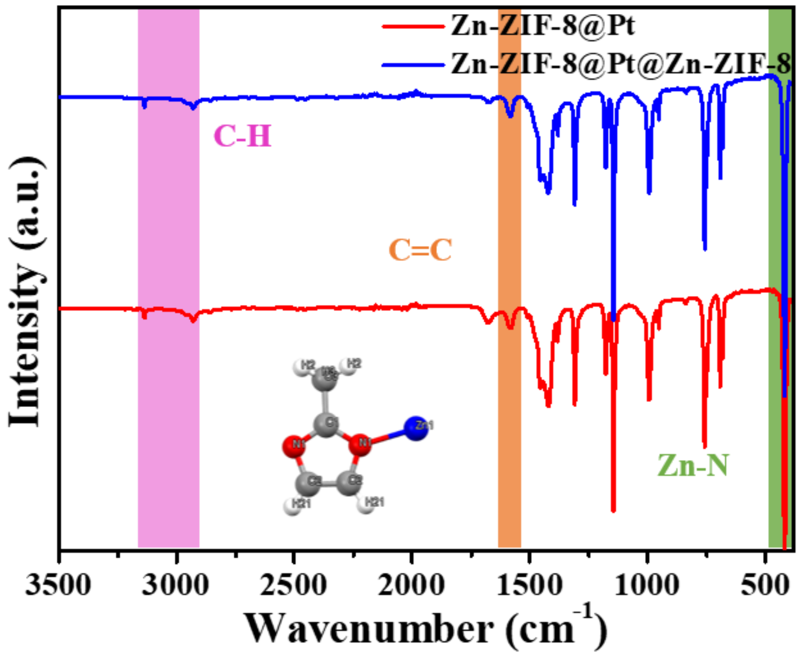

Supplementary Fig. 31 FT-IR spectra of Zn-ZIF-8@Pt and Zn-ZIF-8@Pt@Zn-ZIF-8.

The isostructural Zn-ZIF-8@Pt and Zn-ZIF-8@Pt@Zn-ZIF-8 share the same infrared absorption features (Supplementary Fig. 31). Clearly, the C-H stretching vibration absorption (2929 cm^{-1}), the C=C stretching vibration absorption (1584 cm^{-1}) and the Zn-N stretching vibration absorption (414 cm^{-1}) are observed in the Fourier transform infrared (FT-IR) spectra, which proves the successful synthesis of Zn-ZIF-8.

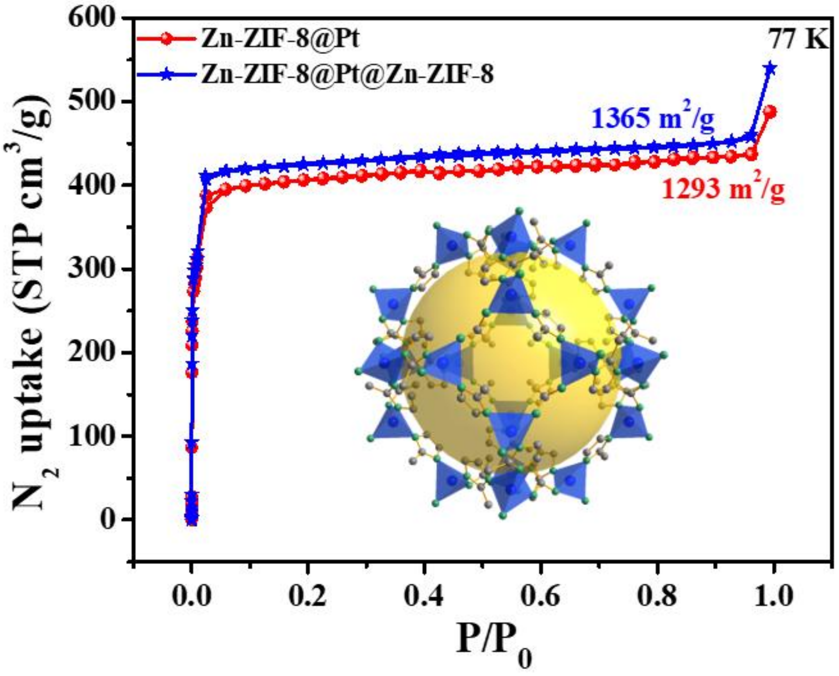

Supplementary Fig. 32 N_2 adsorption/desorption isotherms of Zn-ZIF-8@Pt and Zn-ZIF-8@Pt@Zn-ZIF-8.

The N_2 adsorption-desorption isotherms of Zn-ZIF-8@Pt and Zn-ZIF-8@Pt@Zn-ZIF-8 are identified as type I, exhibiting the features typical of microporous materials. While the latter shows a slight increase in microporous adsorption and specific surface area (S_{BET} rises from 1293 m^2/g to 1365 m^2/g) because of the epitaxial growth of outer shell.

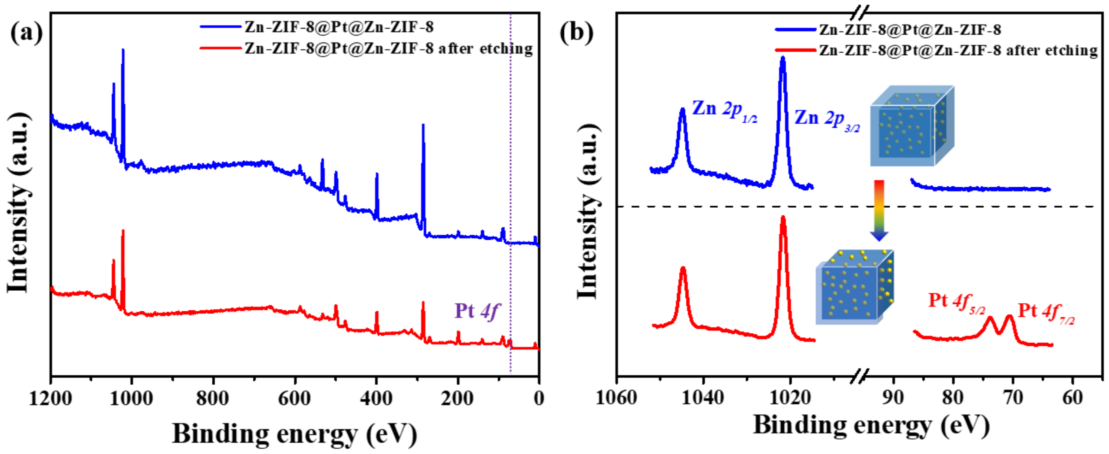

Supplementary Fig. 33 (a) XPS survey spectra of Zn-ZIF-8@Pt@Zn-ZIF-8 before and after plasma etching. (b) Zn *2p* and Pt *4f* high-resolution spectra of Zn-ZIF-8@Pt@Zn-ZIF-8 before and after plasma etching.

XPS depth profiles obtained via the plasma-etching technique were employed to determine the core-shell structure of Zn-ZIF-8@Pt@Zn-ZIF-8. The original XPS spectrum of Zn-ZIF-8@Pt@Zn-ZIF-8 displays only C, N, O and Zn elements while additional Pt element is distinguished after Ar plasma etching, suggesting that Pt nanoparticles are completely encapsulated by the Zn-ZIF-8 shell. The Pt $4f_{5/2}$ and Pt $4f_{7/2}$ signals located at 73.9 eV/70.7 eV suggest that the surface of sandwich Pt nanoparticles is zero charged.

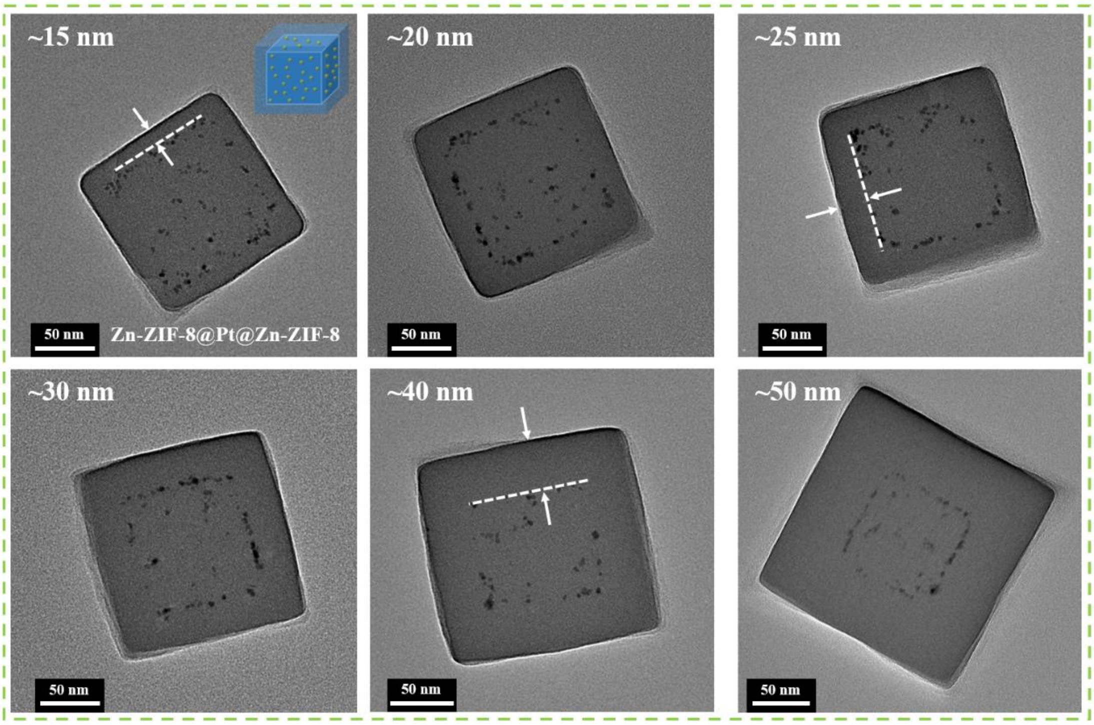

Supplementary Fig. 34 Tunable thickness of Zn-ZIF-8 shell (A ruler for hydrogen spillover).

The Zn-ZIF-8 shell is obtained by liquid-epitaxial growth. So, the thickness of Zn-ZIF-8 shell can be adjusted by changing the concentration of precursors (metal ions and organic ligands), which is precisely controlled from 15 nm to 50 nm (15, 20, 25, 30, 40 and 50 nm).

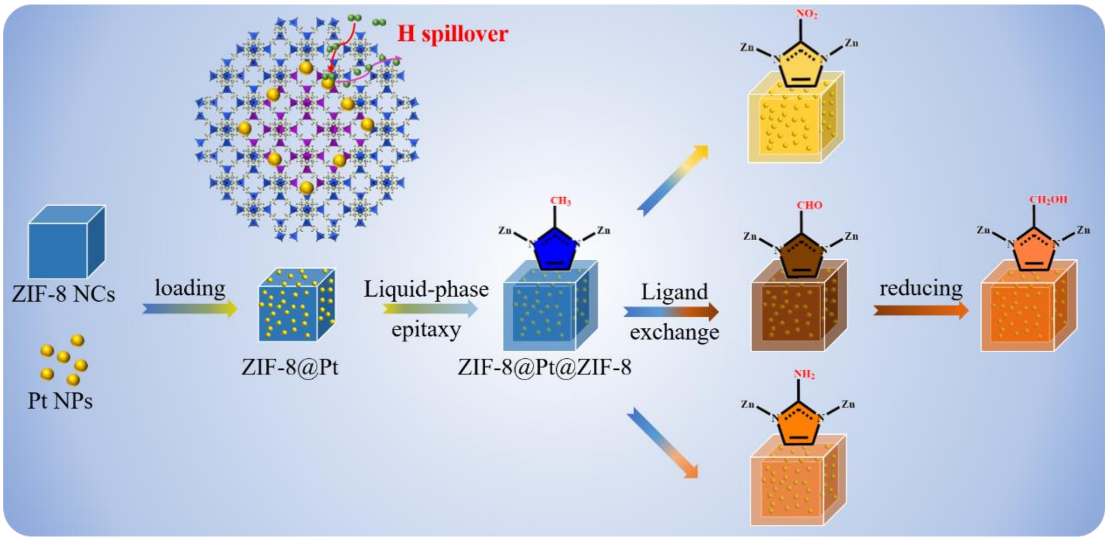

Supplementary Fig. 35 Scheme of preparation of Zn-ZIFs@Pt@Zn-ZIFs homologues by ligand exchange strategy.

In order to obtain Zn-ZIFs@Pt@Zn-ZIFs homologs with different functional groups (CHO, OH, NO_2 and NH_2), solvent-assisted ligand exchange/reduction strategy is adopted to change the original Zn-ZIF-8@Pt@Zn-ZIF-8.

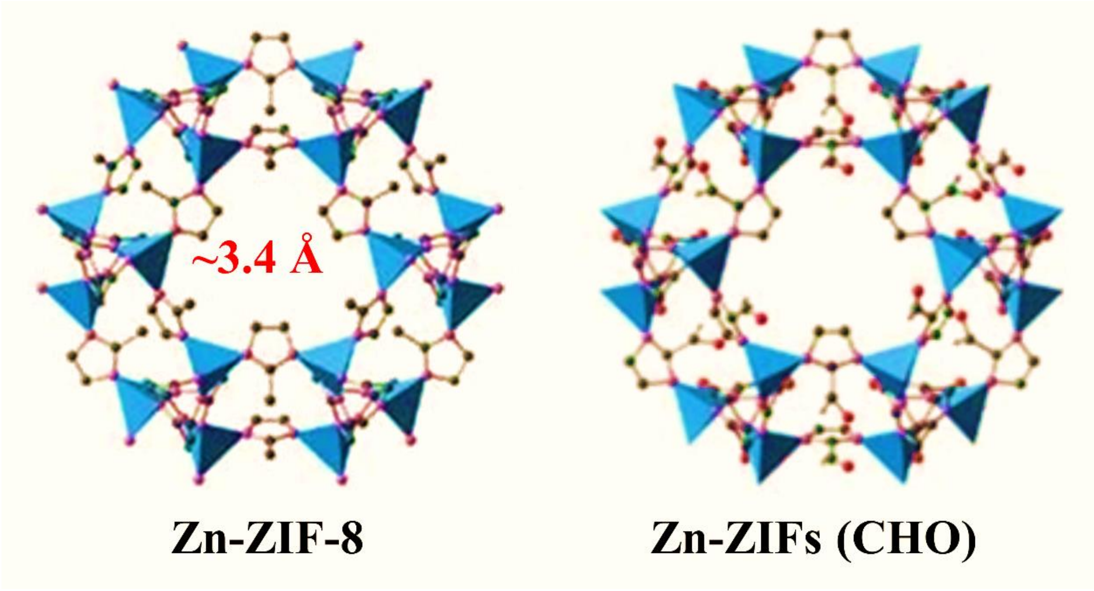

Supplementary Fig. 36 Scheme of Zn-ZIF-8 and Zn-ZIFs (CHO) aperture window.

As demonstrated in Supplementary Fig. 36, Zn-ZIF-8 has very small aperture window (~0.34 nm) and selectively allow the diffusion of H_2 (0.29 nm) over large cyclooctene molecule (~0.6 nm) (Supplementary Fig. 37). In addition, the ligand exchange/reduction process leads to the minimal change in its aperture window. Zn-ZIFs (CHO) CCDC number: 693596.

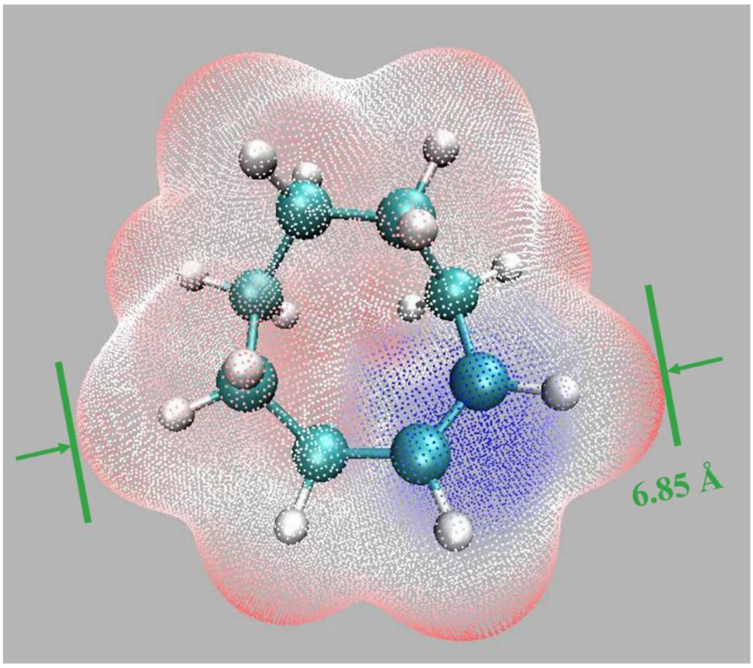

Supplementary Fig. 37 Molecular kinetic diameter of cyclooctene.

The kinetic diameter of cyclooctene is about 6.85 Å by theoretical simulation[c-e], which reveals that cyclooctene could not enter the Zn-ZIF-8 pore.

[c] Lu, T. & Chen, F. Multiwfn: A multifunctional wavefunction analyzer. *J. Comput. Chem.* **33**, 580-592, (2012).
[d] Lu, T. & Chen, F. Quantitative analysis of molecular surface based on improved Marching Tetrahedra algorithm. *Journal of Molecular Graphics & Modelling* **38**, 314-323, (2012).
[e] Humphrey, W., Dalke, A. & Schulten, K. VMD: Visual molecular dynamics. *Journal of Molecular Graphics & Modelling* **14**, 33-38, (1996).

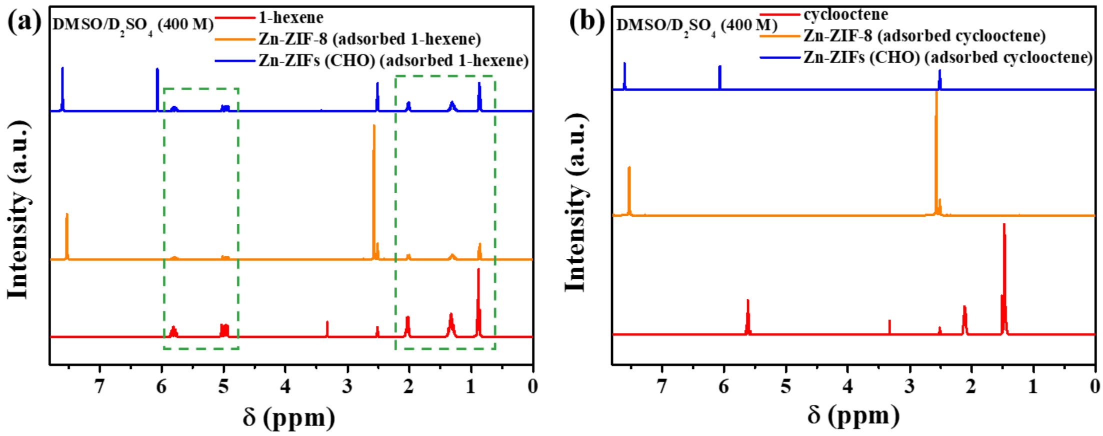

Supplementary Fig. 38 (a) ^{1}H NMR spectra of 1-hexene, Zn-ZIF-8 after adsorption of 1-hexene and Zn-ZIFs (CHO) after adsorption of 1-hexene. (b) ^{1}H NMR spectra of cyclooctene, Zn-ZIF-8 after adsorption of cyclooctene and Zn-ZIFs (CHO) after adsorption of cyclooctene.

To prove that cyclooctene cannot pass through the Zn-ZIF-8 pore, comparative adsorption experiments were performed. First, Zn-ZIF-8 and Zn-ZIFs (CHO) were thoroughly dried and activated in a vacuum at 120°C, and then soaked in 1-hexene solution. After continuous stirring overnight, the samples were centrifuged with alcohol twice and dried naturally. The dried powders were dispersed in deuterium DMSO/D_2SO_4 and their ^{1}H NMR spectra were determined. As shown in Supplementary Fig. 38a, there is still a certain amount of 1-hexene, indicating the adsorption capability of Zn-ZIFs for small molecule 1-hexene. In sharp contrast, for larger cyclooctene, no cyclooctene molecules are detected in Zn-ZIFs after the same treatment (Supplementary Fig. 38b), proving that cyclooctene could not enter the pore of Zn-ZIFs.

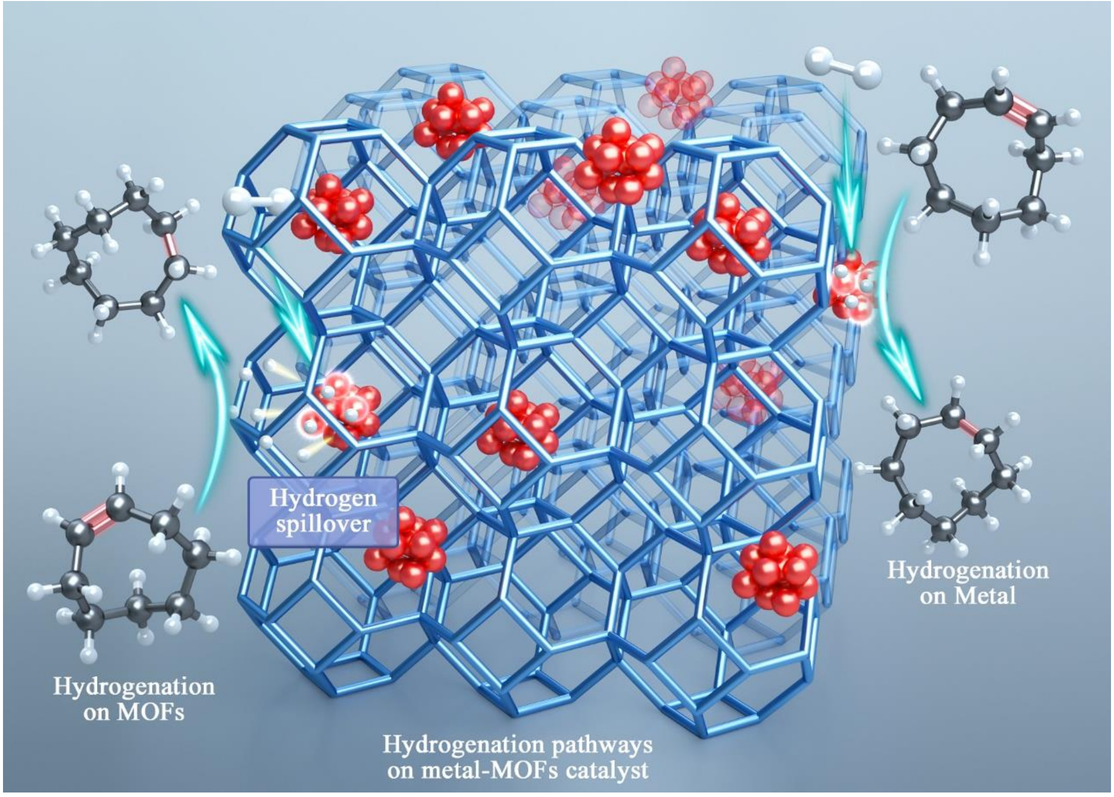

Supplementary Fig. 39 Hydrogenation pathways of cyclooctene on MOFs/metal NPs catalyst.

In the conventional MOFs supported metal nanoparticles (MOFs/metal NPs) catalysts, metal nanoparticles are randomly distributed in porous MOFs structure or near MOFs surface. So, both organic substrates and H_2 can diffuse onto the metal nanoparticle surface in the course of the catalytic reaction. This allows the reactions to occur not only on the MOFs surface but also on the metal nanoparticle surface, leading to difficulty in decoupling the contributions from both pathways.

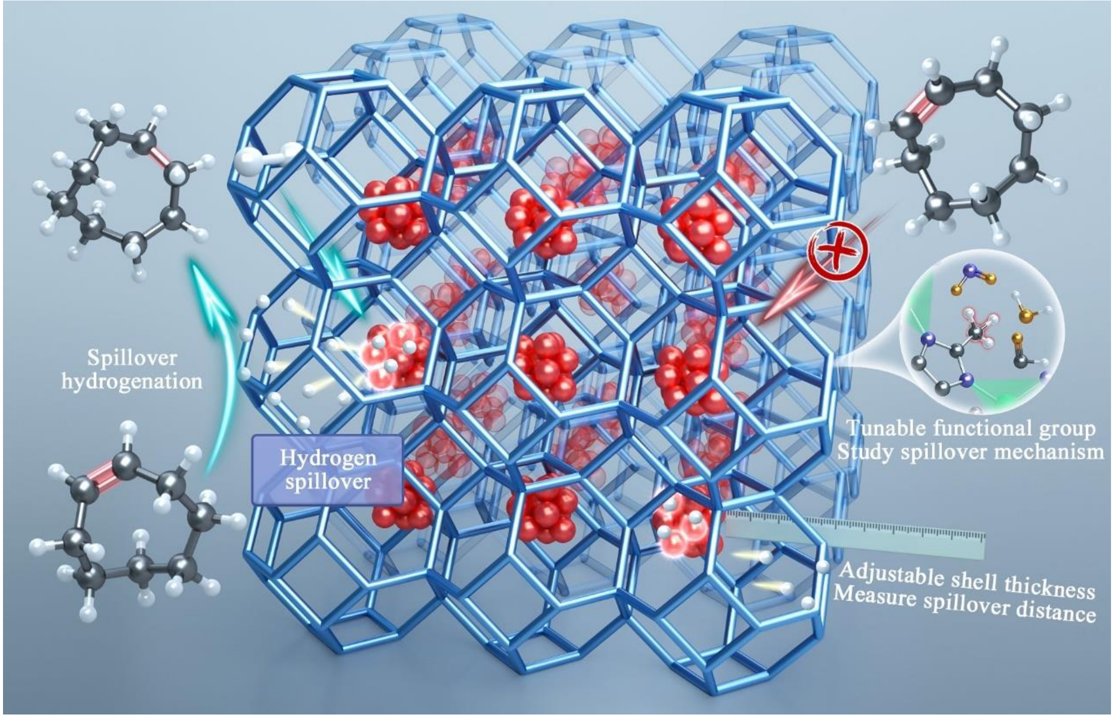

Supplementary Fig. 40 Hydrogenation pathway of cyclooctene on MOFs@Pt@MOFs.

Here, we design MOFs@Pt@MOFs catalysts, where metal catalysts are fully located inside with a uniform and tunable MOFs shell (thickness and functional group). Furthermore, we intentionally select the organic molecules of size larger than aperture of MOFs as the reactants. In this case, only small H_2 can access Pt nanoparticles but larger organic reactant cannot. So, all the catalytic activities are directly attributed to the reaction between spillover hydrogen and organic molecules on the external surface of MOFs matrix. The tunable functional group is unique to study the spillover mechanism and the controlled shell thickness is key to accurately measure the spillover distance.

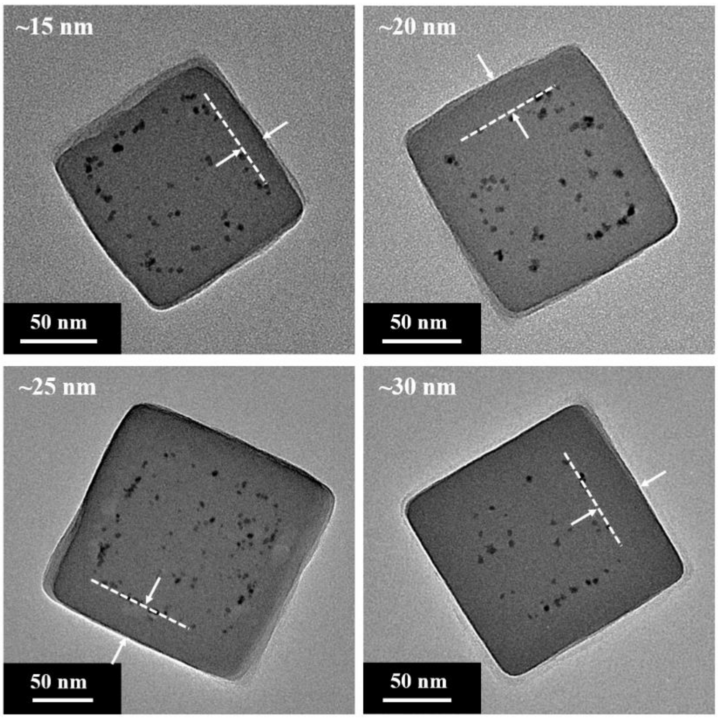

Supplementary Fig. 41 Different shell thickness Zn-ZIFs@Pt@Zn-ZIFs (CHO).

The Zn-ZIFs@Pt@Zn-ZIFs (CHO) shell was obtained by solvent-assisted ligand exchange strategy. So, the thickness of Zn-ZIFs shell is highly dependent on that of the pristine Zn-ZIF-8@Pt@Zn-ZIF-8.

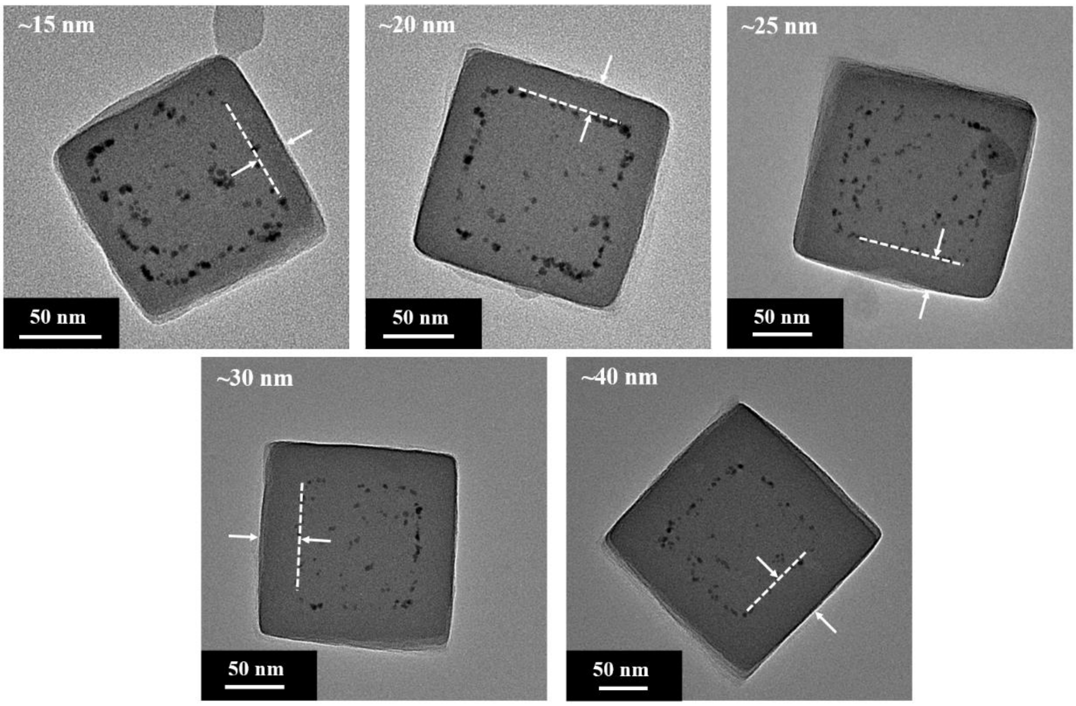

Supplementary Fig. 42 Different shell thickness Zn-ZIFs@Pt@Zn-ZIFs (OH).

The Zn-ZIFs@Pt@Zn-ZIFs (OH) shell was obtained by solvent-assisted ligand reduction strategy. So, the thickness of Zn-ZIFs shell is highly dependent on that of the pristine Zn-ZIFs@Pt@Zn-ZIFs (CHO).

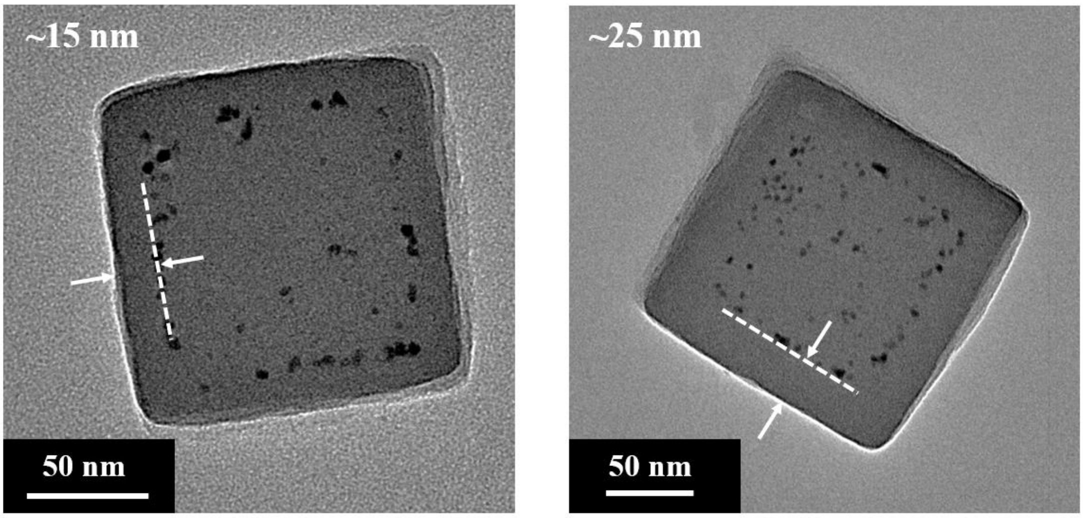

Supplementary Fig. 43 Different shell thickness Zn-ZIFs@Pt@Zn-ZIFs (NO_2).

The Zn-ZIFs@Pt@Zn-ZIFs (NO_2) shell was obtained by solvent-assisted ligand exchange strategy. So, the thickness of Zn-ZIFs shell is highly dependent on that of the pristine Zn-ZIF-8@Pt@Zn-ZIF-8.

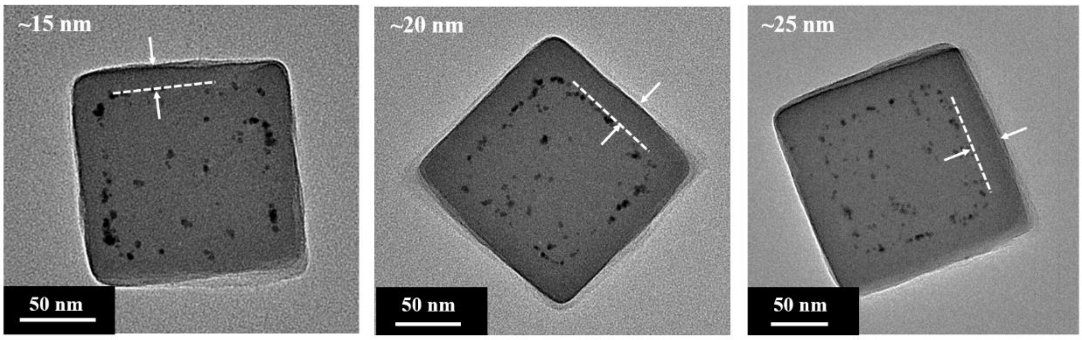

Supplementary Fig. 44 Different shell thickness Zn-ZIFs@Pt@Zn-ZIFs (NH_2).

The Zn-ZIFs@Pt@Zn-ZIFs (NH_2) shell was obtained by solvent-assisted ligand exchange strategy. So, the thickness of Zn-ZIFs shell is highly dependent on that of the pristine Zn-ZIF-8@Pt@Zn-ZIF-8.

Table S1 Hydrogenation conversion ratios of cyclooctene by ZIFs@Pt@ZIFs homologs and contrast catalyst.

Cata.	Reductant	Temp.	Time (min)	Conv. (%)
Pt/SiO_2	2 MPa H_2	80°C	120	99.9
Zn-ZIF-8	2 MPa H_2	80°C	120	0
Zn-ZIF-8@Pt@Zn-ZIF-8 (dry)	2 MPa H_2	80°C	120	0
Zn-ZIF-8@Pt@Zn-ZIF-8 (dry)	2 MPa H_2	100°C	960	~0
Zn-ZIF-8@Pt@Zn-ZIF-8 (H_2O)	2 MPa H_2	80°C	120	96.1
				91.3
				93.4
Zn-ZIFs@Pt@Zn-ZIFs (CHO)	2 MPa H_2	80°C	120	71.3
				68.0
				75.4
Zn-ZIFs@Pt@Zn-ZIFs (OH)	2 MPa H_2	80°C	120	86.1
				82
				88.8
Zn-ZIFs@Pt@Zn-ZIFs (NO_2)	2 MPa H_2	80°C	120	~3.8
Zn-ZIFs@Pt@Zn-ZIFs (NH_2)	2 MPa H_2	80°C	120	41.9
				40.1
				36.8

No solvent

Shell thickness: 15 nm

Reactant: 0.5 mL cyclooctene

Catalyst mass: containing ~0.004 mmol Pt

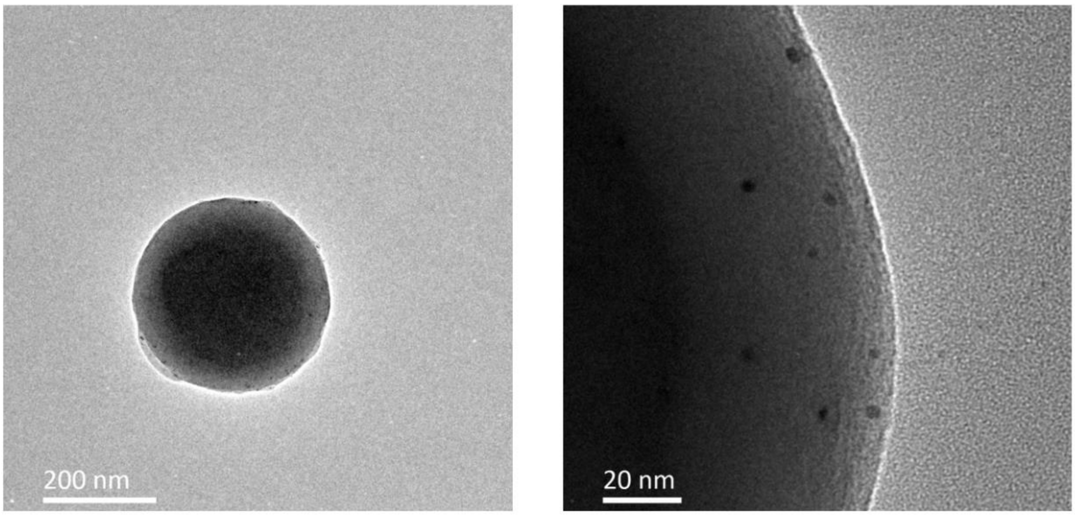

Supplementary Fig. 45 TEM images of SiO_2/Pt.

TEM images show that the diameter of SiO_2 microspheres is about 300 nm, and Pt nanoparticles are uniformly distributed on their surface.

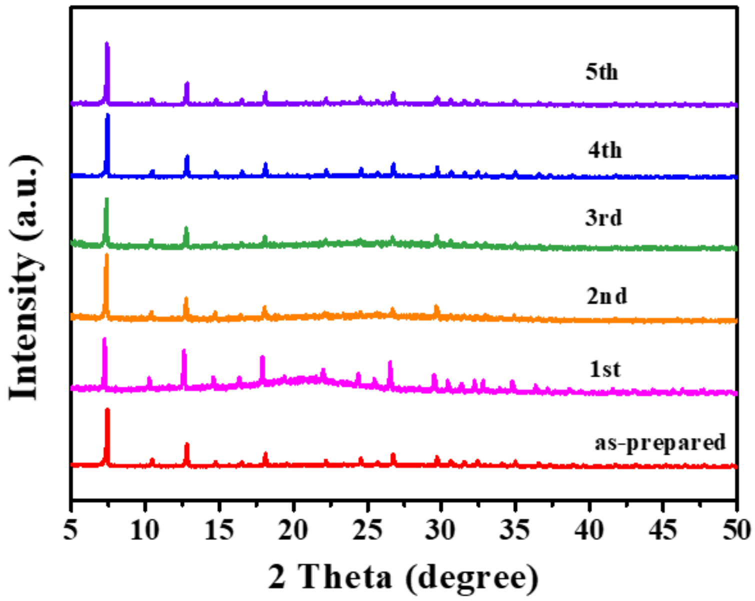

Supplementary Fig. 46 XRD patterns of the catalytic recovery Zn-ZIF-8@Pt@Zn-ZIF-8.

After several catalytic cycles, the X-ray diffraction peaks of Zn-ZIF-8@Pt@Zn-ZIF-8 do not change significantly, which proves its structural stability in the hydrogenation reaction.

Table S2 Catalytic stability of Zn-ZIFs@Pt@Zn-ZIFs homologs toward cyclooctene hydrogenation.

	**(H_2O)**	**(CHO)**	**(OH)**	**(NH_2)**
1	96.1	71.3	86.1	40.1
	91.3	68.0	82	41.9
	93.4	75.4	88.8	36.8
2	15.8	69	81.9	33.1
	15.5	64.6	88.7	36.7
	8.7	62.7	86.7	42.4
3	3.5	63.6	84.8	38.4
	6.1	62.8	81.9	41.7
	2.9	67.5	85.7	37.0
4	0	65.5	67.1	40.8
	0	63.8	85.5	36.2
	0	64.0	87.3	32.9
5	0	57.8	79.4	35.2
	0	65.5	70	39.6
	0	69	88	35.1

No solvent

Reductant: 2 MPa H_2

Shell thickness: 15 nm

Reaction time: 120 min

Reactant: 0.5 mL cyclooctene

Catalyst: Zn-ZIFs@Pt@Zn-ZIFs homologs

Recovery of catalyst: centrifugation (ethanol) and natural drying

Table S3 Conversion ratio of cyclooctene by Zn-ZIF-8@Pt@Zn-ZIF-8 at different shell thickness and temperature.

Temp. (℃) \ Thickness	15 nm	20 nm	25 nm	30 nm	40 nm	50 nm
40	33.8	18.3	2.8	0	-	-
	34.8	17.0	2.6	0	-	-
	43.1	20.8	1.4	0	-	-
60	48.6	32.5	17.3	7.2	0	-
	46.9	34.8	20.0	4.9	0	-
	50.4	33.2	22.2	7.1	0	-
80	79.1	63.5	50.6	42.0	25.2	4.5
	74.1	61.7	53.3	40.9	17.8	2.9
	75.5	67.9	56.6	38.9	21.4	3.2

No solvent

Reductant: 2 MPa H_2

Time: 360 min (40℃), 90 min (60℃), 80 min (80℃)

Reactant: 0.5 mL cyclooctene

Catalyst: Zn-ZIF-8@Pt@Zn-ZIF-8 (H_2O)

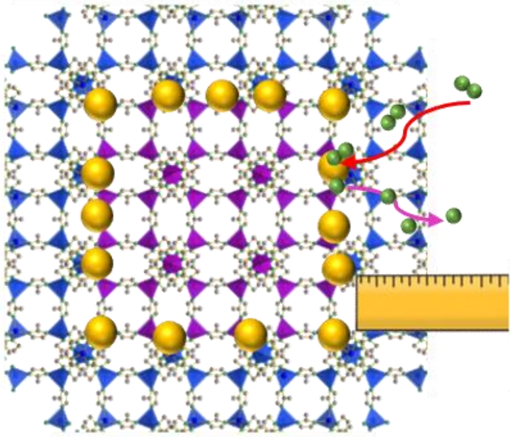

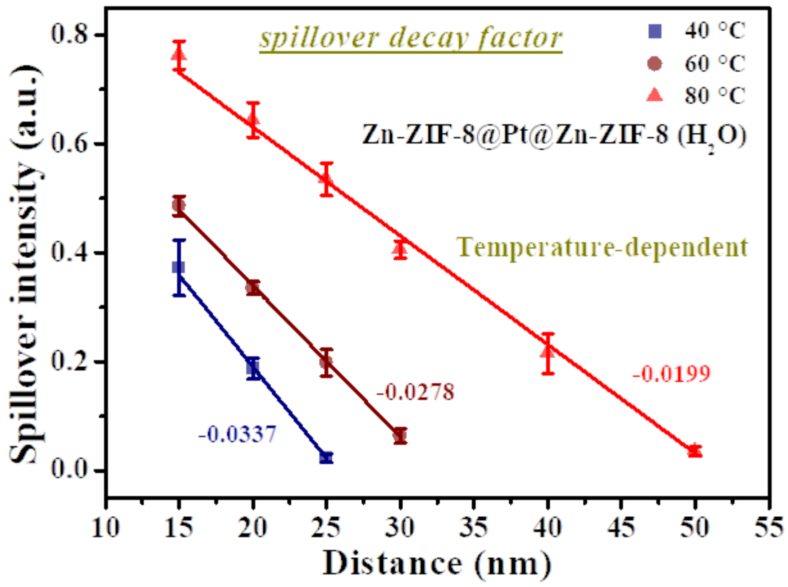

Supplementary Fig. 47 Linear relationship between spillover intensity and spillover distance at different temperatures for Zn-ZIF-8@Pt@Zn-ZIF-8 (H_2O) catalyst. Reaction time: 80 min.

For instance, in the Zn-ZIF-8@Pt@Zn-ZIF-8 (H_2O) system, the spillover intensity decreases with the increase of shell thickness under the same reaction time and temperature. This phenomenon can be represented by a formula: $y = ax + b$ (where y refers to spillover intensity, x refers to the shell thickness and a refers to spillover decay factor). Note that the absolute value of spillover decay factor decreases with the increase of temperature.

Table S4 Conversion ratio of cyclooctene by Zn-ZIFs@Pt@Zn-ZIFs homologs at different shell thickness.

Cata. \ Thickness	15 nm	20 nm	25 nm	30 nm	40 nm	50 nm
(H_2O)	79.1	63.5	50.6	42.0	25.2	4.5
	74.1	61.7	53.3	40.9	17.8	2.9
	75.5	67.9	56.6	38.9	21.4	3.2
(CHO)	55.2	37.8	24.9	14.9	6.0	0
	52.8	41.4	31.8	9.5	1.1	0
	57.9	44.0	27.4	11.8	3.2	0
(OH)	70.9	54.9	43.3	33.1	12.8	0
	67.0	58.3	48.7	33.2	10.7	0
	65.1	59.8	45.6	37.2	14.0	0
(NH_2)	30.8	3	0	-	-	-
	28.6	4.5	0	-	-	-
	24.3	4.4	0	-	-	-

No solvent

Reductant: 2 MPa H_2

Reaction time: 80 min

Reaction temperature: 80°C

Reactant: 0.5 mL cyclooctene

Catalyst: Zn-ZIFs@Pt@Zn-ZIFs homologs

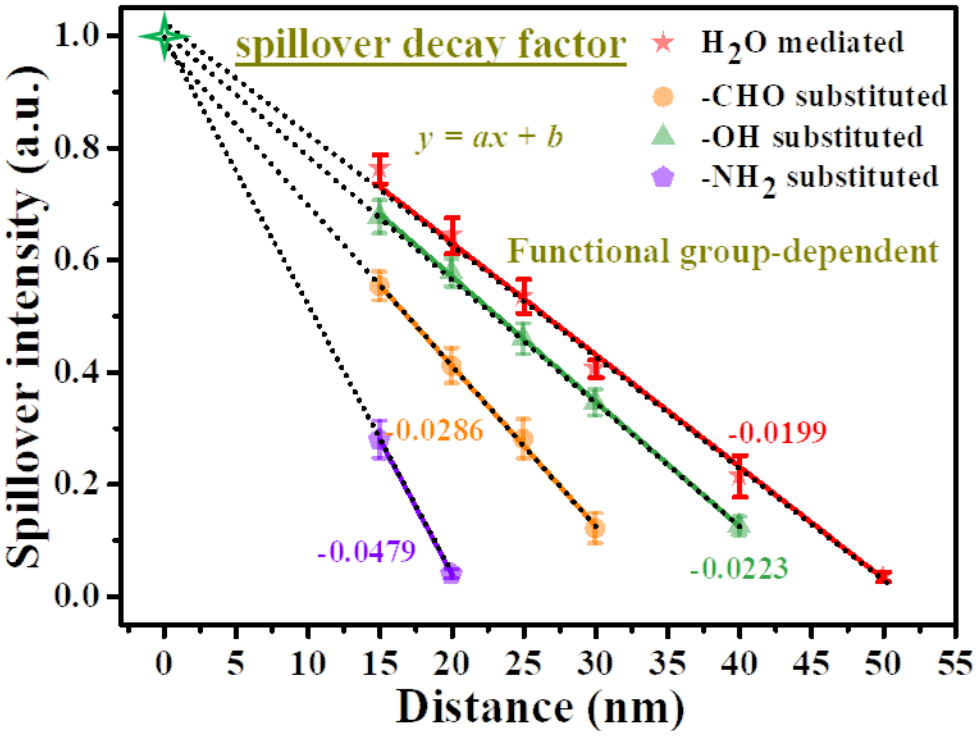

Supplementary Fig. 48 Linear relationship between spillover intensity and spillover distance of Zn-ZIFs@Pt@Zn-ZIFs homologs (80-minute reaction).

At the specific temperature and time (80°C and 80 min), the spillover intensity well fits an equation: $y = ax + b$, where y refers to the spillover intensity, a represents the spillover decay factor, x stands for the shell thickness, and b is the maximum spillover intensity close to 1. The 80-minute reaction time was selected because the conversion ratio of zero-shell samples just approached 100% at that time (longer reaction time eventually gave rise to 100% conversion ratio for all the samples, which could not uncover the influence of varied functional groups and shell thicknesses on hydrogen spillover behavior).

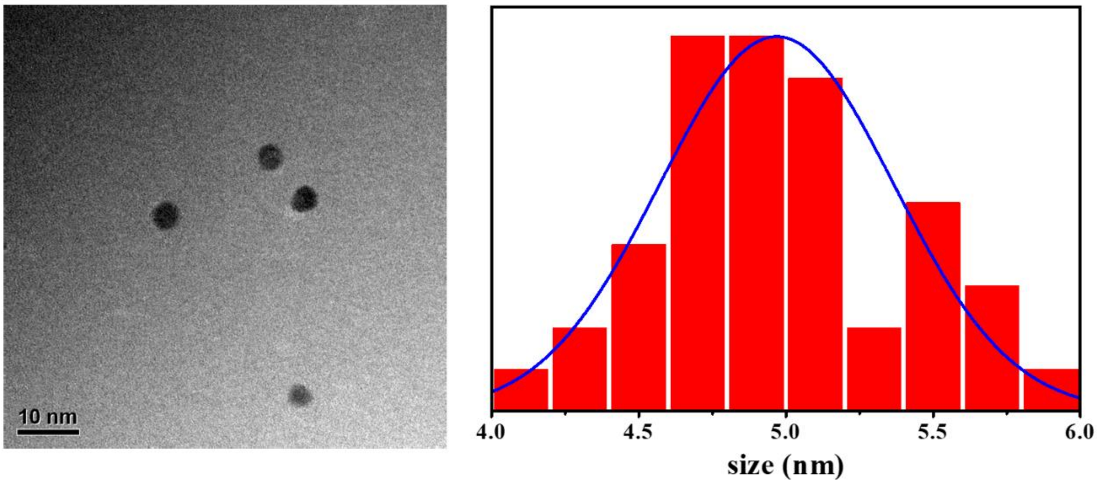

Supplementary Fig. 49 TEM image of Pt NPs and their size distribution.

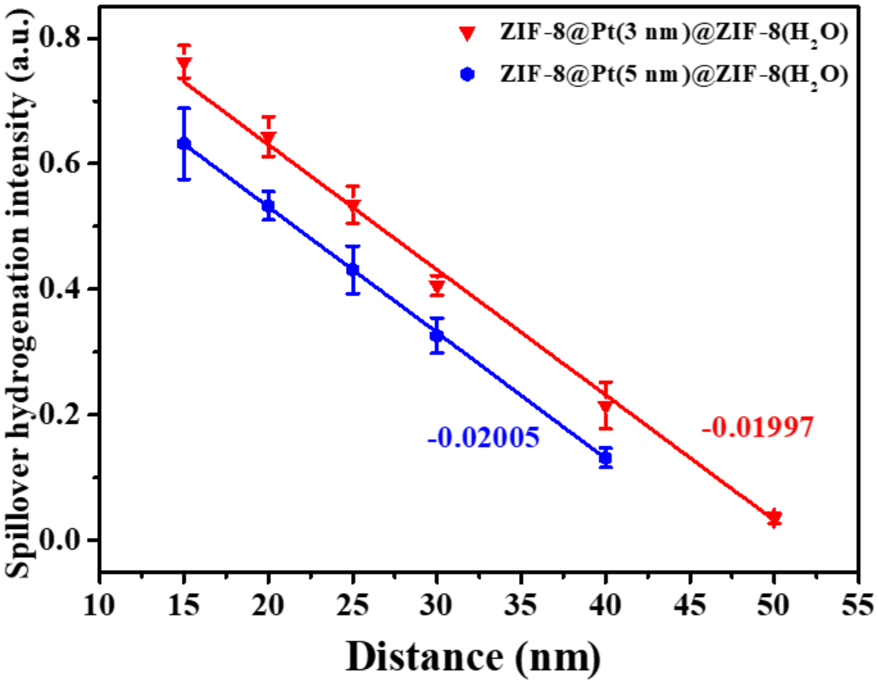

Supplementary Fig. 50 Linear relationship between spillover intensity and spillover distance of Zn-ZIF-8@Pt@Zn-ZIF-8 (H_2O) (80-minute reaction). All reactions were performed using catalysts with the same amount of Pt NPs.

The sandwich catalysts with large-sized Pt NPs (ca. 5 nm) exhibit nearly the same spillover decay factor as the above-used one but lower hydrogenation activity, meaning that particle size only affects hydrogen activation independent of spillover intensity.

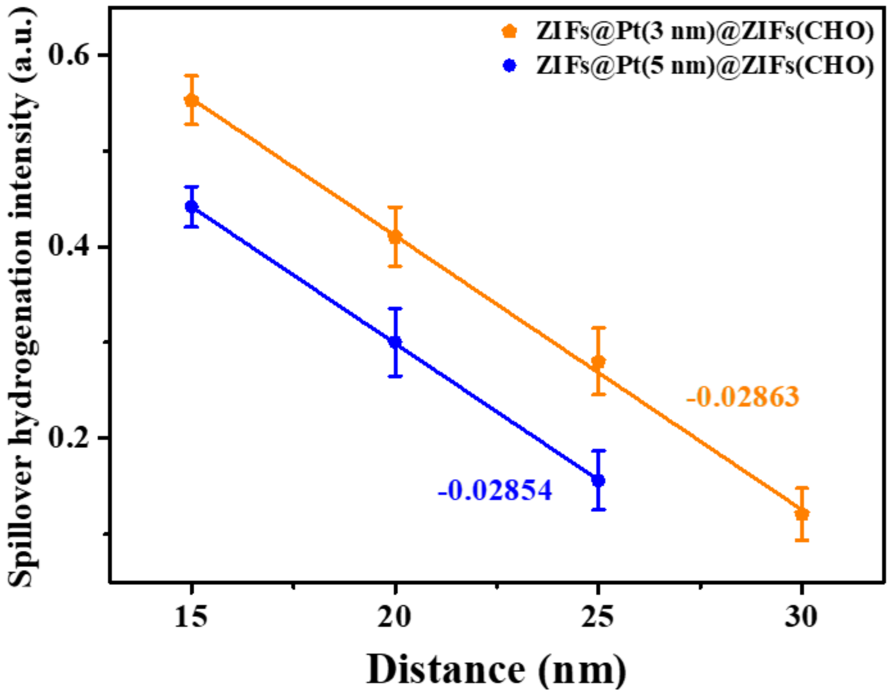

Supplementary Fig. 51 Linear relationship between spillover intensity and spillover distance of Zn-ZIFs@Pt@Zn-ZIFs (CHO) (80-minute reaction). All reactions were performed using catalysts with the same amount of Pt NPs.

The sandwich catalysts with large-sized Pt NPs (ca. 5nm) exhibit nearly the same spillover decay factor as the above-used one but lower hydrogenation activity, meaning that particle size only affects hydrogen activation independent of spillover intensity.

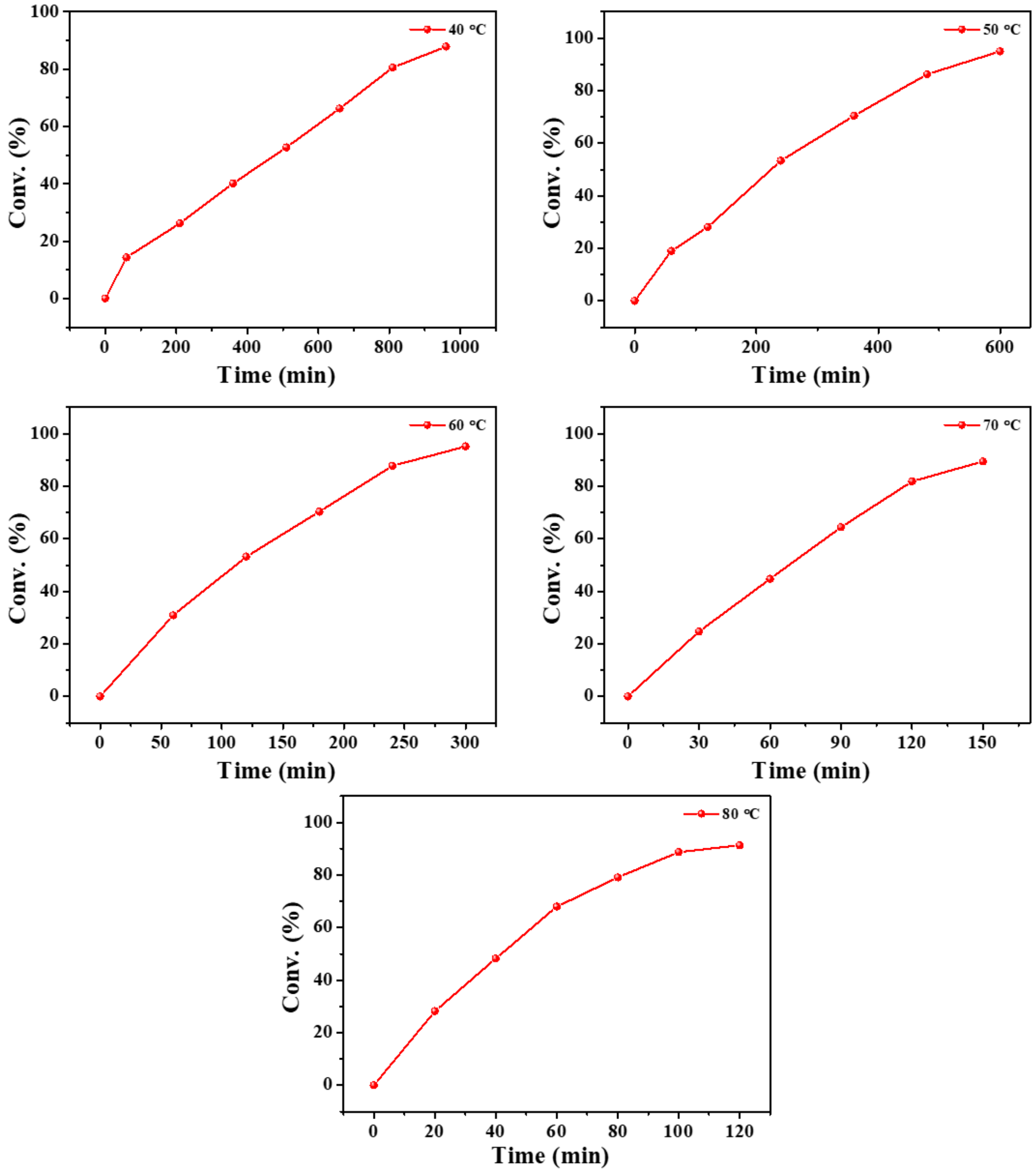

Supplementary Fig. 52 Catalytic performance of cyclooctene hydrogenation by Zn-ZIF-8@Pt@Zn-ZIF-8 (H_2O) catalyst at various reaction temperatures.

No solvent

Reductant: 2 MPa H_2

Shell thickness: 15 nm

Reactant: 0.5 mL cyclooctene

Catalyst: Zn-ZIF-8@Pt@Zn-ZIF-8 (H_2O)

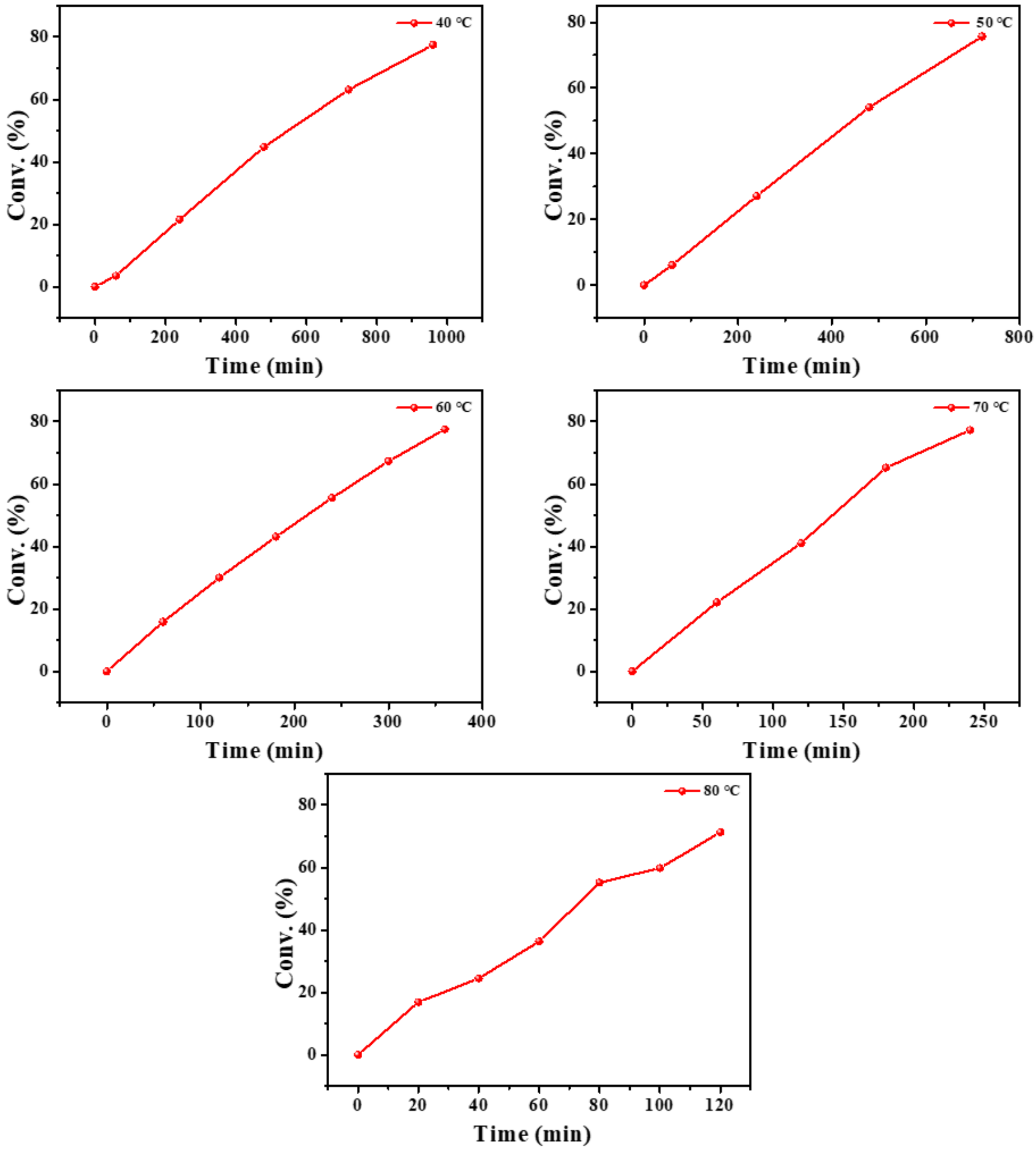

Supplementary Fig. 53 Catalytic performance of cyclooctene hydrogenation by Zn-ZIFs@Pt@Zn-ZIFs (CHO) catalyst at various reaction temperatures.

No solvent

Reductant: 2 MPa H_2

Shell thickness: 15 nm

Reactant: 0.5 mL cyclooctene

Catalyst: Zn-ZIFs@Pt@Zn-ZIFs (CHO)

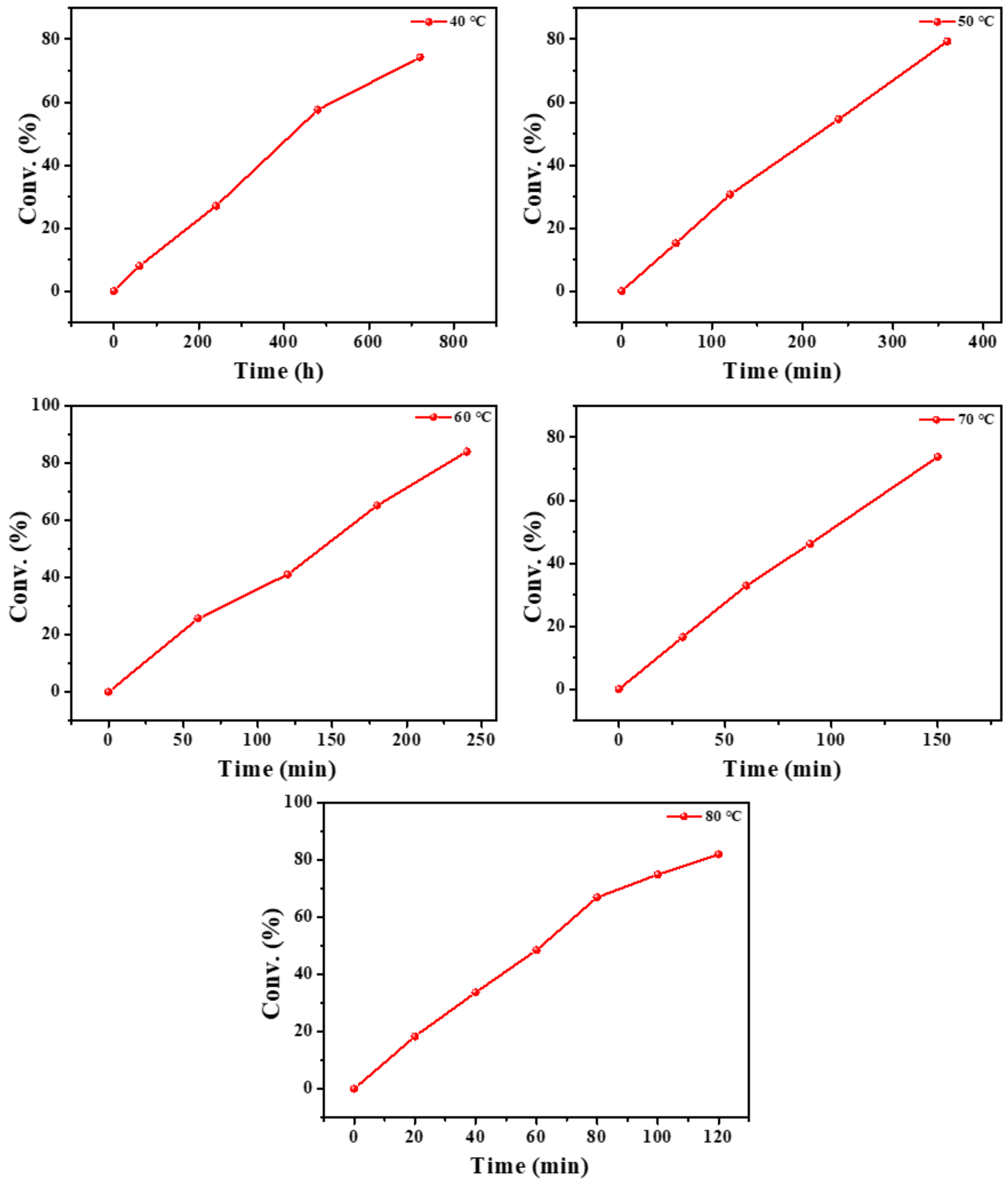

Supplementary Fig. 54 Catalytic performance of cyclooctene hydrogenation by Zn-ZIFs@Pt@Zn-ZIFs (OH) catalyst at various reaction temperatures.

No solvent

Reductant: 2 MPa H_2

Shell thickness: 15 nm

Reactant: 0.5 mL cyclooctene

Catalyst: Zn-ZIFs@Pt@Zn-ZIFs (OH)

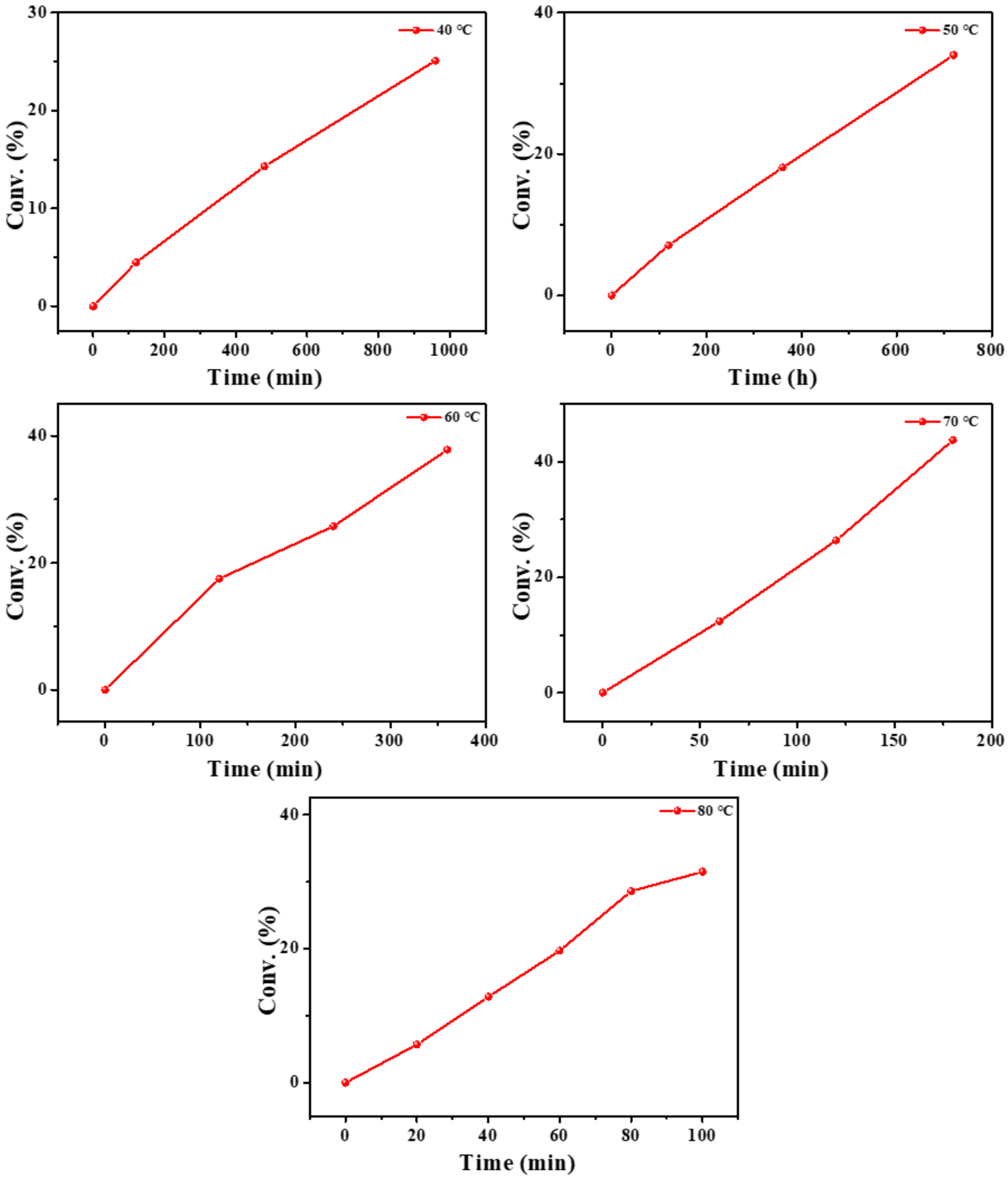

Supplementary Fig. 55 Catalytic performance of cyclooctene hydrogenation by Zn-ZIFs@Pt@Zn-ZIFs (NH_2) catalyst at various reaction temperatures.

No solvent

Reductant: 2 MPa H_2

Shell thickness: 15 nm

Reactant: 0.5 mL cyclooctene

Catalyst: Zn-ZIFs@Pt@Zn-ZIFs (NH_2)

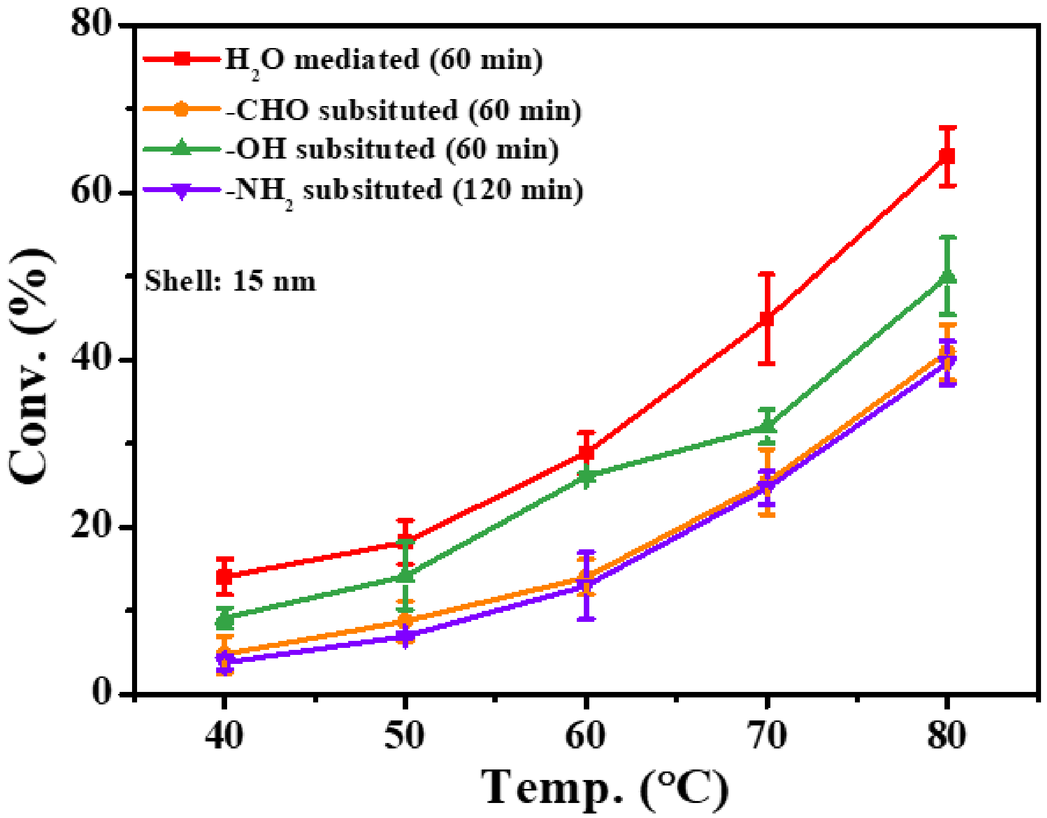

Supplementary Fig. 56 Conversion of cyclooctene by Zn-ZIFs@Pt@Zn-ZIFs homologs catalysts at different temperatures.

No solvent

Reductant: 2 MPa H_2

Shell thickness: 15 nm

Reactant: 0.5 mL cyclooctene

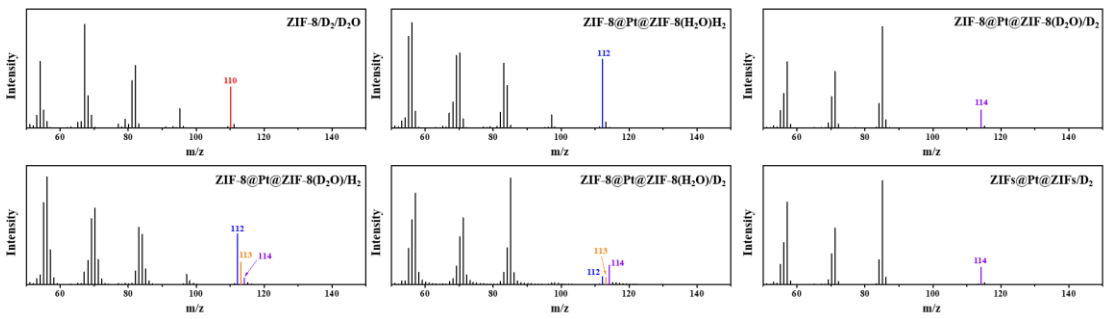

Supplementary Fig. 57 Mass spectrometry of the reaction products.

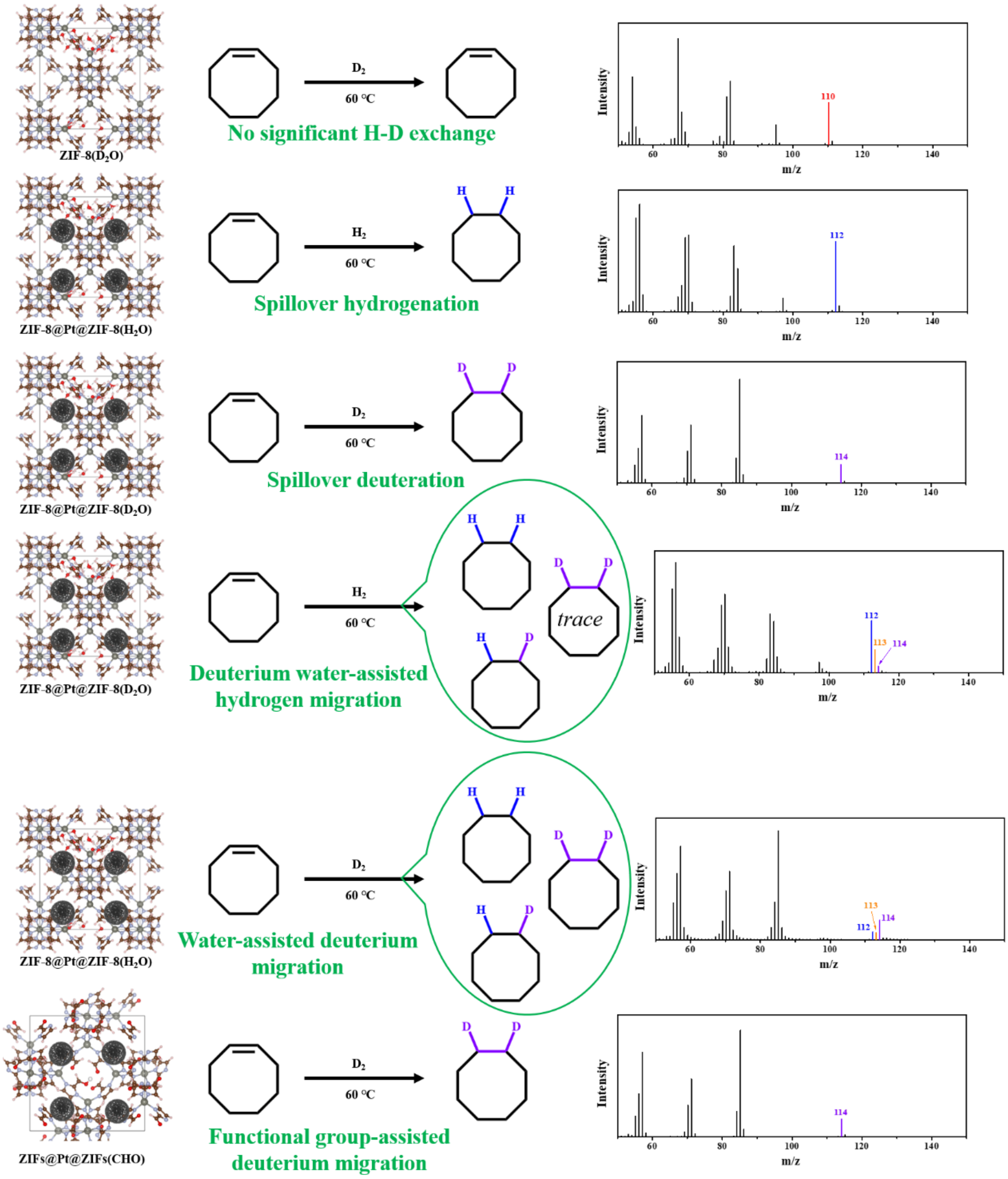

Supplementary Fig. 58 Deuterium labeling experiments. Analysis of reaction products in different systems.

The hydrogenation and deuteration of cyclooctene are hardly detected in Zn-ZIF-8(D_2O)-D_2 system (the top line), indicating that H-D exchange on probe cyclooctene is difficult to occur under the mild condition. At the same time, complete spillover hydrogenation and spillover deuteration can be clearly observed in Zn-ZIF-8@Pt@Zn-ZIF-8 (H_2O)-H_2 system (the second line from the top) and Zn-ZIF-8@Pt@Zn-ZIF-8 (D_2O)-D_2 system (the third line from the top), respectively. Interestingly, whether in Zn-ZIF-8@Pt@Zn-ZIF-8 (D_2O)-H_2 system (the fourth line from the top) or in Zn-ZIF-

8@Pt@Zn-ZIF-8 (H_2O)-D_2 system (the fifth line from the top), cyclooctene is hydrogenated to cyclooctane, [D1]-cyclooctane and [D_2]-cyclooctane. The above analysis demonstrates that H_2 splitting occurs on Pt NPs, and the activated hydrogen atoms diffuse across MOFs structure by the water-assist path accompanied by exchange with water. In addition, as for Zn-ZIFs@Pt@Zn-ZIFs (CHO)-D_2 system (the bottom line), the product of [D_2]-cyclooctane clarifies the migration of D atoms across MOFs containing CHO functional groups.

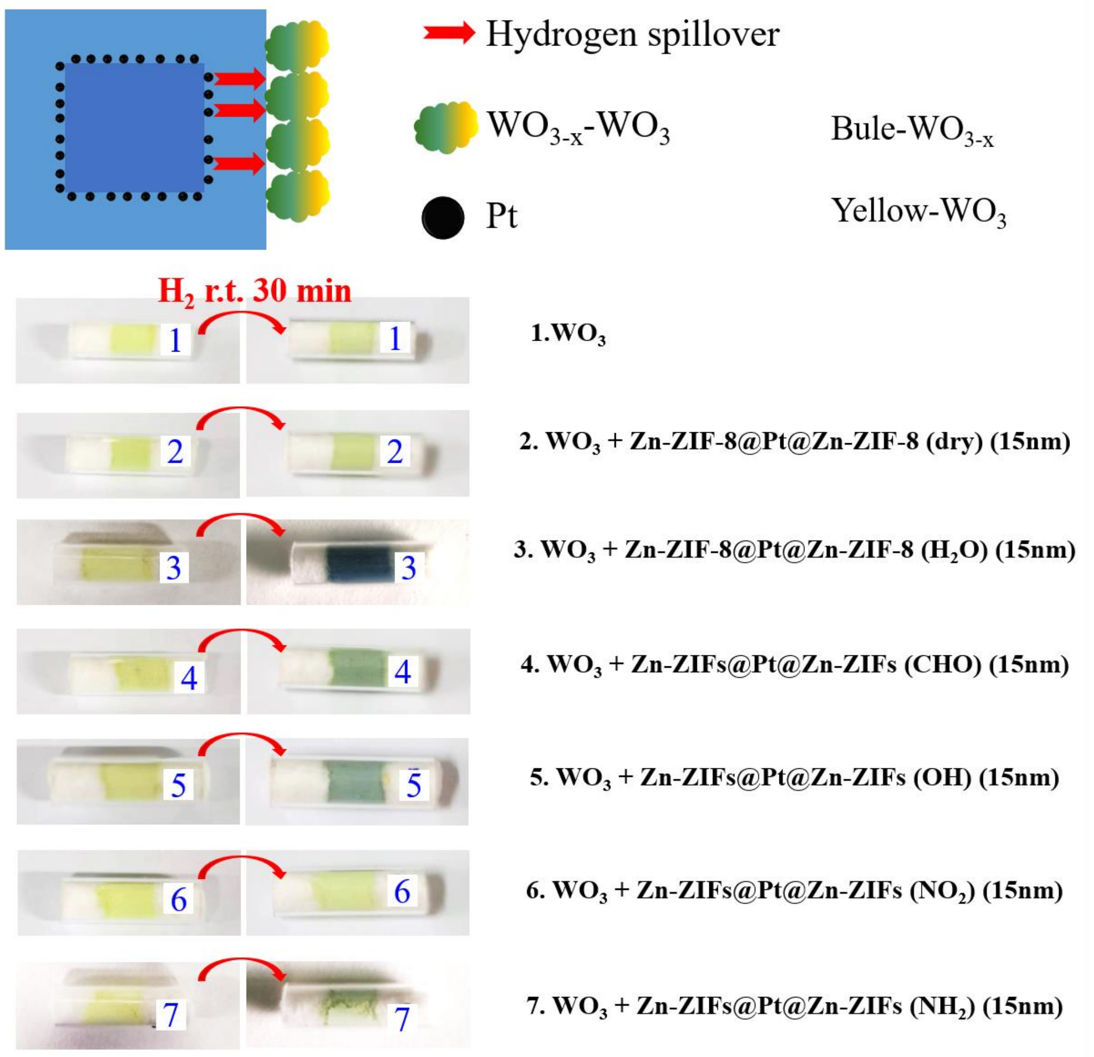

Supplementary Fig. 59 Top: scheme of WO_3 discoloration caused by hydrogen spillover. Down: photographs of samples of 1 g WO_3 mixed with 0.04 g ZIFs@Pt@ZIFs homologs before and after treatment with H_2 at room temperature for 0.5 h.

WO_3 was used to visually confirm the H_2 activation by Pt nanoparticles and subsequent hydrogen spillover to Zn-ZIFs at low temperature, in which the activated H species could readily react with the bright yellow WO_3 to form dark blue H_xWO_3. The Zn-ZIFs@Pt@Zn-ZIFs homologs (OH, CHO, NH_2) and Zn-ZIF-8@Pt@Zn-ZIF-8 (H_2O) catalysts endow the dark blue color of WO_3 after H_2 treatment at room temperature. In contrast, WO_3 alone as well as WO_3 mixed with Zn-ZIF-8@Pt@Zn-ZIF-8 (dry) and Zn-ZIFs@Pt@Zn-ZIFs (NO_2) exhibit bare color change after hydrogen treatment under the same condition. Thus, the color evolution of WO_3 demonstrates that the H_2

activation, dissociation and hydrogen spillover can successfully occur on the functionalized Zn-ZIFs@Pt@Zn-ZIFs homologs (OH, CHO, NH_2) and Zn-ZIF-8@Pt@Zn-ZIF-8 (H_2O).

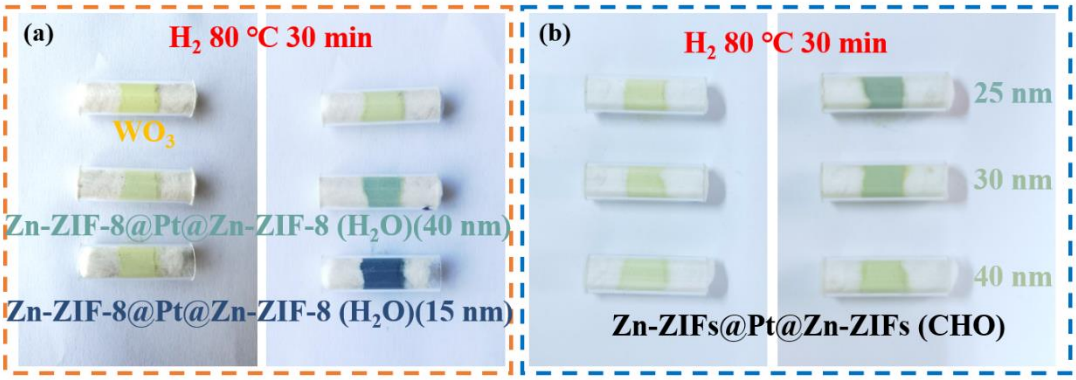

Supplementary Fig. 60 (a) Photographs of samples of 1 g WO_3 mixed with 0.04 g Zn-ZIF-8@Pt@Zn-ZIF-8 (H_2O) before and after treatment with H_2 at 80°C for 0.5 h. (b) Photographs of samples of 1 g WO_3 mixed with 0.04 g Zn-ZIFs@Pt@Zn-ZIFs (CHO) before and after treatment with H_2 at 80°C for 0.5 h.

The color change uncovers a significant spillover distance (MOFs shell thickness) dependence. Under the same condition, the thinner shell thickness gives rise the darker blue color of sandwich sample.

Table S5 EXAFS fitting parameters at Zn K edge (S_0^2 = 0.97)

Sample	Path	C.N.	R (Å)	$\sigma^2 \times 10^3$ (Å^2)	ΔE (eV)	R factor
ZnO	Zn-O	4*	1.96±0.02	6.0±2.3	1.8±3.7	0.017
	Zn-Zn	12*	3.23±0.02	20.1±19.1	2.6±3.0	
1	Zn-N	3.8±0.6	1.99±0.01	4.2±1.2	5.2±1.7	0.015
2	Zn-N	3.9±0.6	1.99±0.01	4.9±1.3	5.3±1.6	0.014
3	Zn-N	3.8±0.6	1.99±0.01	4.6±1.2	5.2±1.6	0.014
4	Zn-N	4.3±0.6	2.00±0.01	5.6±1.1	4.0±1.4	0.011
5	Zn-N	4.2±0.5	2.00±0.01	6.2±1.1	3.7±1.3	0.009
6	Zn-N	4.1±0.4	2.00±0.01	5.7±1.0	3.9±1.2	0.007

C.N: coordination numbers; *R*: bond distance; σ^2: Debye-Waller factors; Δ*E*: the inner potential correction. *R* factor: goodness of fit. *fitting with fixed parameter.

1: Zn-ZIF-8@Pt@Zn-ZIF-8 (H_2O) air/r.t.

2: Zn-ZIF-8@Pt@Zn-ZIF-8 (H_2O) H_2/80℃

3: Zn-ZIF-8@Pt@Zn-ZIF-8 (H_2O) H_2/80℃/30 min

4: Zn-ZIFs@Pt@Zn-ZIFs (CHO) air/r.t.

5: Zn-ZIFs@Pt@Zn-ZIFs (CHO) H_2/80°C

6: Zn-ZIFs@Pt@Zn-ZIFs (CHO) H_2/80°C/30 min

The obtained XAFS data was processed in Athena (version 0.9.26) for background, pre-edge line and post-edge line calibrations. Then Fourier transformed fitting was carried out in Artemis (version 0.9.26). The k^3 weighting, k-range of 2 - 12.5 Å^{-1} and R range of 1 - 2 Å were used for the fitting. The four parameters including coordination number, bond length, Debye-Waller factor and E_0 shift (CN, R, σ^2, ΔE_0) were fitted without anyone was fixed, constrained or correlated.

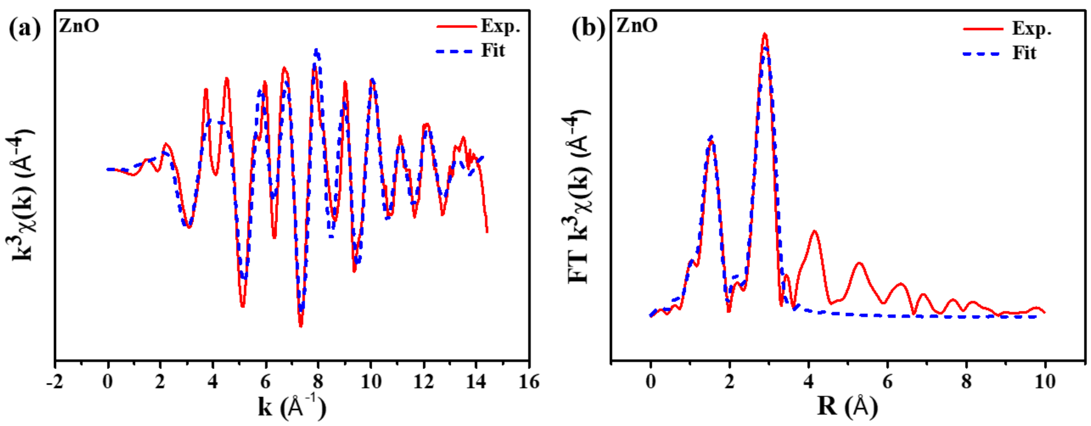

Supplementary Fig. 61 (a) K^3-weighted spectra in K space for ZnO, (b) Zn K-edge EXAFS data in R-space of ZnO.

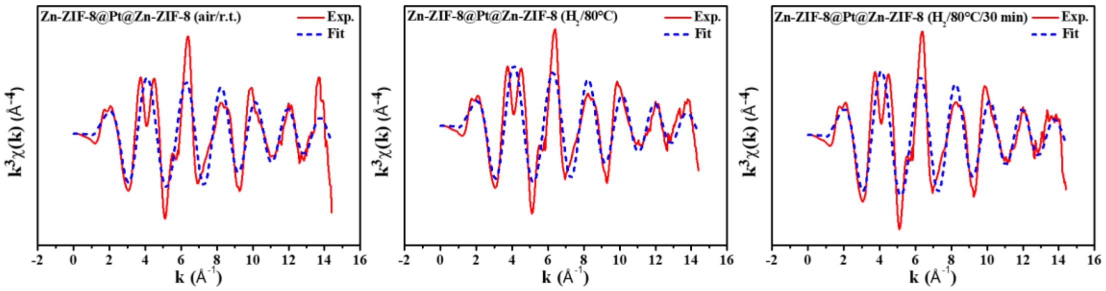

Supplementary Fig. 62 K^3-weighted spectra in K space for Zn-ZIF-8@Pt@Zn-ZIF-8 (H_2O) under different conditions.

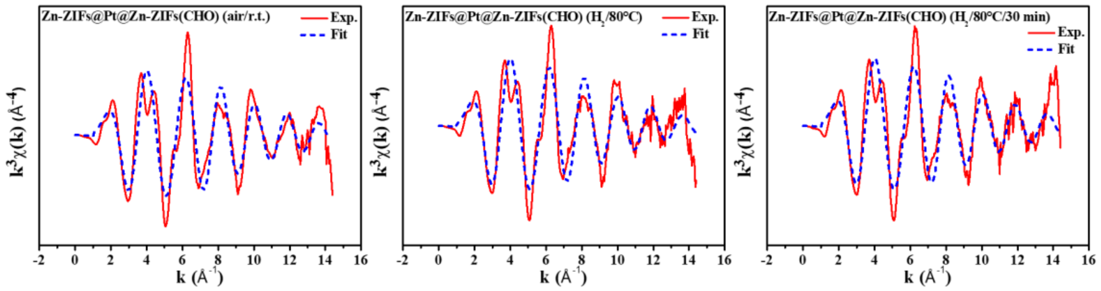

Supplementary Fig. 63 K^3-weighted spectra in K space for Zn-ZIFs@Pt@Zn-ZIFs (CHO) under different conditions.

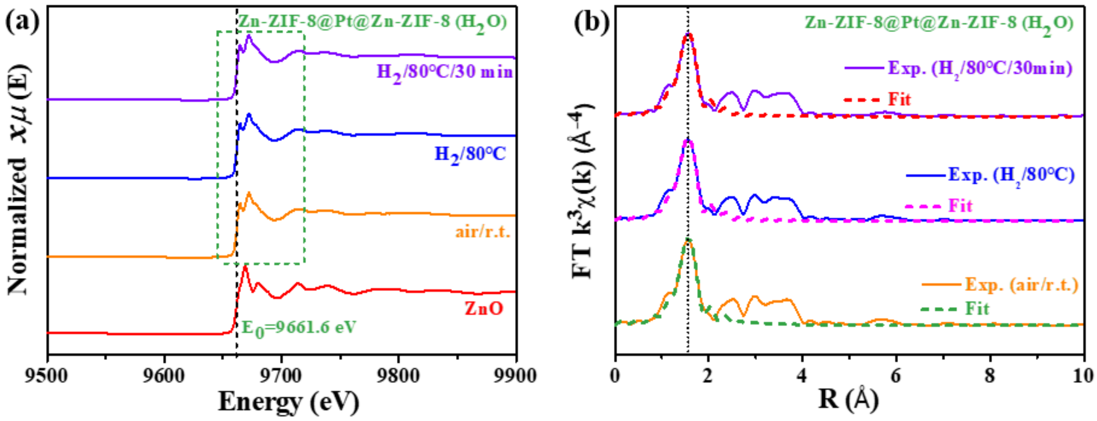

Supplementary Fig. 64 *In situ* XAS characterization of Zn-ZIF-8@Pt@Zn-ZIF-8 (H_2O). (a) Zn K-edge XANES and (b) Zn K-edge EXAFS spectra in R-space collected on as-prepared Zn-ZIF-8@Pt@Zn-ZIF-8 (H_2O) under air at room temperature or hydrogen at 80°C.

First, the Zn K-edge X-ray absorption near-edge structure (XANES) spectra of ZnO and as-prepared Zn-ZIF-8@Pt@Zn-ZIF-8 are recorded under ambient condition. As shown in Supplementary Fig. 64a, when Zn is coordinated by oxygen atoms in ZnO, the first XANES peak (9669.1 eV) dominates the spectrum. As comparison, zinc imidazole complexes (Zn-ZIF-8), in which nitrogen atoms coordinate the metal ion, show a significant increase in the second XANES peak intensity (9672.2 eV). Second, the X-ray absorption fine structure (EXAFS) data and the best-fit analysis are summarized in Supplementary Fig. 64b, and the dominant peak at 1.99 Å is assigned to the first shell coordination of M (metal)-N (Table S5). According to coordination analysis, one can see that the average coordination number of the M-N shell estimated by EXAFS is found to be four-coordination, which is in good agreement with those determined by XRD survey. Furthermore, both *in situ* XANES and *in situ* EXAFS results demonstrate that Zn K-edge peaks of Zn-ZIF-8@Pt@Zn-ZIF-8 (H_2O) barely change under hot hydrogen atmosphere, validating that hydrogen spillover occurring on Zn-ZIFs does not involve any change in Zn oxidation state and coordination environment.

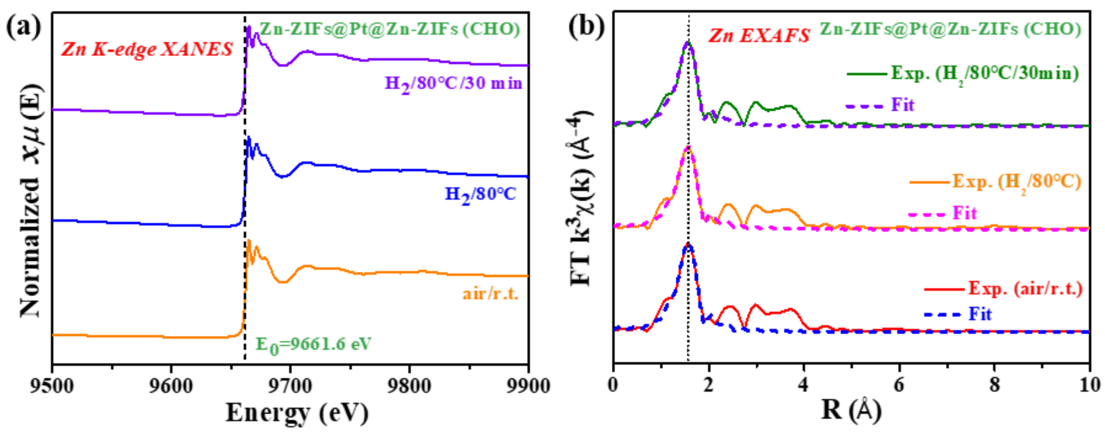

Supplementary Fig. 65 *In situ* XAS characterization of Zn-ZIFs@Pt@Zn-ZIFs (CHO). (a) Zn K-edge XANES and (b) Zn K-edge EXAFS spectra in R-space collected on as-prepared Zn-ZIFs@Pt@Zn-ZIFs (CHO) under air at room temperature or hydrogen at 80°C.

Both *in situ* XANES and *in situ* EXAFS results demonstrate that Zn K-edge peaks of Zn-ZIFs@Pt@Zn-ZIFs (CHO) barely change under hot hydrogen atmosphere, validating that hydrogen spillover occurring on Zn-ZIFs does not involve any change in Zn oxidation state and coordination environment.

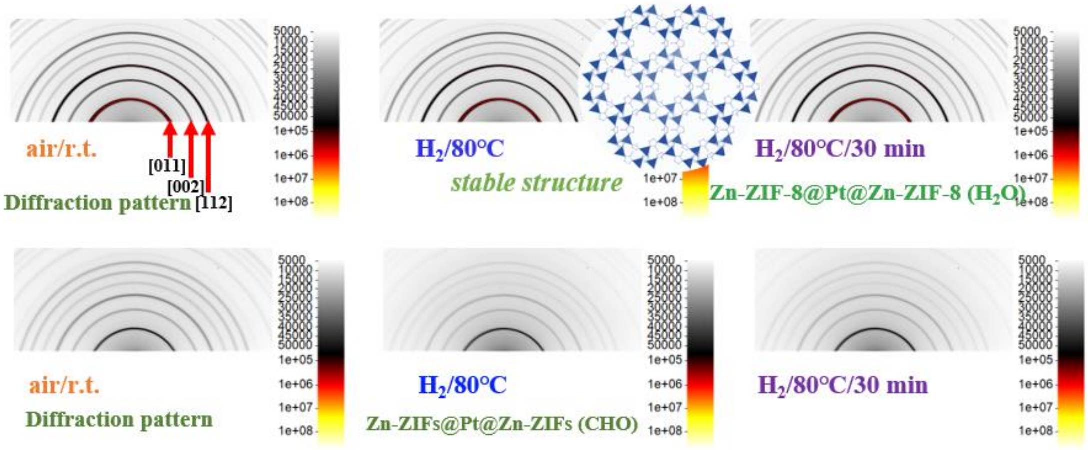

Supplementary Fig. 66 *In situ* XRD patterns of as-prepared Zn-ZIFs@Pt@Zn-ZIFs under air at room temperature or hydrogen at 80°C (0 min and 30 min) (Part is given in Fig. 4c).

The corresponding XRD patterns show that Zn-ZIF-8@Pt@Zn-ZIF-8 (H_2O) and Zn-ZIFs@Pt@Zn-ZIFs (CHO) maintain ultra-high structural stability during whole H_2-heat-process.

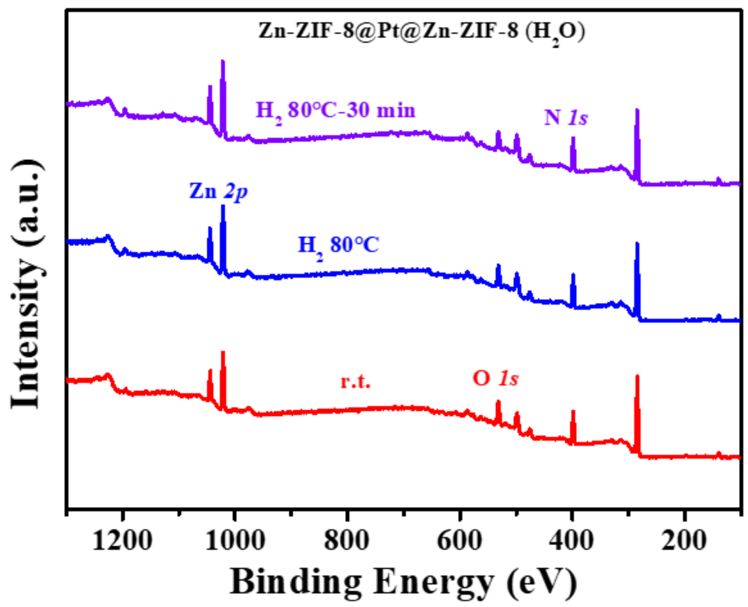

Supplementary Fig. 67 *In situ* XPS survey spectra of Zn-ZIF-8@Pt@Zn-ZIF-8 (H_2O) at as-prepared and spillover state.

X-ray photoelectron spectroscopy (XPS) analysis indicates that the major composition elements of Zn-ZIF-8@Pt@Zn-ZIF-8 (H_2O) include C, N, Zn and O.

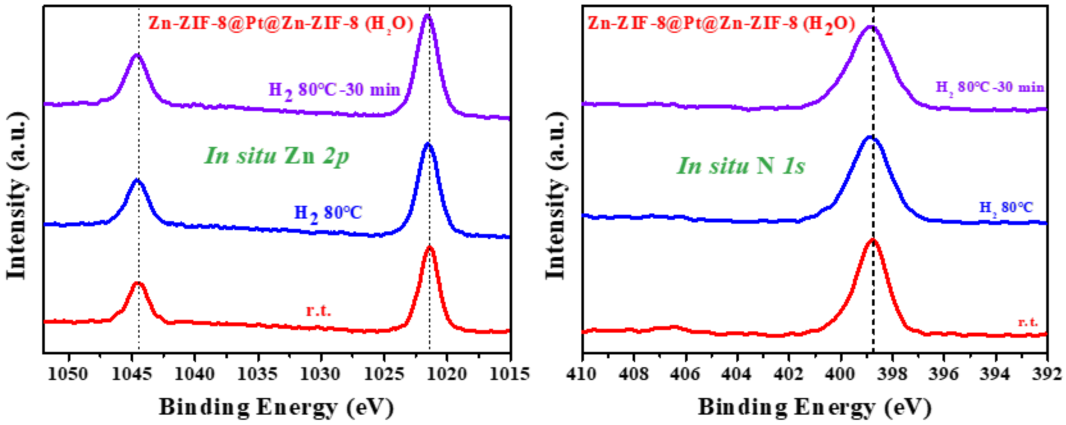

Supplementary Fig. 68 *In situ* XPS spectra (Zn *2p* and O *1s*) of Zn-ZIF-8@Pt@Zn-ZIF-8 (H_2O) at as-prepared and spillover state.

The Zn *2p* and N *1s* peaks of as-prepared Zn-ZIF-8@Pt@Zn-ZIF-8 (H_2O) are located at 1021/1044 eV and 389.8 eV, respectively, and these peaks remain almost unaltered after interacting hydrogen atoms generated on Pt. The slight shift of these peaks is likely due to the introduction of H (or electron) in the system that affects the Zn-N bond.

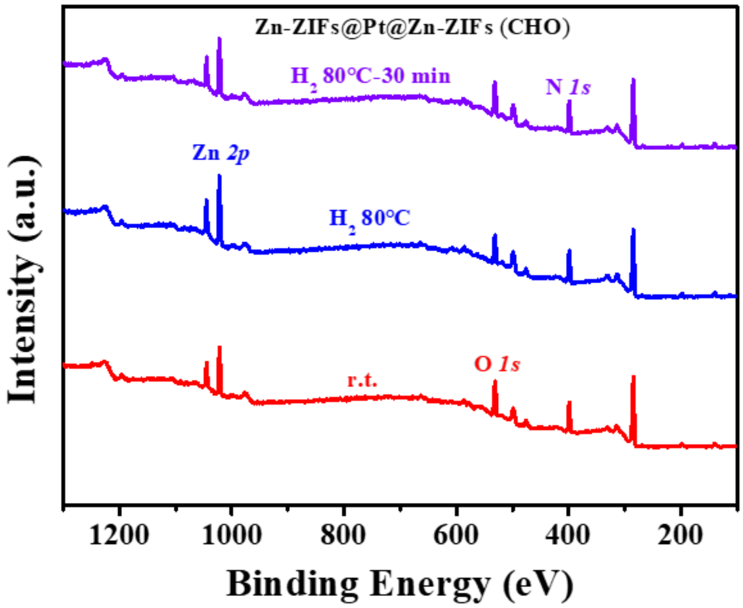

Supplementary Fig. 69 *In situ* XPS survey spectra of Zn-ZIFs@Pt@Zn-ZIFs (CHO) at as-prepared and spillover state.

X-ray photoelectron spectroscopy (XPS) analysis indicates that the major composition elements of Zn-ZIFs@Pt@Zn-ZIFs (CHO) include C, N, Zn and O.

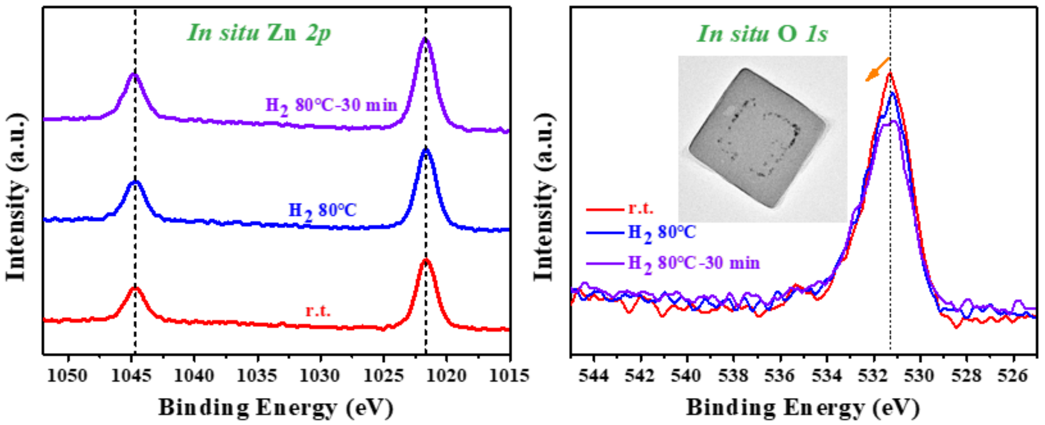

Supplementary Fig. 70 *In situ* XPS spectra (Zn *2p* and O *1s*) of Zn-ZIFs@Pt@Zn-ZIFs (CHO) at as-prepared and spillover state.

The Zn *2p* peaks of Zn-ZIFs@Pt@Zn-ZIFs (CHO) always remain unchanged during the entire *in situ* test, while the corresponding O *1s* peaks shift to higher energy in the spillover state, indicating that the O species of aldehyde group is the possible binding site of H atom.

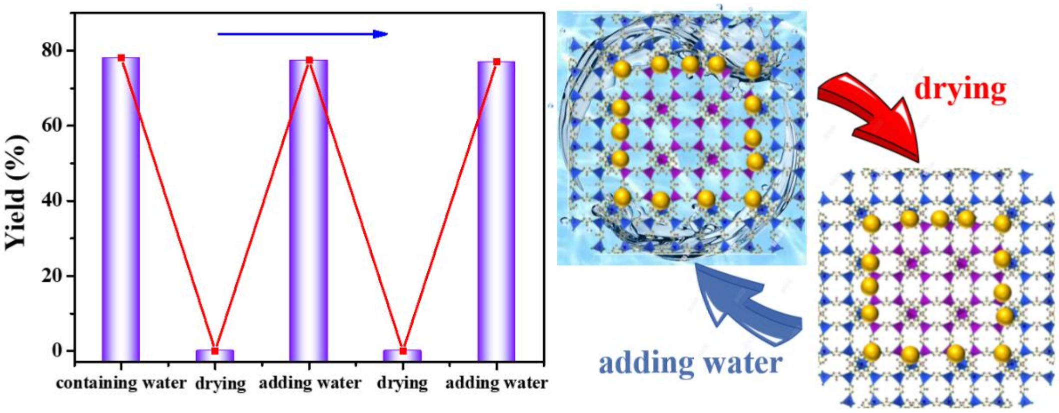

Supplementary Fig. 71 Effect of water on hydrogen spillover. Spillover hydrogenation yield of cyclooctene over water-contained and dried Zn-ZIF-8@Pt@Zn-ZIF-8 catalysts.

The water-containing catalyst delivers quite high catalytic activity for cyclooctene hydrogenation, whereas the water-free catalyst is almost inactive.

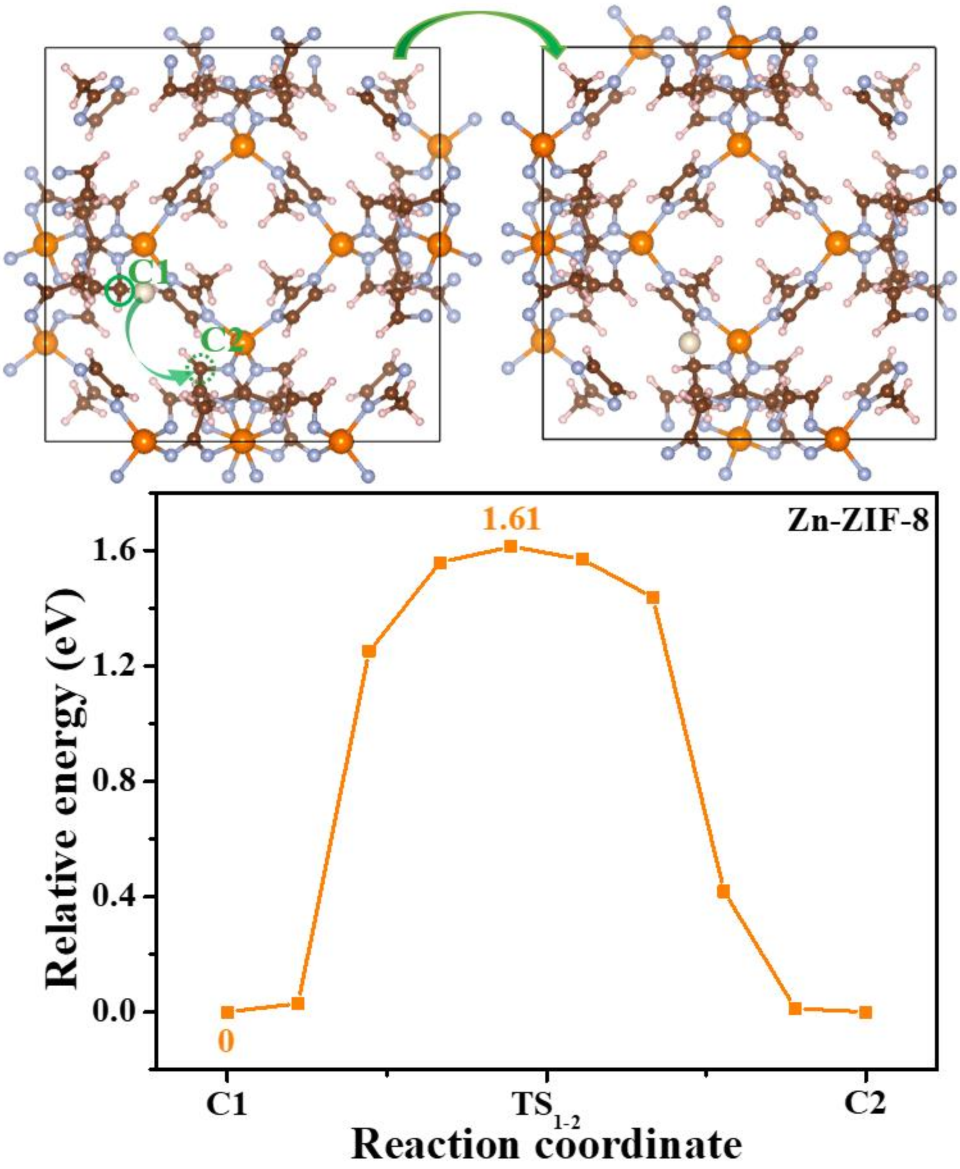

Supplementary Fig. 72 Hydrogen spillover in Zn-ZIF-8.

As shown in top panel, hydrogen atoms migrate via the step C1 to C2. The calculated energy barrier of corresponding transition state (TS_{1-2}) is 1.61 eV (bottom panel).

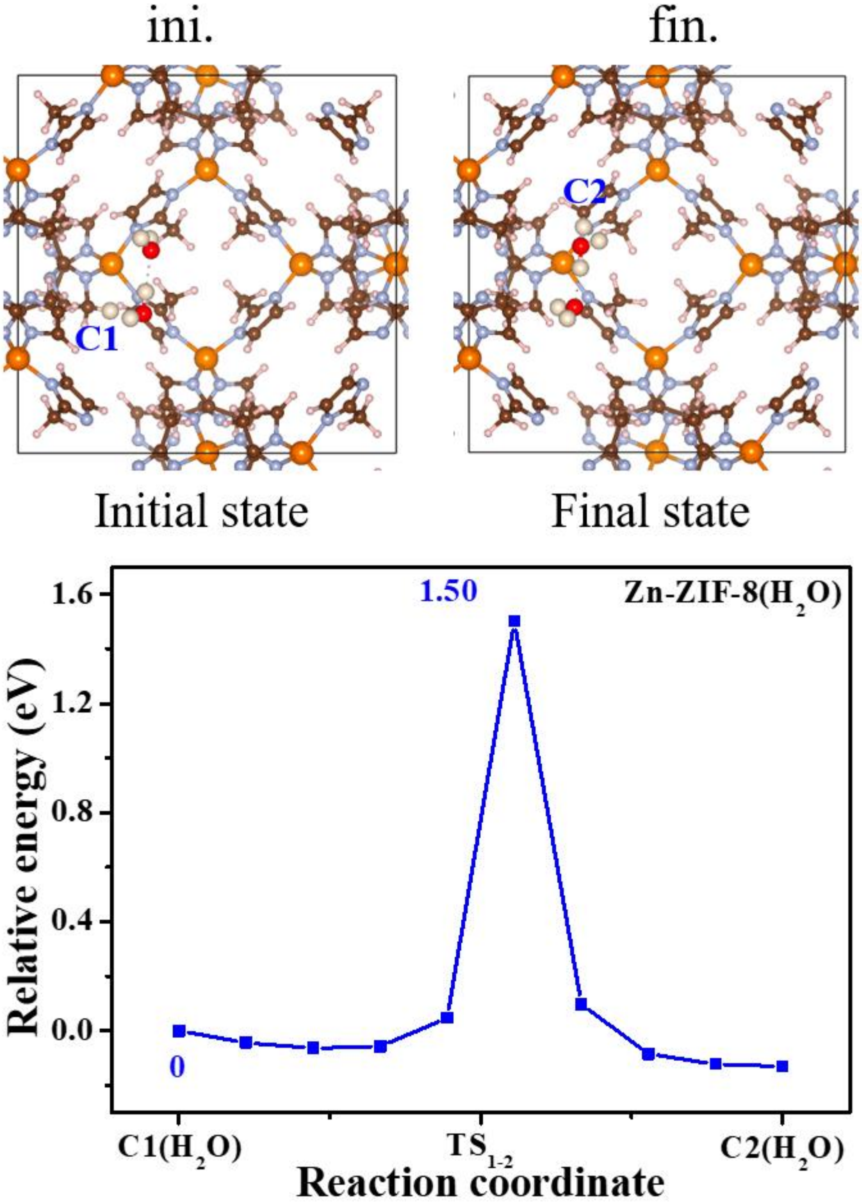

Supplementary Fig. 73 Hydrogen spillover in Zn-ZIF-8.

As shown in top panel, hydrogen atoms migrate in the form of H_3O· via the step C1 (H_2O) to C2 (H_2O). The calculated energy barrier of corresponding transition state (TS_{1-2}) is 1.5 eV (bottom panel).

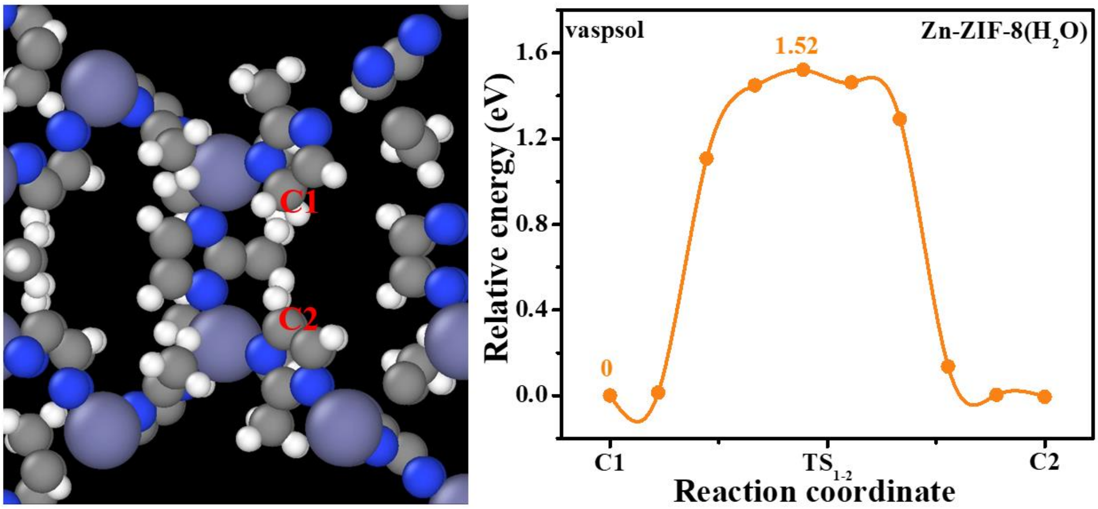

Supplementary Fig. 74 Hydrogen spillover in Zn-ZIF-8 (H_2O).

If hydrogen atoms migrate in the water environment (calculated by VASPsol method), the corresponding energy barrier is 1.52 eV.

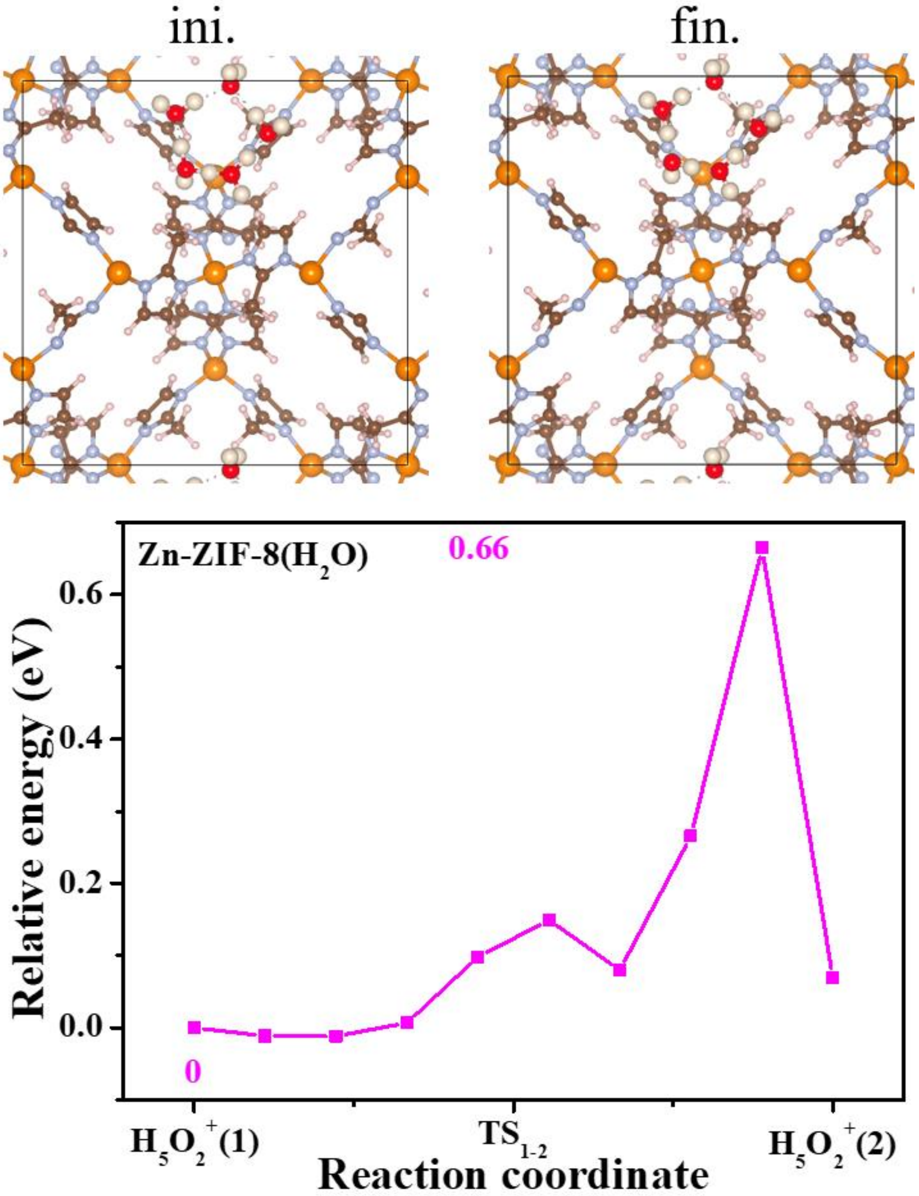

Supplementary Fig. 75 Water-assisted proton hopping via Zundel cation $H_5O_2^+$.

Electron migrates along framework of Zn-ZIF-8 simultaneously with water-assisted proton hopping, and this process is obviously affected by the number of water molecules. This value increases to 0.66 eV when the calculation model involves two water molecules (i.e., $H_5O_2^+$). This result well explains the reduced catalytic activity of the recovered sample (Figure 3c).

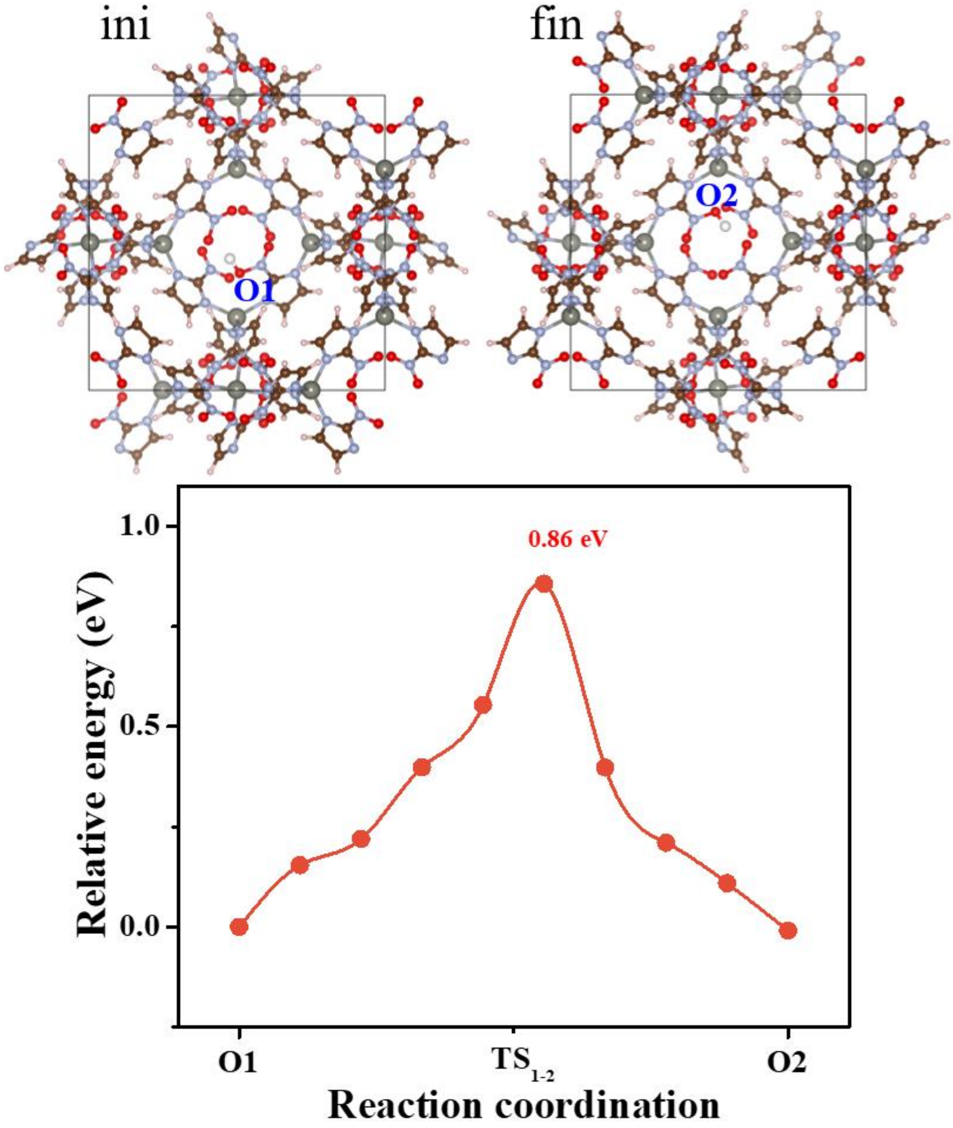

Supplementary Fig. 76 Hydrogen spillover in Zn-ZIFs (NO_2).

As for nitro-ZIFs, although H also migrates through the O··H··O pathway, its migration barrier is as high as 0.86 eV, which also explains its low spillover hydrogenation activity.

Table S6 Conversion ratio and selectivity of 5-chloroquinolines catalyzed by various catalysts.

Cata.	Temp. (°C)	Conv. (%)	Sel.-a (%)
Pt nanoparticles	80	99.7	75.5
	80	99.9	83.2
	80	99.3	80
Pt/C	80	99.7	46.5
	80	99.8	47.5
	80	99.8	51.2
Pt/TiO_2	80	48.2	96.6
	80	53.7	94.7
	80	55.6	93.7
Pt/Al_2O3	80	92.3	89.3
	80	97.5	93.1
	80	98.3	91.6
Zn-ZIF-8@Pt@Zn-ZIF-8 (dry)	100	0	-
Zn-ZIF-8@Pt@Zn-ZIF-8 (H_2O)	80	99.5	99.9
	80	99.8	99.6
	80	99.6	99.3
Zn-ZIFs@Pt@Zn-ZIFs (CHO)	80	99.9	99.9
	80	96.1	99.3
	80	97	99.9

Solvent: isopropanol

Reductant: 2 MPa H_2

Time: 240 min

Reactant: 0.5 mmol 5-chloroquinolines

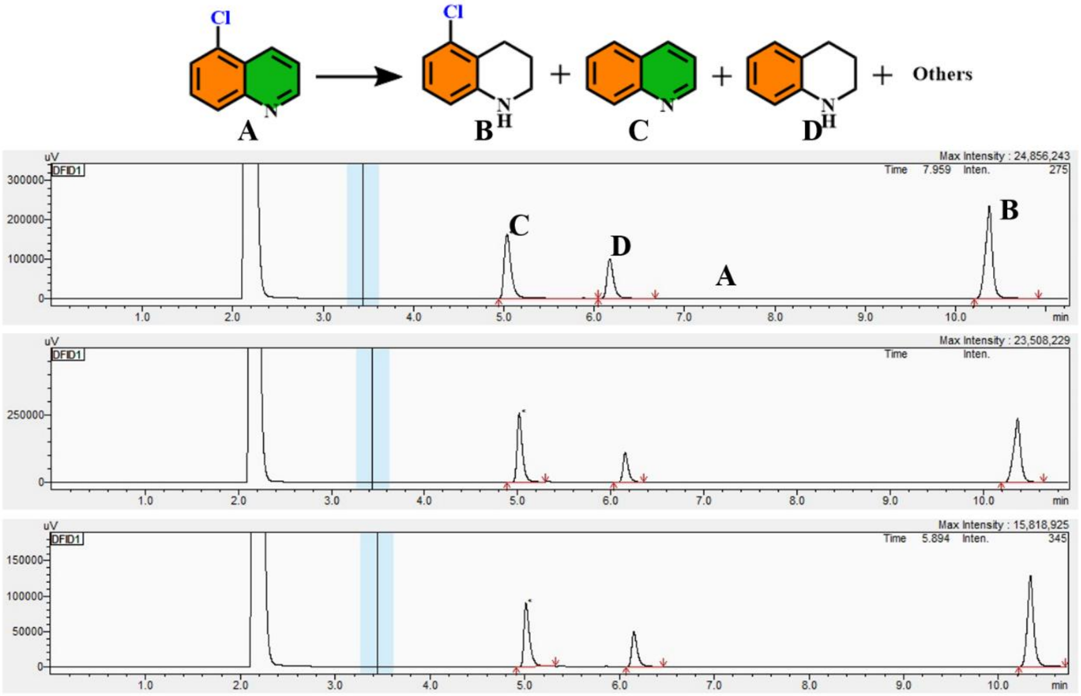

Supplementary Fig. 77 Raw GC data for selective hydrogenation of 5-chloroquinolines by Pt/C.

Peaks for	Retention time	Peak area	Height	Peak area/%
**A**	7.423	0	0	0
**B**	10.375	1256400	233388	47.535
**C**	5.035	883397	161478	33.423
**D**	6.173	503285	100015	19.042
**Total**		2643083	494882	100.000

Peaks for	Retention time	Peak area	Height	Peak area/%
**A**	7.423	0	0	0
**B**	10.371	1221796	233495	46.504
**C**	5.017	982219	254046	37.385
**D**	6.155	423291	108890	16.111
**Total**		2627306	596430	100.000

Peaks for	Retention time	Peak area	Height	Peak area/%
**A**	7.423	0	0	0
**B**	10.338	590810	128389	51.137
**C**	5.013	366092	89216	31.687
**D**	6.152	198437	48528	17.176
**Total**		2643083	494882	100.000

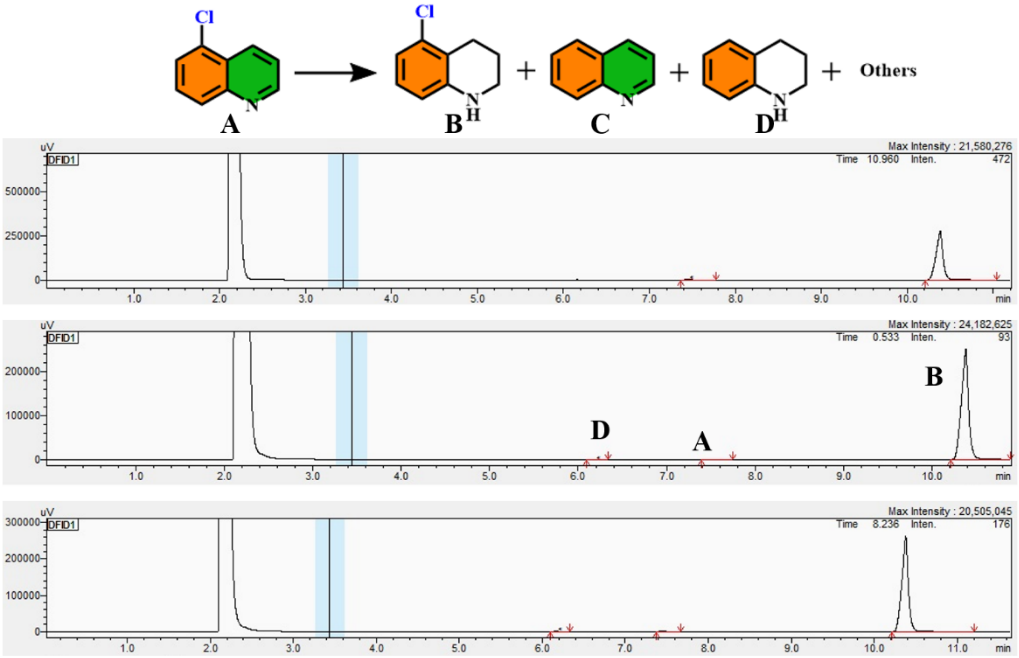

Supplementary Fig. 78 Raw GC data for selective hydrogenation of 5-chloroquinolines by Zn-ZIF-8@Pt@Zn-ZIF-8 (H_2O).

The Zn-ZIF-8@Pt@Zn-ZIF-8 (H_2O) catalysts deliver not only an equivalent catalytic activity to the reference Pt nanoparticles and Pt/C catalysts but also an unprecedented high selectivity of >99% to the primary product of 5-chloro-1,2,3,4-tetrahydroquinoline under the same reaction condition.

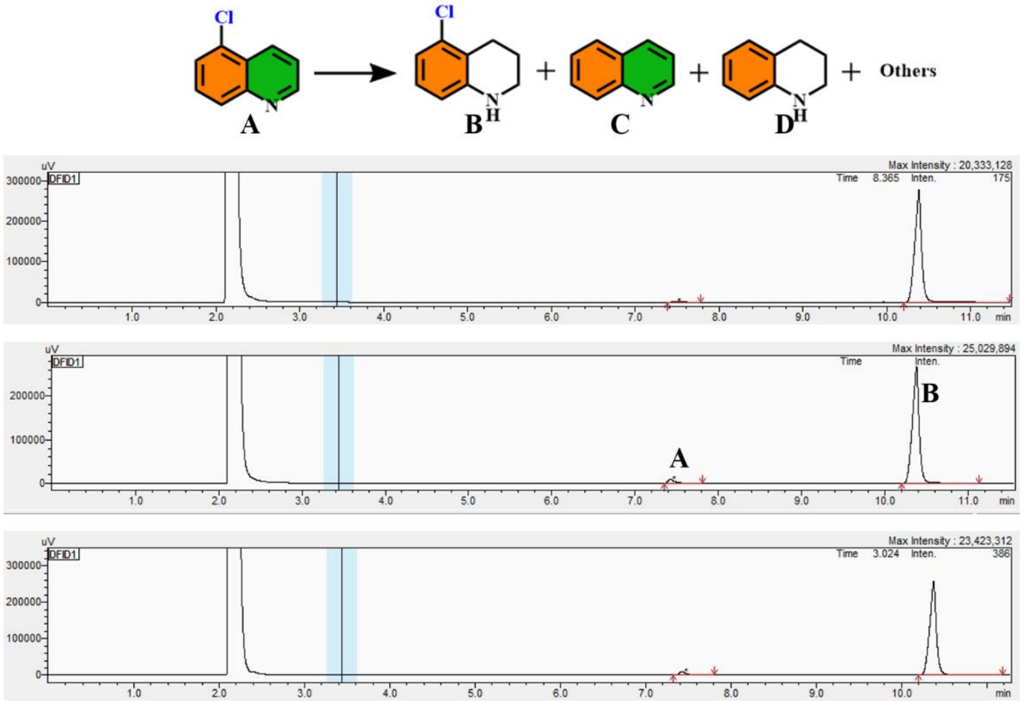

Supplementary Fig. 79 Raw GC data for selective hydrogenation of 5-chloroquinolines by Zn-ZIFs@Pt@Zn-ZIFs (CHO).

The Zn-ZIFs@Pt@Zn-ZIFs (CHO) catalysts deliver not only an equivalent catalytic activity to the reference Pt nanoparticles and Pt/C catalysts but also an unprecedented high selectivity of >99% to the primary product of 5-chloro-1,2,3,4-tetrahydroquinoline under the same reaction condition.

Table S7 Comparison of conversion ratio and selectivity of chloroquinolines catalyzed by the-state-of-the art catalysts.

Cata.	Temp. (°C)	P (MPa)	Time (h)	Conv. (%)	Sel. (%)	Ref.
Co_3O_4-Co/ NGr@α-Al_2O_3	120	2	48	99	81	1
$Au_{0.9}Pd_{0.1}$/CNR	100	2	5	85.3	89.3	2
CoO_x@CN	110	3	3	99	94	3
Au/HSA-TiO_2	60	2	3	98	100	4
Pt/HSA-TiO_2	60	2	3	97	92	4
Pd/HSA-TiO_2	60	2	3	92	86	4
Pt/*NR*-CeO_2	25	2	5	99.6	96.9	5
Co@C	80	2	4	99	99	6
Co-SA/AC @N-CNTs-L	100	2	3	96.2	99	7
Zn-ZIF-8@Pt@ Zn-ZIF-8 (H_2O)	80	2	4	99.6	99.6	This work
Zn-ZIFs@Pt@Zn-ZIFs (CHO)	80	2	4	97.6	99	This work

1. *J. Am. Chem. Soc.,* **2015,** *137*, 11718.

2. *J. Mater. Chem. A,* **2017**, *5,* 3260-3266.

3. *ACS Catal.* **2016,** *6,* 5816–5822

4. *J. Am. Chem. Soc.* **2012**, *134,* 17592– 17598

5. *J. Catal.* **2018,** *359,* 101-111.

6. *Chem* **2020,** *6,* 2994-3006.

7. Adv. Mater.**2019,** *31,* 1906051.

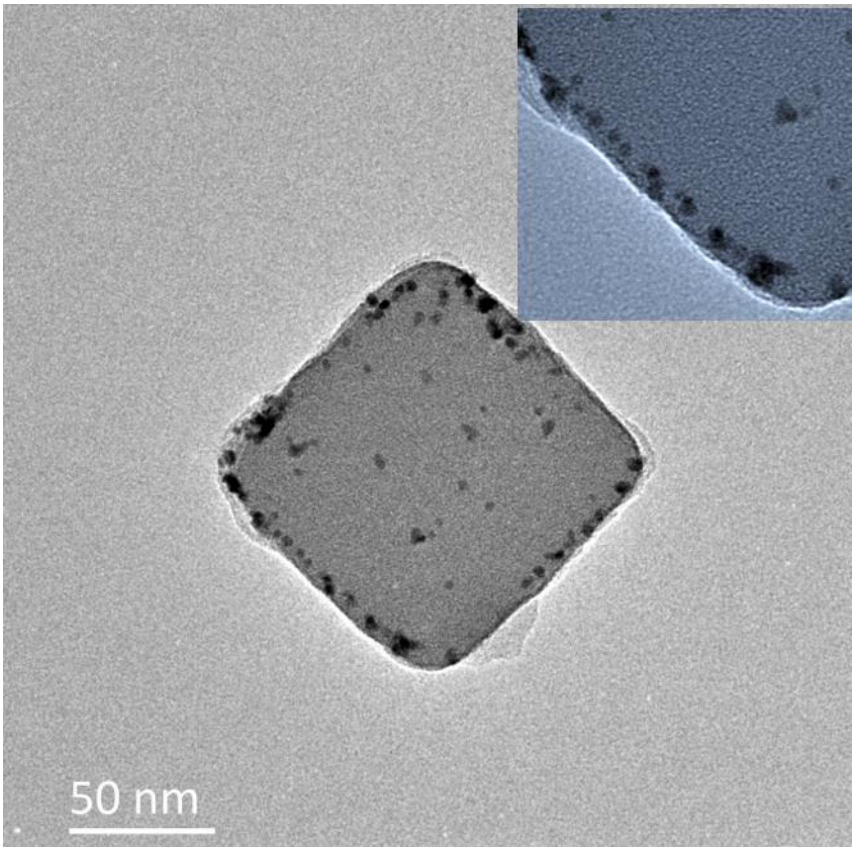

Supplementary Fig. 80 TEM image of Zn-ZIF-8@Pt@Zn-ZIF-8 with incomplete shell.

SEM image shows that Pt nanoparticles are incompletely coated in the Zn-ZIF-8 shell.

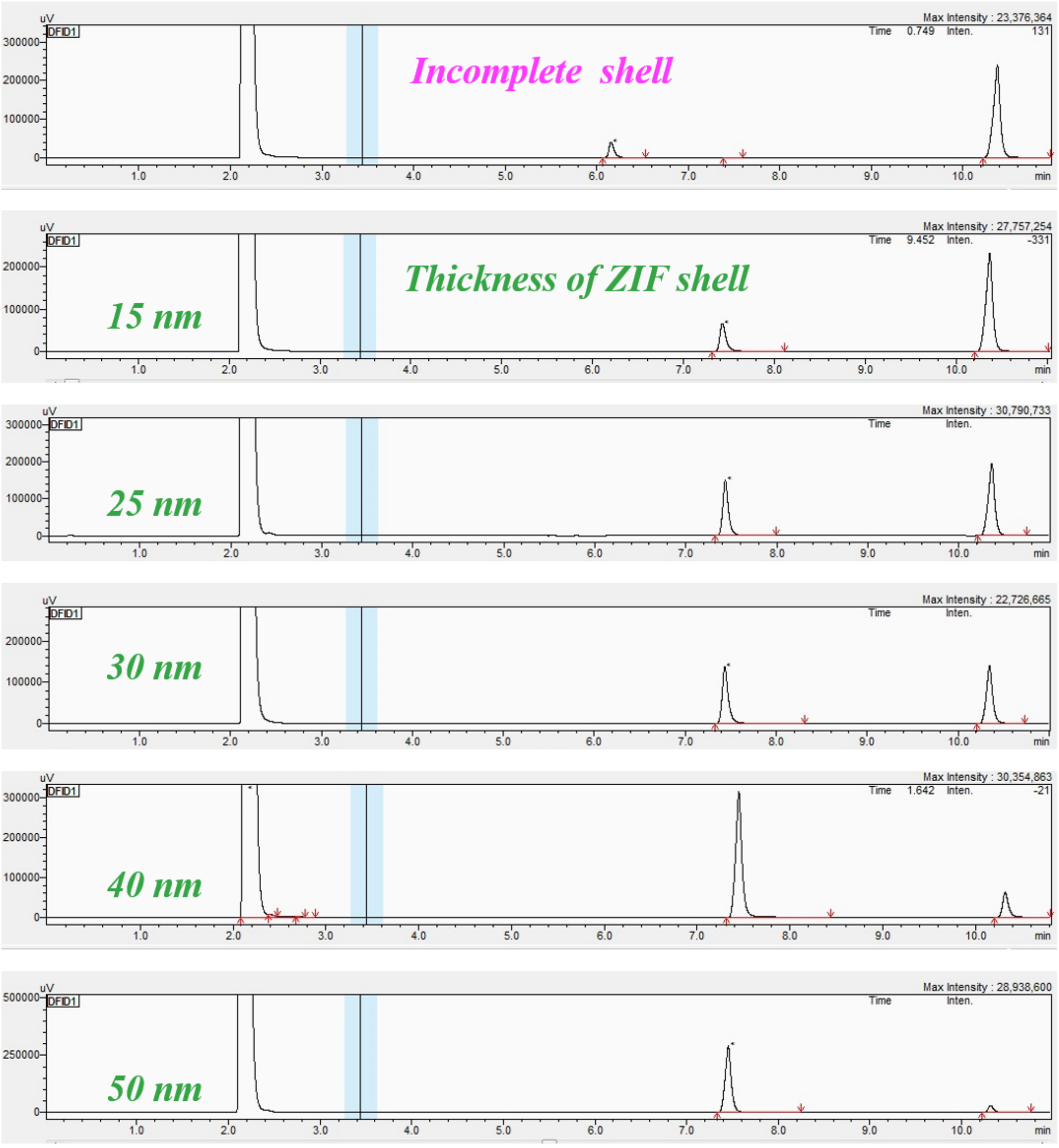

Supplementary Fig. 81 Raw GC data for selective hydrogenation of 5-chloroquinolines by Zn-ZIF-8@Pt@Zn-ZIF-8 (H_2O) with different shell thickness (Error experiment 1).

Solvent: isopropanol
Temperature: 80°C
Reductant: 2 MPa H_2
Time: 200 min
Reactant: 0.5 mmol 5-chloroquinolines

With the increase of shell thickness, the conversion ratio of 5-chloroquine gradually decreases while the selectivity remains stable.

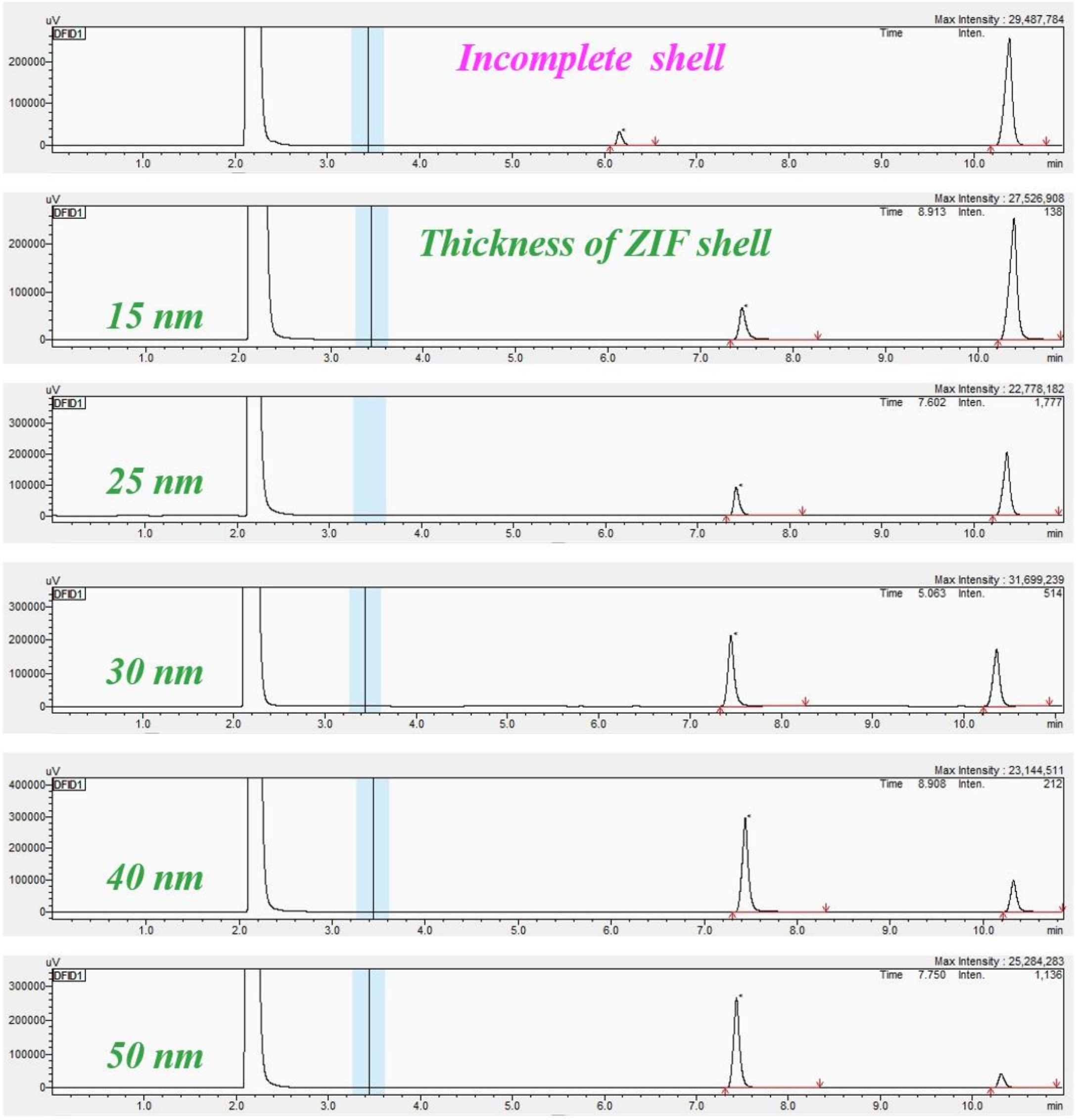

Supplementary Fig. 82 Raw GC data for selective hydrogenation of 5-chloroquinolines by Zn-ZIF-8@Pt@Zn-ZIF-8 (H_2O) with different shell thickness (Error experiment 2).

Solvent: isopropanol
Temperature: 80°C
Reductant: 2 MPa H_2
Time: 200 min
Reactant: 0.5 mmol 5-chloroquinolines

With the increase of shell thickness, the conversion ratio of 5-chloroquine gradually decreases and the selectivity remains stable.

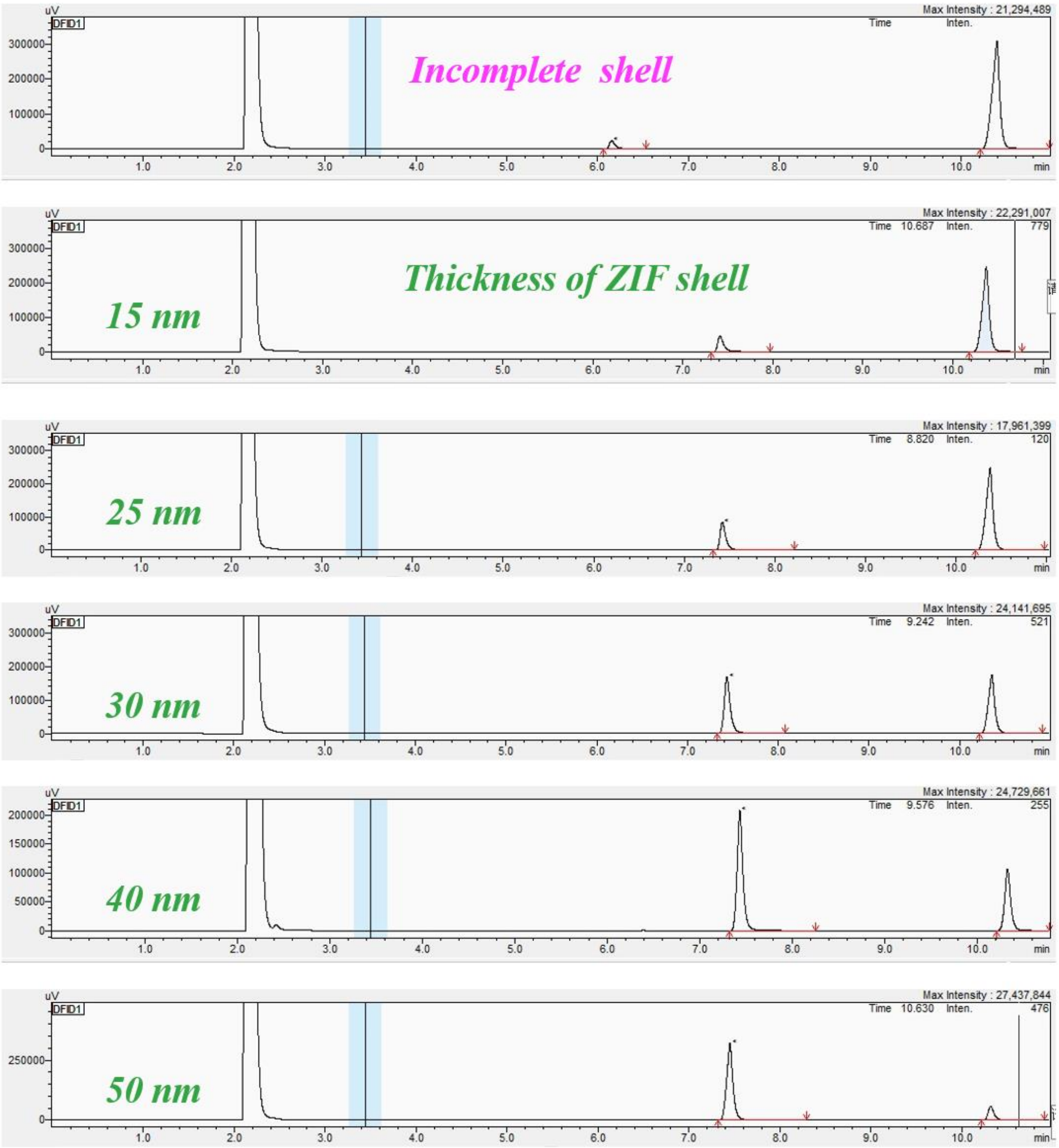

Supplementary Fig. 83 Raw GC data for selective hydrogenation of 5-chloroquinolines by Zn-ZIF-8@Pt@Zn-ZIF-8 (H_2O) with different shell thickness (Error experiment 3).

Solvent: isopropanol
Temperature: 80°C
Reductant: 2 MPa H_2
Time: 200 min
Reactant: 0.5 mmol 5-chloroquinolines

With the increase of shell thickness, the conversion ratio of 5-chloroquine gradually decreases and the selectivity remains stable.
